# MiR-130a-3p Alleviates Inflammatory and Fibrotic Phases of Pulmonary Fibrosis Through Proinflammatory Factor TNF-α and Profibrogenic Receptor TGF-βRII

**DOI:** 10.3389/fphar.2022.863646

**Published:** 2022-03-30

**Authors:** Yan Ding, Yapeng Hou, Yanhong Liu, Tong Yu, Yong Cui, Hongguang Nie

**Affiliations:** ^1^ Department of Stem Cells and Regenerative Medicine, College of Basic Medical Science, China Medical University, Shenyang, China; ^2^ Department of Anesthesiology, the First Hospital of China Medical University, Shenyang, China

**Keywords:** pulmonary fibrosis, miR-130a-3p, NF-κB signaling pathway, inflammatory cytokines, TGF-β/Smad signaling pathway

## Abstract

Pulmonary fibrosis (PF) is a progressive disease characterized by extracellular matrix (ECM) deposition that destroys the normal structure of the lung parenchyma, which is classified into two successive inflammatory and fibrotic phases. To investigate the anti-inflammatory and anti-fibrotic roles of miR-130a-3p in mice with bleomycin (BLM)-induced PF and the underlying mechanism, we performed single-cell RNA-sequencing analysis, which demonstrated that BLM increased/decreased the percentage of macrophages and fibroblasts/epithelial cells in PF lungs, respectively. The differentially expressed genes were enriched in PPAR signaling pathway and lysosome, ECM–receptor interaction and ribosome, and metabolism reaction. Time-course studies demonstrated that the inflammation-related factors increased significantly at day 7 (inflammatory phase), whereas the fibrosis-related factors increased at day 28 (fibrotic phase) after BLM exposure. Meanwhile, miR-130a-3p could ameliorate pulmonary lesions by downregulating the secretion of inflammatory cytokines (IL-1β, IL-6, TNF-α, and TGF-β1) and the deposition of ECM (α-SMA, FN, HYP, and collagen) in the inflammatory and fibrotic phase, respectively. In the LPS-induced inflammatory cell model, the upregulation of miR-130a-3p was mainly achieved by the activation of the NF-κB signaling pathway, which suppressed the proinflammatory factor TNF-α. Comparatively, the TGF-β/Smad signaling pathway was inhibited by miR-130a-3p targeting TGF-βRII in the TGF-β1-deduced fibrotic cell model. The evidence supports that miR-130a-3p exerts an anti-inflammatory and anti-fibrotic effect in BLM-induced PF, implying a potential pharmacological agent in the therapy of PF patients.

## Introduction

Pulmonary fibrosis (PF) is a lethal and nearly cureless pulmonary disease, which is characterized by lung epithelium–mesenchyme transition (EMT), proliferation of fibroblasts, and replacement of functional tissue with the extracellular matrix (ECM) (Meyer 2017; Deng et al., 2020). Despite great advancements in this field, the molecular basis of PF is still not completely understood.

Multiple cell types are involved in the two major development phases of PF, namely, inflammatory (clotting/coagulation and inflammation) and fibrotic (fibroblast migration/proliferation/activation and tissue remodeling). Recent studies have confirmed that macrophages are regarded as central mediators in the inflammatory phase by releasing inflammatory cytokines, such as interleukin (IL)-1β, IL-6, and tumor necrosis factor (TNF)-α, (Liu and Yang 2013; Oishi and Manabe 2018; Li et al., 2019; Xianyuan et al., 2019; Wang et al., 2020; Bignold and Johnson 2021), whereas fibroblasts are considered to be the pivotal effector cells in the fibrotic phase, participating in the deposition of ECM that leads to the progressive decline in lung function (Xie et al., 2018; Peyser et al., 2019). Transforming growth factor-β1 (TGF-β1), a crucial fibrogenic factor, promotes differentiation of fibroblasts to myofibroblasts with higher expression of α-smooth muscle actin (α-SMA) *via* the TGF-β1/Smad signaling pathway and accelerates the accumulation of ECM (Wei et al., 2019; Lv et al., 2020).

Evidence supports the role of miRNAs in inflammatory response and myofibroblast differentiation in the context of PF, among which miR-130a-3p is highly expressed in inflammatory-related lung diseases and significantly reduces the profibrogenic gene expression (Wang et al., 2017; Kuse et al., 2020; Mo et al., 2020; Parzibut et al., 2021). By bioinformatic prediction using target prediction software (TargetScan and miRanda), we find that miR-130a-3p is a potential binding partner for both TNF-α and TGF-βRII, which are crucial to the NF-κB and TGF-β1/Smad signaling pathway, respectively (Su et al., 2015).

Our previous studies demonstrated that TGF-β1-induced proliferation and differentiation of fibroblasts could be inhibited by miR-130a-3p in the MLG2908 cell line, but whether miR-130a-3p can inhibit the progression of PF *in vivo*, especially in different phases of PF has not been established (Liu et al., 2021). The aim of the present study was to explore the anti-inflammatory and anti-fibrotic effects of miR-130a-3p by examining the activity of the NF-κB and TGF-β1/Smad signaling pathways, which would provide a new therapeutic approach to prevent and perhaps even partially reverse the occurrence and progression of PF.

## Materials and Methods

### Acquisition, Filtering, and Processing of Single-Cell RNA Sequence Data

We downloaded the processed single-cell RNA sequence (scRNA-seq) data from the website (https://hmgubox.helmholtz-muenchen.de/f/492f4319237a464f9a28). The dataset contained lung single-cell suspensions from six time points after bleomycin (BLM) treatment (inflammatory phase-days 3, 7, and 10; fibrotic phase-days 14, 21, and 28) and with 29,297 cells after quality control filtering. The Uniform Manifold Approximation and Projection (UMAP) method was used to display cell-type clusters, and differentially expressed genes (DEGs) were calculated by the Wilcox test (FindMarkers function), following which GO and KEGG pathway analysis was performed with default parameters.

### Bleomycin-Induced Pulmonary Fibrosis Model in Mice

Pathogen-free male C57 mice were provided by Beijing SPF Biotechnology Co., Ltd. with the animal certificate number: SYXK (Liao) 2018–0008. All experiments were performed in accordance with the China Medical University Ethics Committee (certificate number: CMU2019088). The optimal time points for the PF mouse model at different phases were established. The mice (weight 20–25 g) were anesthetized by diazepam (17.5 mg kg^−1^, intraperitoneally) followed by ketamine (450 mg kg^−1^, intraperitoneally) 6 min later. BLM was injected intratracheally at the dosage of 1.5 mg/kg (Sigma-Aldrich, St. Louis, MO) or vehicle on day 0, and lung tissues were collected at days 0, 7, 14, 21, and 28 after exposure. For the miR-130a-3p therapeutic effects, miR-130a-3p agomiR (3 nmol, GenePharma, Suzhou, China) was injected intratracheally, followed by BLM (1.5 mg/kg), and 2 nmol miR-130a-3p agomiR was given every 7 days. Body weight was monitored daily, and the lungs were harvested at days 7 and 28 (Chaudhary et al., 2006; Kolb et al., 2020), and 100 mg of lung tissue was used for measuring the content of hydroxyproline (HYP, Jiancheng, Nanjing, China).

### Lung Index and Wet/Dry Weight Ratio

The wet weight was measured immediately after the mouse lungs were removed. The lung index was computed as wet lung weight/body weight. The wet/dry weight ratio was determined after the lung was oven-dried at 60°C for 48 h.

### Differential Cell Count and Proinflammatory Cytokines in Bronchoalveolar Lavage Fluid

Bronchoalveolar lavage fluid (BALF) was collected to assess the severity of lung inflammation after BLM instillation as described previously (Mo et al., 2020). Briefly, 0.3 ml PBS was injected into the lung lobe through a tracheal cannula, and the procedure was repeated three times. The BALF samples were centrifuged at 1,500 rpm for 10 min at 4°C, immediately. The differential cell count was evaluated with Wright–Giemsa stain (Solarbio, Beijing, China), and the proinflammatory cytokine levels in the supernatant were detected by ELISA kit (Neobioscience, Shenzhen, China).

### Histology and Immunofluorescence Assay

Freshly harvested lung tissues were fixed, dehydrated, and embedded in 4% paraformaldehyde, 30% sucrose, and OCT, respectively, and then sectioned at 8 μm thickness. To semi-quantify the histopathologic changes in H&E (Solarbio, Beijing, China) or Masson’s trichrome (Jiancheng, Nanjing, China) staining, the alveolitis and Ashcroft scores were applied to assess the pulmonary alveolitis and fibrosis, respectively (Wang et al., 2021).

For immunofluorescence, the cell membrane was permeabilized by Triton-100 (0.1%) and incubated with an α-SMA primary antibody (1:200, 4°C, overnight, Merck, Darmstadt, Germany) and then with a secondary antibody (1:100, ZSGB-BIO, Beijing, China). The nucleus was stained by DAPI. Finally, the sections were mounted using a fluorescence microscope after dehydration.

### Fluorescence *In Situ* Hybridization

The 8-μm-thick tissue sections were used for fluorescence *in situ* hybridization (FISH) assay (Lindahl et al., 2016; Fan et al., 2021). In brief, the slides were treated with proteinase-K for 20 min at 37°C, incubated in the 20 nM miR-130a-3p probe labeled with CY3 (Sequence: 5′-ATG​CCC​TTT​TAA​CAT​TGC​ACT​G-3′) (GenePharma, Suzhou, China) in the hybridization mixture at 37°C overnight, and the nucleus was stained by DAPI.

### Cell Culture and CCK-8 Assay

MH-S and MRC-5 cells were both purchased from the American Type Culture Collection. The MH-S cells were cultured in RPMI medium (Corning, New York, United States) with 10% fetal bovine serum (FBS, Gibco, New York, United States), 100 IU penicillin, and 100 μg/ml streptomycin in 5% CO_2_–95% air at 37°C. The MRC-5 cells were cultured in MEM medium (Gibco, New York, United States) with 10% FBS, 1% MEM NEAA (Gibco, New York, United States), 1% sodium pyruvate (Gibco, New York, United States), 100 IU penicillin, and 100 μg/ml streptomycin.

For cell transfection, 50–60% confluent cells were transfected with TGF-βRII-siRNA (si-TGF-βRII), miR-130a-3p mimic (Mimic), miR-130a-3p inhibitor (Inhibitor), negative control (NC/Mimic NC/Inhibitor NC, the negative control of si-TGF-βRII/miR-130a-3p mimic/inhibitor), and labeled CY3-miR-130a-3p.

To establish the cell model of the inflammatory and fibrotic phase, the MH-S and MRC-5 cells were treated with LPS (0, 1, 2, 5, 10, and 20 μg/ml) for 12/24 h and TGF-β1 (0, 1, 5, 10, and 20 ng/ml) for 24/48 h, respectively. After treatment, the viability of cells was measured by a CCK-8 kit (Biosharp, Guangzhou, China) according to the manufacturer’s protocol.

### Western Blot Assay

The protein lysates were separated on SDS-PAGE gels and then transferred to PVDF membranes (Invitrogen, Waltham, United States). The membranes were blocked in 5% BSA for 1 h and incubated with following primary antibodies: TNF-α (1:1,000, Santa Cruz, California, United States), P65, IκB, and p-IκB (1:1,000, Abmart, Shanghai, China), E-cadherin (1:1,000, Affinity, Nanjing, China), SMAD4 (1:1,000, Cell Signaling Technology, Danvers, United States), α-SMA (1:1,000, Merck, Darmstadt, Germany), FN (1:1,000, Abcam, Cambridge, United Kingdom), TGF-βRII, SMAD2/3, p-SMAD2/3 (1:1,000, Elabscience, Wuhan, China), and β-actin (1:1,000, Santa Cruz, California, United States) overnight at 4°C. The membranes were then washed three times with TBST for 10 min and incubated with secondary antibody (1:5,000, ZSGB-BIO, Beijing, China). The protein bands were visualized using ECL reagents.

### Quantitative Real-Time PCR

Total RNA was isolated using TRIzol (Invitrogen, Waltham, United States). The cDNAs of RNA and miRNAs were synthesized by a synthesis kit (TaKaRa, Kusatsu, Japan). Quantitative real time-polymerase chain reaction (qRT-PCR) was then carried out by SYBR Premix Ex Taq II (TaKaRa, Kusatsu, Japan) in the Applied Biosystems 7,500 system. The relative expression levels were calculated by the 2^−ΔΔCT^ method and normalized to the GAPDH (for mRNAs) or U6 (for miRNAs). The external replicates are at least three. All the primers are shown in Supplementary Table S1.

### Dual Luciferase Reporter Gene Assay

Dual luciferase reporter assay (Vazyme, Nanjing, China) was performed to determine the firefly and Renilla luciferase activities in NC and miR-130a-3p mimic with TGF-βRII-3′UTR WT or TGF-βRII-3′UTR MUT reporter plasmids (GenePharma, Suzhou, China).

### Statistical Analysis

Data were presented as the mean ± SE. We evaluated the power of the sample size first to meet *p* < 0.05. After the data passed the Shapiro–Wilk and Levene tests, the differences among the groups were tested by one-way analysis of variance (ANOVA) followed by Bonferroni’s test. If not, the differences between the groups were tested by Mann–Whitney *U*-test. Statistical significance was accepted at *p* < 0.05. All data analysis was performed with Origin 8.0.

## Results

### Differential Gene Expression Analysis

**FIGURE 1:**
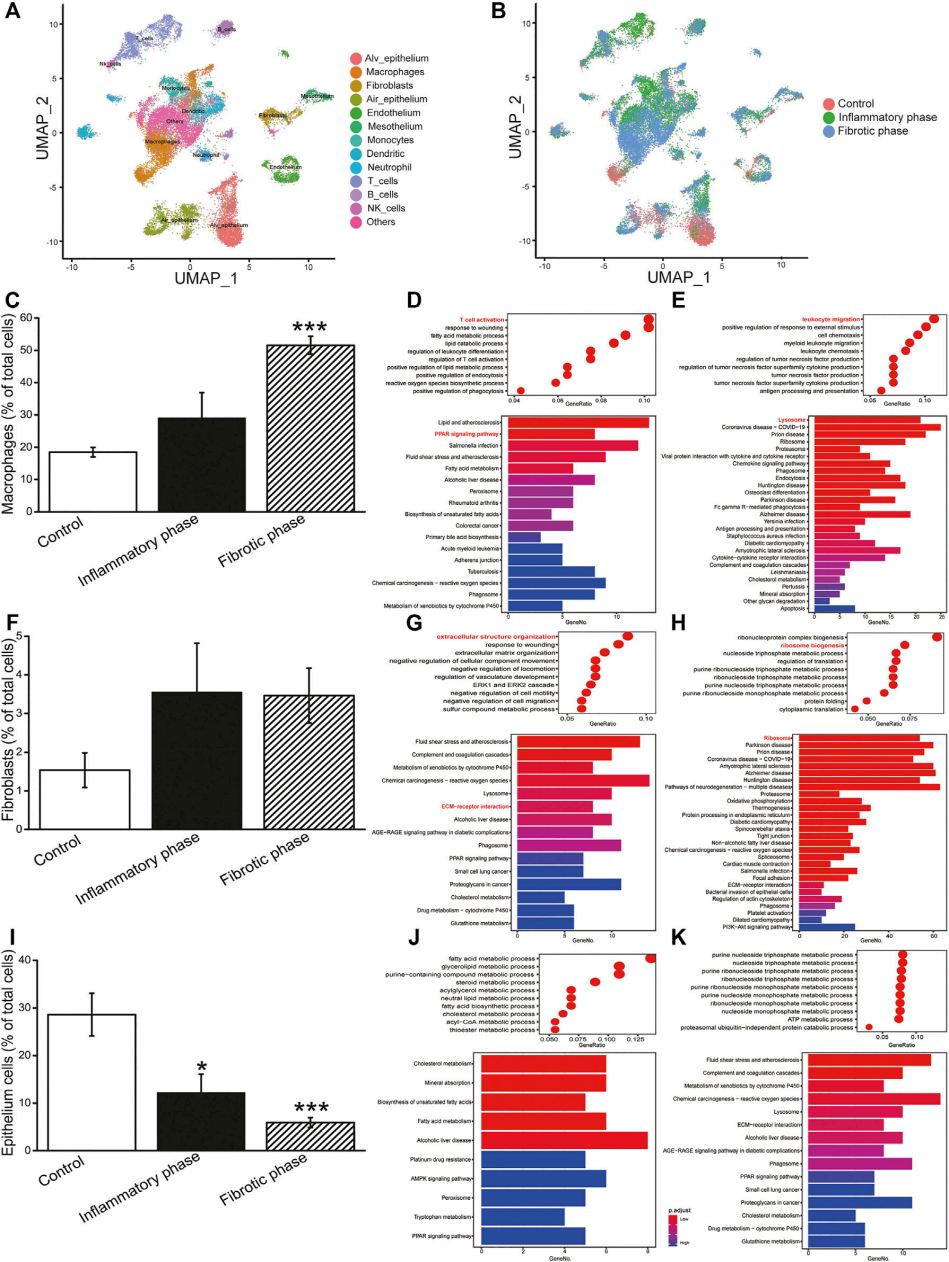
ScRNA-seq analysis of BLM-induced PF. **(A)** Identified cell types in UMAP. **(B)** Cells split by inflammatory and fibrotic phases. **(C)** Percentage of macrophages. Bubble/bar plot showing GO/KEGG enrichment analysis results of the **(D)** downregulated and **(E)** upregulated genes in macrophages. **(F)** Percentage of fibroblasts. Bubble/bar plot showing GO/KEGG enrichment analysis results of the **(G)** downregulated and **(H)** upregulated genes in fibroblasts. **(I)** Percentage of epithelial cells. Bubble/bar plot showing GO/KEGG enrichment analysis results of the **(J)** downregulated and **(K)** upregulated genes in epithelial cells. *p < 0.05, ***p < 0.001, versus the control group, by the Mann–Whitney *U* test.

To describe the cellular heterogeneity and the changes of gene expression networks in BLM-induced PF, we analyzed the scRNA-seq dataset and identified 13 cell-type identities that were manually annotated using canonical marker genes (Figure 1A). Most cell clusters contained cells from all conditions, whereas the number of cells in clusters varied among groups (Figure 1B). Therefore, we analyzed the cell count and revealed that BLM decreased/increased the percentage of epithelial cells/macrophages, fibroblasts, and myofibroblasts in the PF lung, respectively (Figures 1C,F,I; Supplementary Figure S1).

The upregulated and downregulated DEGs in different cells were then subjected to GO enrichment analysis (Figures 1D,E,G,H,J,K). The results revealed that the most significant DEGs in macrophages, fibroblasts, and epithelial cells were enriched in inflammatory, ECM deposition, and metabolism-related biological processes, respectively.

To investigate the functional pathway in different type cells, we performed KEGG enrichment analysis. As shown in Figures 1D,E,G,H,J,K, the DEGs of macrophages, fibroblasts, and epithelial cells were enriched in PPAR signaling pathway and lysosome, ECM–receptor interaction and ribosome, and metabolism reaction, respectively. Therefore, we investigated the activity of the NF-κB and TGF-β1/Smad signaling pathway, which was regulated by PPAR pathway and ECM, respectively.

### Bleomycin Upregulated Inflammatory Cytokines and Fibrosis-Related Genes

**FIGURE 2:**
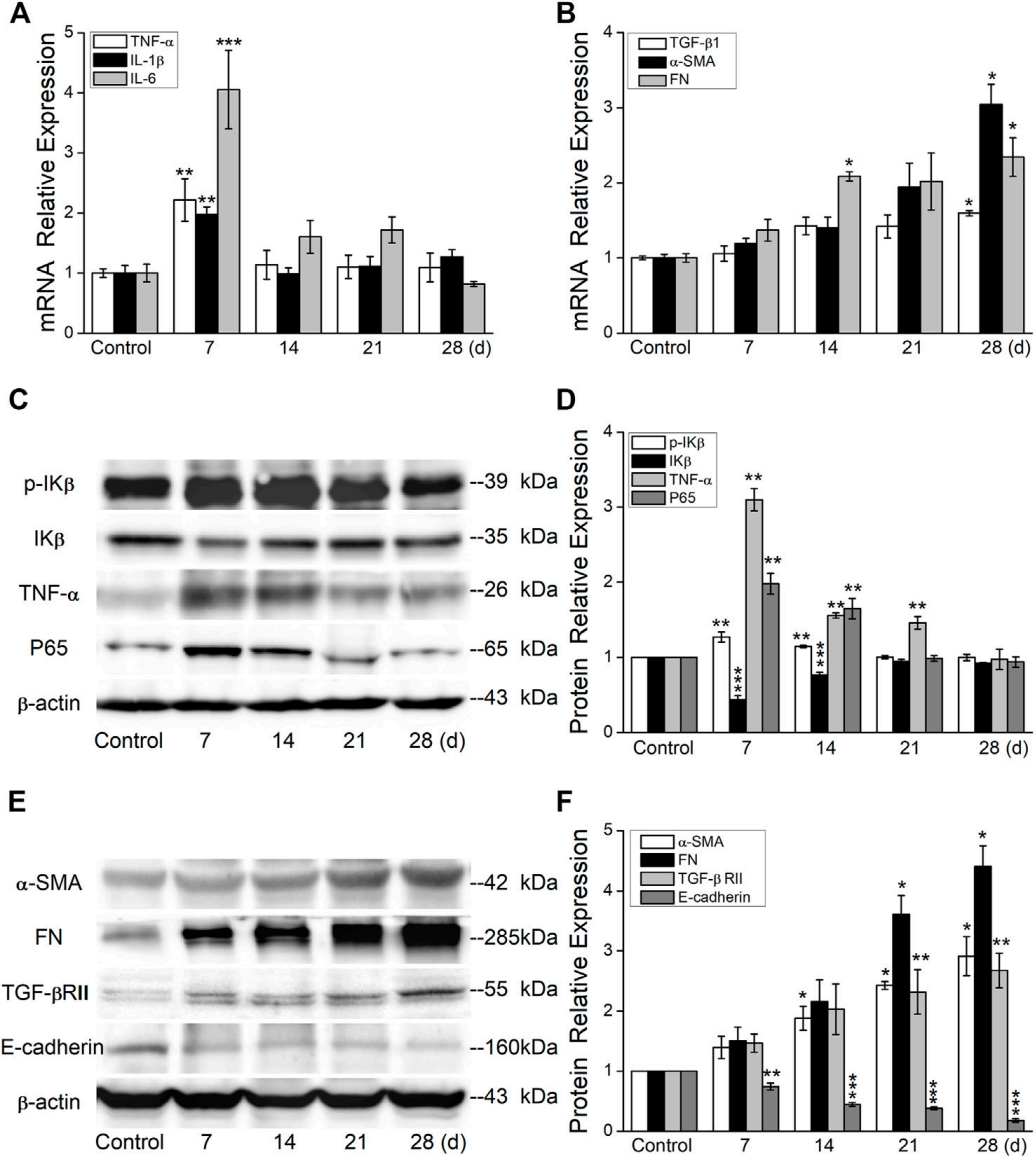
BLM induces the expression of inflammation- and fibrosis-related protein. **(A)** qRT-PCR quantitation of mRNA expressions of inflammation-related genes. **p < 0.01, ***p < 0.001, -versus the control group, one-way ANOVA followed by Bonferroni’s test, *n* = 3–6. **(B)** qRT-PCR quantitation of mRNA expressions of fibrosis-related genes. *p < 0.05, versus the control group, by the Mann–Whitney *U* test, *n* = 3–6. **(C-D)** Representative and statistical data of inflammation-related proteins. **p < 0.01, ***p < 0.001, versus the control group, by the Mann–Whitney *U* test, *n* = 4–5. **(E-F)** Representative and statistical data of fibrosis-related proteins. *p < 0.05, **p < 0.01, ***p < 0.001, versus the control group, by the Mann–Whitney *U* test, *n* = 3–5.

To establish an optimal time point for inflammatory and fibrotic phases, we first performed a time-course study in the BLM-induced PF model. As shown in Figures 2A,B, the mRNA expression levels of proinflammatory cytokine IL-1β, IL-6, and TNF-α were upregulated significantly at day 7, whereas the fibrosis-related genes increased at day 28 after BLM exposure.

Consistent with the aforementioned results at the mRNA level, our data also demonstrated that the inflammation-related proteins increased significantly at day 7 (Figures 2C,D), whereas the fibrosis-related proteins increased at day 28 after BLM exposure (Figures 2E,F). The increased and continued expression of proinflammatory cytokines and fibrosis-related genes in mouse lungs after BLM exposure suggests that BLM can induce persistent inflammatory and profibrotic responses in mouse lungs, signifying a shift from inflammatory response to fibrotic response.

### Dual Changes of MiR-130a-3p Expression in Inflammatory and Fibrotic Phases

**FIGURE 3:**
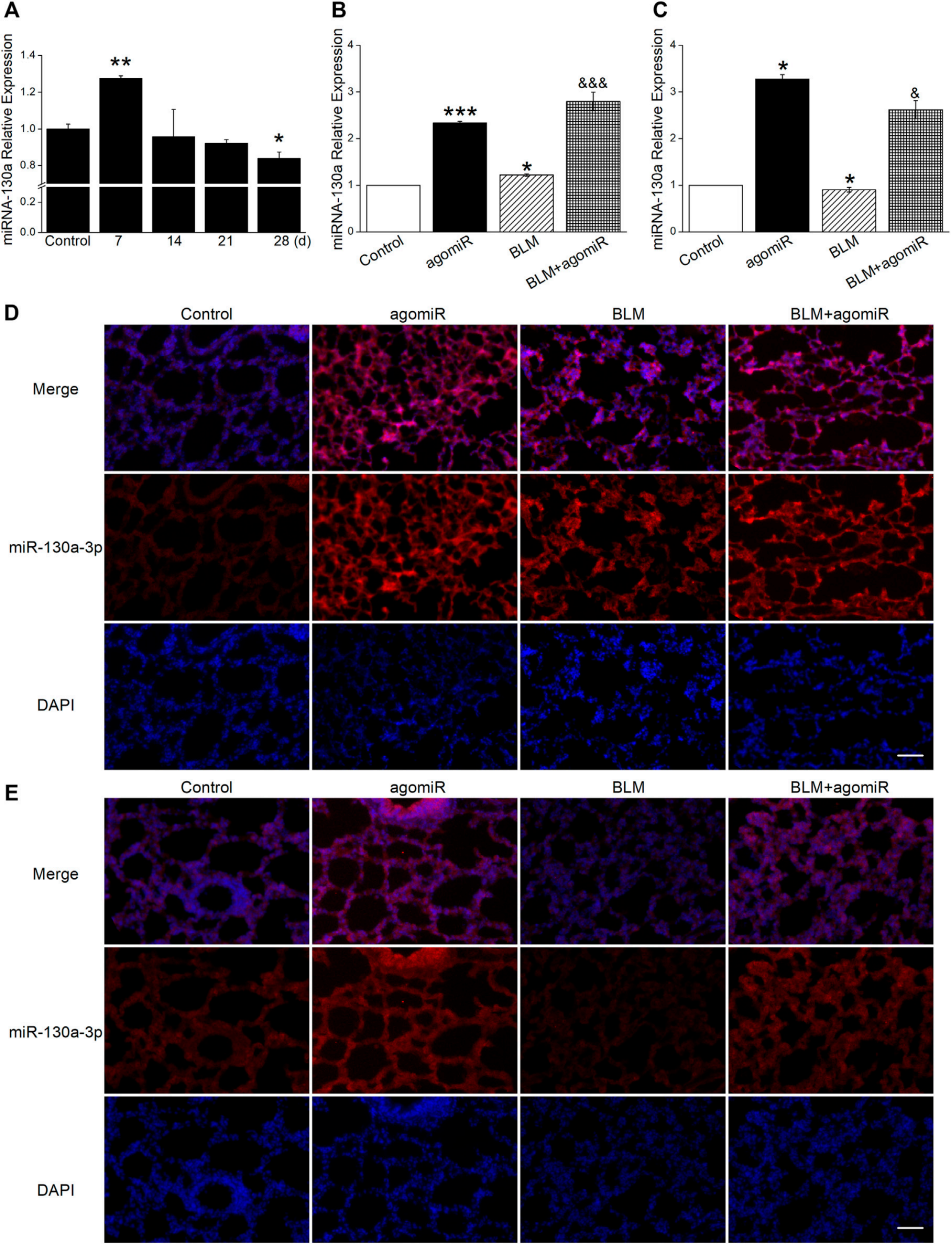
MiR-130a-3p expression correlates with inflammatory and fibrotic phases. **(A)** qRT-PCR quantitation of miR-130a-3p expression at different times after BLM treatment. *p < 0.05, **p < 0.01, versus the control group, by the Mann–Whitney *U* test, *n* = 3–4. **(B)** qRT-PCR quantitation of miR-130a-3p expression in the inflammatory phase after BLM and/or miR-130a-3p agomiR treatment. *p < 0.05, ***p < 0.001, versus the control group; ^&&&^ *p* < 0.001, versus the BLM group, one-way ANOVA followed by Bonferroni’s test, *n* = 3–4. **(C)** qRT-PCR quantitation of miR-130a-3p expression in the fibrotic phase after BLM and/or miR-130a-3p agomiR treatment. *p < 0.05, versus the control group; ^&^ *p* < 0.05, versus the BLM group, by the Mann–Whitney *U* test, *n* = 4–5. FISH analysis of miR-130a-3p in lung tissues from the **(D)** inflammatory phase and **(E)** fibrotic phase. miR-130a-3p-positive (red) and DAPI (blue). Scale bar = 50 µm.

The dynamic changes of miR-130a-3p showed that it was highly expressed at day 7 post BLM administration whereas decreased at day 28, illustrating that the miR-130a-3p level was phase-dependent in the BLM-induced PF model (Figure 3A).

To further evaluate miR-130a-3p expression in miR-130a-3p agomiR therapy, lung tissues from different phases were examined by qRT-PCR and FISH methods. After injection of agomiR, the miR-130a-3p mRNA level was significantly increased (Figures 3B,C), which was further supported by the FISH results (Figures 3D,E).

### MiR-130a-3p Suppressed Pulmonary Inflammation and Collagen Deposition

**FIGURE 4:**
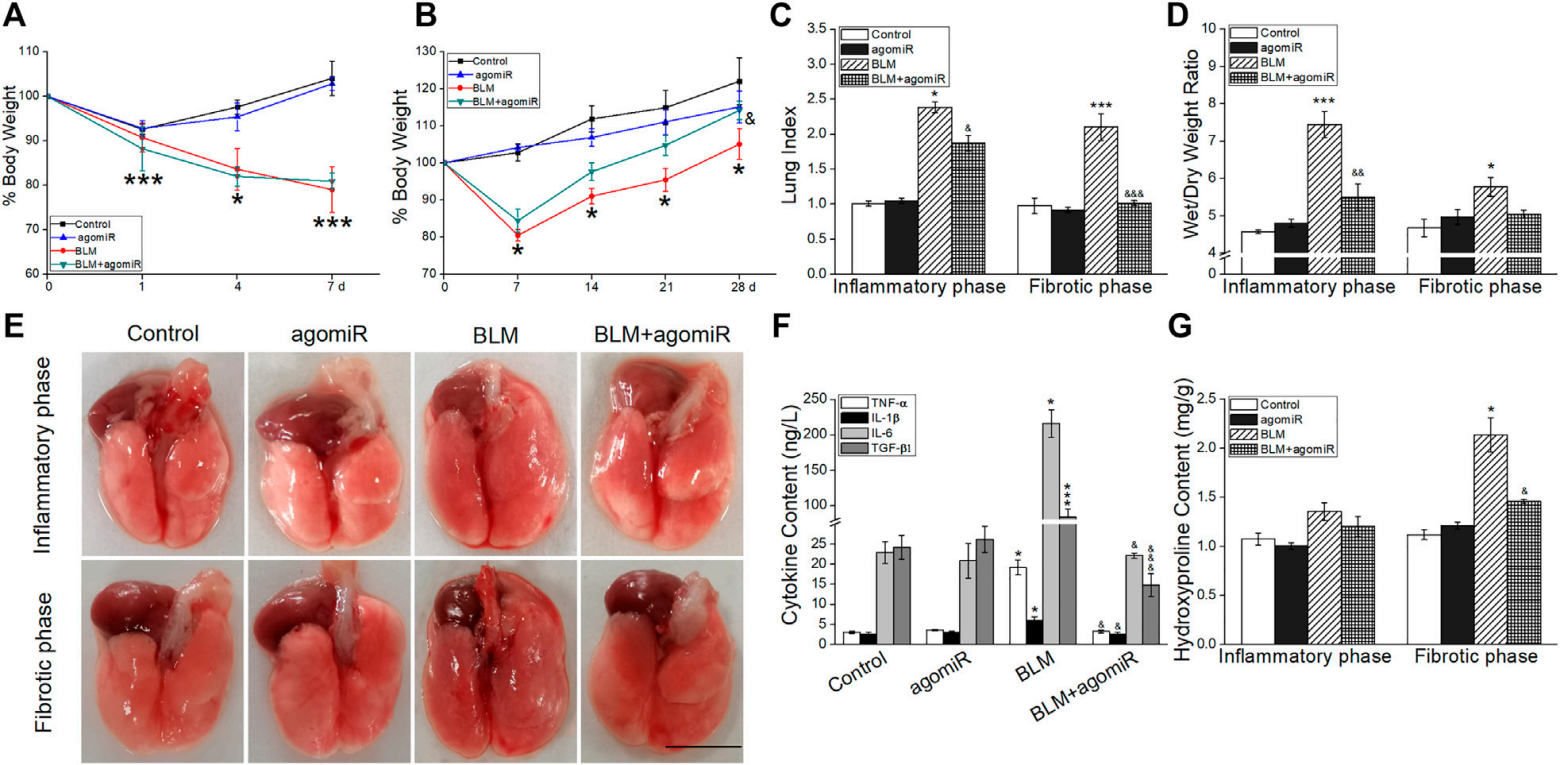
Effect of miR-130a-3p agomiR on pulmonary inflammation and collagen deposition. **(A-B)** Mouse body weight changes in the course of the inflammatory phase or fibrotic phase after BLM induction. *p < 0.05, ***p < 0.001, versus the control group; ^&^ *p* < 0.05, versus the BLM group, by the Mann–Whitney *U* test, *n* = 3–5. **(C)** Lung index (%). **(D)** Wet/dry weight ratio. *p < 0.05, ***p < 0.001, versus the control group; ^&^ *p* < 0.05, ^&&^ *p* < 0.01, ^&&&^ *p* < 0.001, versus the BLM group, one-way ANOVA followed by Bonferroni’s test, *n* = 3–5. **(E)** Representative photographs of whole lungs from all the experimental groups. Scale bar = 1 cm. **(F)** TNF-α, IL-1β, and IL-6 levels in BALF of the inflammatory phase. *p < 0.05, versus the control group; ^&^ *p* < 0.05, versus the BLM group, by the Mann–Whitney *U* test, n = 4–5. TGF-β1 level in BALF of the fibrotic phase. ***p < 0.001, versus the control group; ^&&&^ *p* < 0.001, versus the BLM group, one-way ANOVA followed by Bonferroni’s test, *n* = 4–5. **(G)** HYP levels in lung tissue of two phases. *p < 0.05, versus the control group; ^&^ *p* < 0.05, versus the BLM group, by the Mann–Whitney *U* test, *n* = 3–5.

There was a significant weight loss after intratracheal instillation by BLM compared with the mice in the control group, especially in the 7 days post administration (Figures 4A,B). However, miR-130a-3p was able to reverse the reduction of weight and actually facilitated weight gain in BLM-induced fibrotic phase mice. In contrast, the lung index and wet/dry weight ratio were increased, suggesting that BLM could cause pulmonary edema damage in the inflammatory phase, which was alleviated by miR-130a-3p (Figures 4C,D).

After lung collection, the appearance of BLM-induced lungs was swollen and hyperemic in the inflammatory phase, whereas lungs were enlarged and showed diffuse hemorrhage in the fibrotic phase. The miR-130a-3p therapeutic lungs showed an improved aspect compared with the BLM group (Figure 4E).

As shown in Figure 4F, miR-130a-3p could inhibit the increase of inflammatory factors (TNF-α, IL-6, and IL-1β) in the inflammatory phase, identical with the proportion of lymphocytes and neutrophils in BALF (Supplementary Figure S2). Intriguingly, the high levels of fibrogenic factor (TGF-β1) and HYP were mitigated by miR-130a-3p administration, indicating that miR-130a-3p inhibited collagen deposition in the BLM-induced fibrotic phase (Figures 4F,G).

### MiR-130a-3p Offset Bleomycin-Induced Structural Damage

**FIGURE 5:**
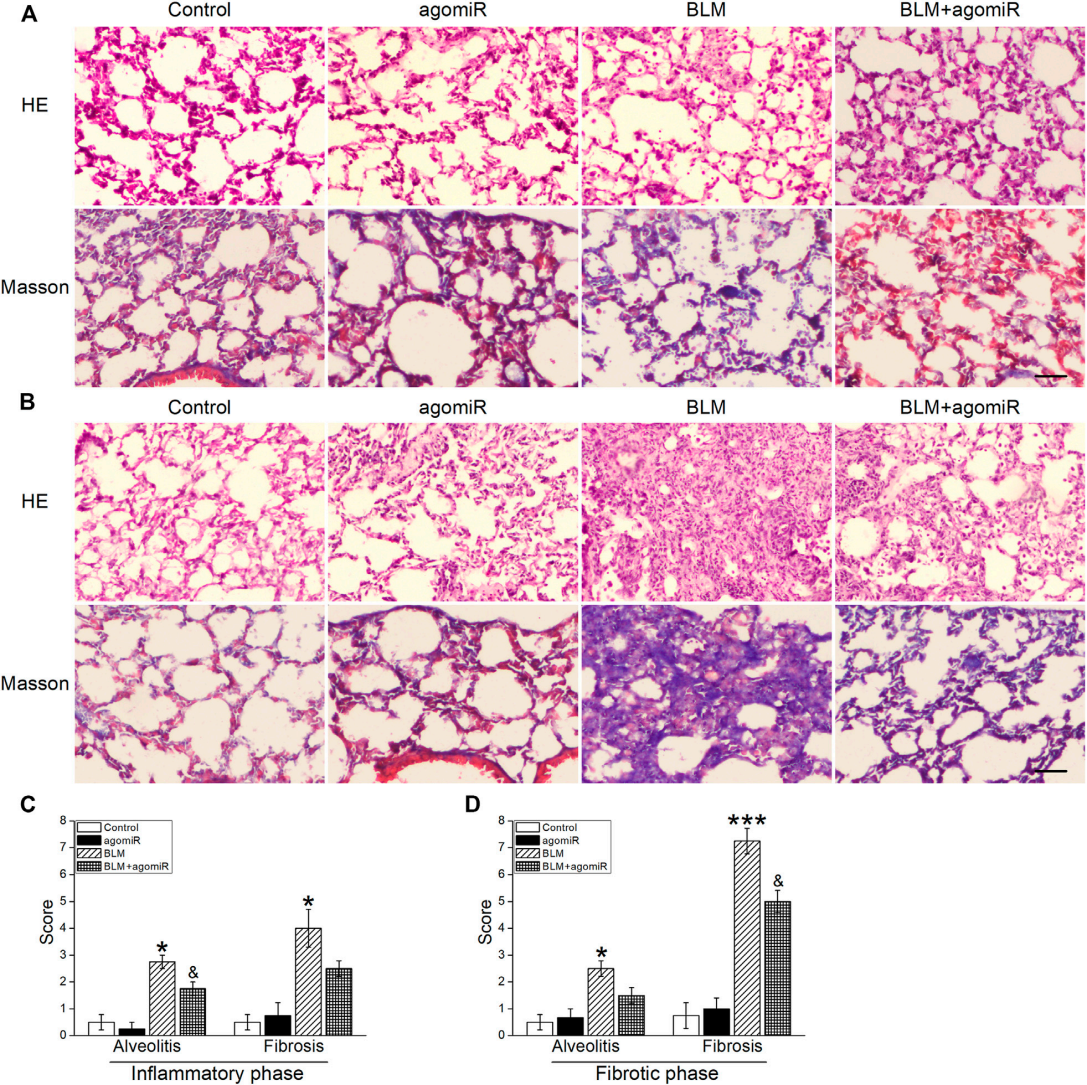
MiR-130a-3p agomiR offsets BLM-induced structural damage in the mouse lung. Pathologic change of lung tissues evaluated by H&E and Masson’s trichrome staining in the **(A)** inflammatory phase and **(B)** fibrotic phase after BLM induction. Scale bar = 50 μm. Alveolitis and fibrosis scores of the lung sections in the **(C)** inflammatory phase and **(D)** fibrotic phase. *p < 0.05, ***p < 0.001, versus the control group; ^&^ *p* < 0.05, versu*s* the BLM group, by the Mann–Whitney *U* test, *n* = 4–5.

As shown in HE staining (Figures 5A,B), the PF mice developed marked lung injury in the inflammatory phase, manifested by the thickened alveolar septum with a large inflammatory cell infiltration and destructive alveolar structure, suggesting a development of pulmonary edema. As expected, in the fibrotic phase, lung injury got worse in the BLM mice, manifested by disarrangement of alveolar architecture, with particularly serious thickness of the alveolar wall and severe interstitial fibrosis. Additionally, quantitative data for the alveolitis score in the lung were in accordance with the histological observations, while the miR-130a-3p treatment markedly mitigated these events (Figures 5C,D).

To further ascertain whether miR-130a-3p inhibits collagen generation, Masson's trichrome staining was used. In BLM-stimulated mice, especially in the fibrotic phase, the lung tissue exhibited a significant increase in both the collagen fibers and fibrosis scores, illustrating that serious lung fibrosis was induced by BLM, whereas miR-130a-3p significantly attenuated the increase of these parameters (Figures 5A–D).

### MiR-130a-3p Reduced the Expression of Inflammation and Fibrosis-Related Factors

**FIGURE 6:**
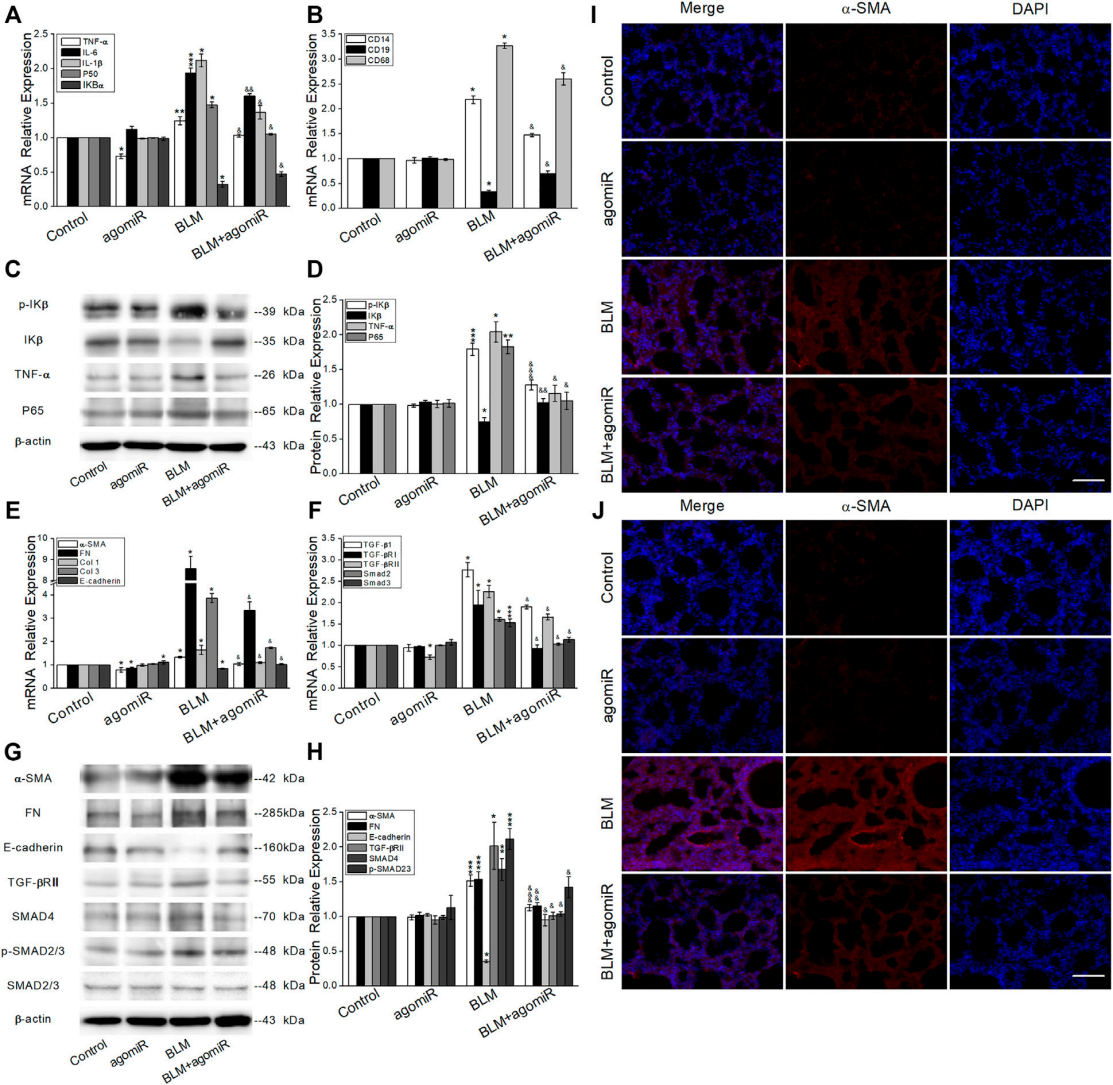
MiR-130a-3p reduces the expression of inflammatory- and fibrotic-related factors. **(A-B)** qRT-PCR quantitation of mRNA expressions of inflammation-related genes. *p < 0.05, **p < 0.01, ***p < 0.001, versus the control group; ^&^ *p* < 0.05, ^&&^ *p* < 0.01, versus the BLM group, by the Mann–Whitney *U* test, *n* = 3–5. **(C-D)** Representative and statistical data of NF-κB signal pathway protein levels. *p < 0.05, **p < 0.01, ***p < 0.001, versus the control group; ^&^ *p* < 0.05, ^&&^ *p* < 0.01, ^&&&^ *p* < 0.001, versus the BLM group, by the Mann–Whitney *U* test, *n* = 3–5. **(E-F)** qRT-PCR quantitation of mRNA expressions of fibrosis-related genes. *p < 0.05, ***p < 0.001, versus the control group; ^&^ *p* < 0.05, versus the BLM group, by the Mann–Whitney *U* test, *n* = 3–5. **(G-H)** Representative and statistical data of TGF-β/Smad signal pathway protein levels. *p < 0.05, **p < 0.01, ***p < 0.001, versus the control group; ^&^ *p* < 0.05, ^&&^ *p* < 0.01, ^&&&^ *p* < 0.001, versus the BLM group, by the Mann–Whitney *U* test, *n* = 3–5. Immunofluorescence for α-SMA in the **(I)** inflammatory phase and **(J)** fibrotic phase. α-SMA-positive (red) and DAPI (blue). Scale bar = 50 μm.

To investigate whether the NF-κB signaling pathway was related to the inflammatory phase and clarify the role of miR-130a-3p, we applied qRT-PCR (Figures 6A,B) and Western blot (Figures 6C,D), both of which showed higher expression of P65, TNF-α, IL-1β, IL-6, p-IκB, and CD14/68 and lower expression of IκB and CD19 in the BLM group, whereas after miR-130a-3p treatment, the expression of the aforementioned markers was partly reversed.

We next investigated the expression of fibrosis-related factors. As expected, ECM proteins (α-SMA, fibronectin (FN), and Col 1/3) markedly increased, whereas E-cadherin decreased at the mRNA level (Figure 6E). Meanwhile, TGF-β1/Smad signaling molecules (TGF-βRII, p-SMAD2/3, SMAD2/3, and SMAD4) were upregulated in BLM-induced lungs (Figure 6F). The aforementioned results were also verified at the protein level, whereas miR-130a-3p showed an opposite effect on BLM-induced PF (Figures 6G,H).

α-SMA is a recognized biomarker of myofibroblast activation. Consistent with the aforementioned results, immunofluorescent staining assay also demonstrated more α-SMA staining (red staining) in the alveolar and interstitial space of BLM-induced lungs than that of miR-130a-3p treatment, especially in the fibrotic phase (Figures 6I,J).

### MiR-130a-3p Suppressed Tumor Necrosis Factor-α Production in the Inflammatory Phase

**FIGURE 7:**
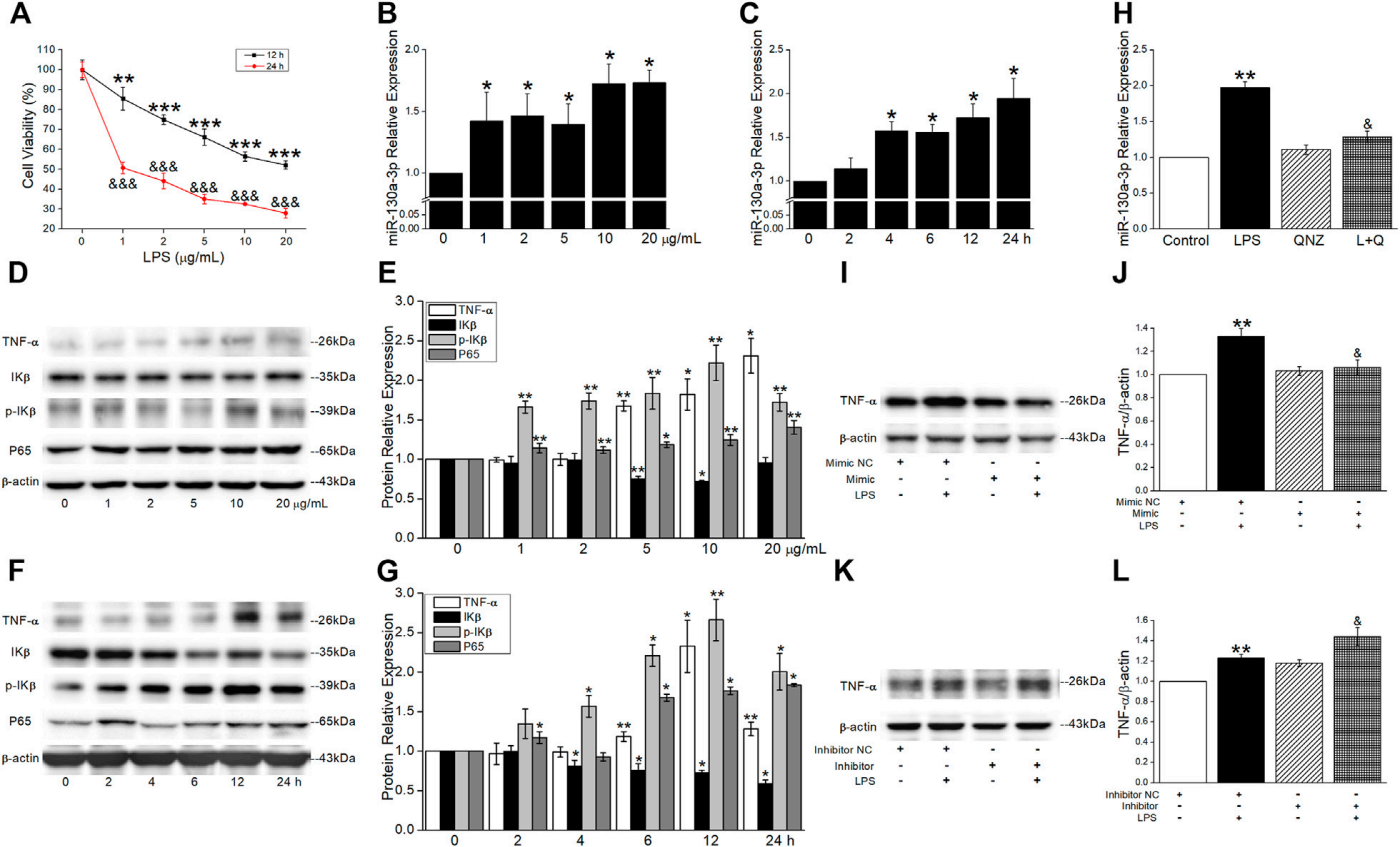
MiR-130a-3p suppresses TNF-α production in the inflammatory phase. **(A)** Relative cell viability was measured by CCK-8 assay, and the data for 0 μg/ml were set to 100%. **p < 0.01, ***p < 0.001, versus 0 μg/ml (12 h); ^&&&^ *p* < 0.001, versus 0 μg/ml (24 h), one-way ANOVA followed by Bonferroni’s test, *n* = 3–4. **(B-C)** qRT-PCR quantitation of miR-130a-3p expression under concentration- and time-dependent LPS treatment. *p < 0.05, versus 0 μg/ml or 0 h, by the Mann–Whitney *U* test, *n* = 4. Representative and statistical data of NF-κB signal pathway protein levels under **(D-E)** concentration- and **(F-G)** time-dependent LPS treatment. *p* < 0.05, **p < 0.01, versus 0 μg/ml or 0 h, by the Mann–Whitney *U* test, *n* = 3–5. MH-S cells were treated with 11 nM of NF-κB inhibitor (QNZ) and 10 μg/ml of LPS for 12 h. **(H)** qRT-PCR quantitation of miR-130a-3p expression. **p < 0.01, versus the control group; ^&^ *p* < 0.05, versus the LPS group, by the Mann–Whitney *U* test, *n* = 4. **(I-J)** Representative and statistical data of TNF-α protein after LPS administration and/or miR-130a-3p mimic transfection. **(K-L)** Representative and statistical data of TNF-α protein after LPS administration and/or miR-130a-3p inhibitor transfection. **p < 0.01, versus the Mimic/Inhibitor NC group; ^&^ *p* < 0.05, versus the Mimic/Inhibitor NC + LPS group, by the Mann–Whitney *U* test, *n* = 4-7.

To determine the cell viability in an inflammatory cell model, the MH-S cells were treated with different concentrations (0, 1, 2, 5, 10, and 20 μg/ml) of LPS. The cell viability was inhibited in a concentration-dependent manner and decreased to about 60% after LPS administration at 10 μg/ml for 12 h (Figure 7A). The levels of miR-130a-3p were rapidly increased in a concentration- and time-dependent manner after LPS treatment in the MH-S cells (Figures 7B,C). To investigate the relationship between miR-130a-3p and inflammatory-related genes, we found that LPS upregulated the expression of TNF-α, p-IKβ, and P65 and downregulated the expression of IKβ in a concentration- and time-dependent manner (Figures 7D–G). Based on the previously mentioned results, the MH-S cells treated with LPS at 10 μg/ml for 12 h were chosen for further experiments.

To further identify that NF-κB/p65 activation following LPS may play a crucial role in production surplus of miR-130a-3p, we measured the levels of miR-130a-3p in response to LPS in the presence of a NF-κB inhibitor, quinazoline (QNZ, 11 nM) in MH-S cells. The qRT-PCR analysis revealed that the increase of miR-130a-3p expression induced by LPS was offset by the treatment of QNZ, which indicated that LPS induced miR-130a-3p expression through NF-κB (Figure 7H). To make sure whether TNF-α protein was controlled by miR-130a-3p, we applied miR-130a-3p mimic and found that the protein expression of TNF-α induced by LPS was reduced after miR-130a-3p administration whereas reversed by miR-130a-3p inhibitor (Figure 7I–L).

In brief, the aforementioned results uncovered a regulatory mechanism between NF-κB and miR-130a-3p, which controlled TNF-α homeostatic production after NF-κB activation in alveolar macrophages.

### Effects of MiR-130a-3p on the Proliferation and Differentiation in Fibrotic Phase

**FIGURE 8:**
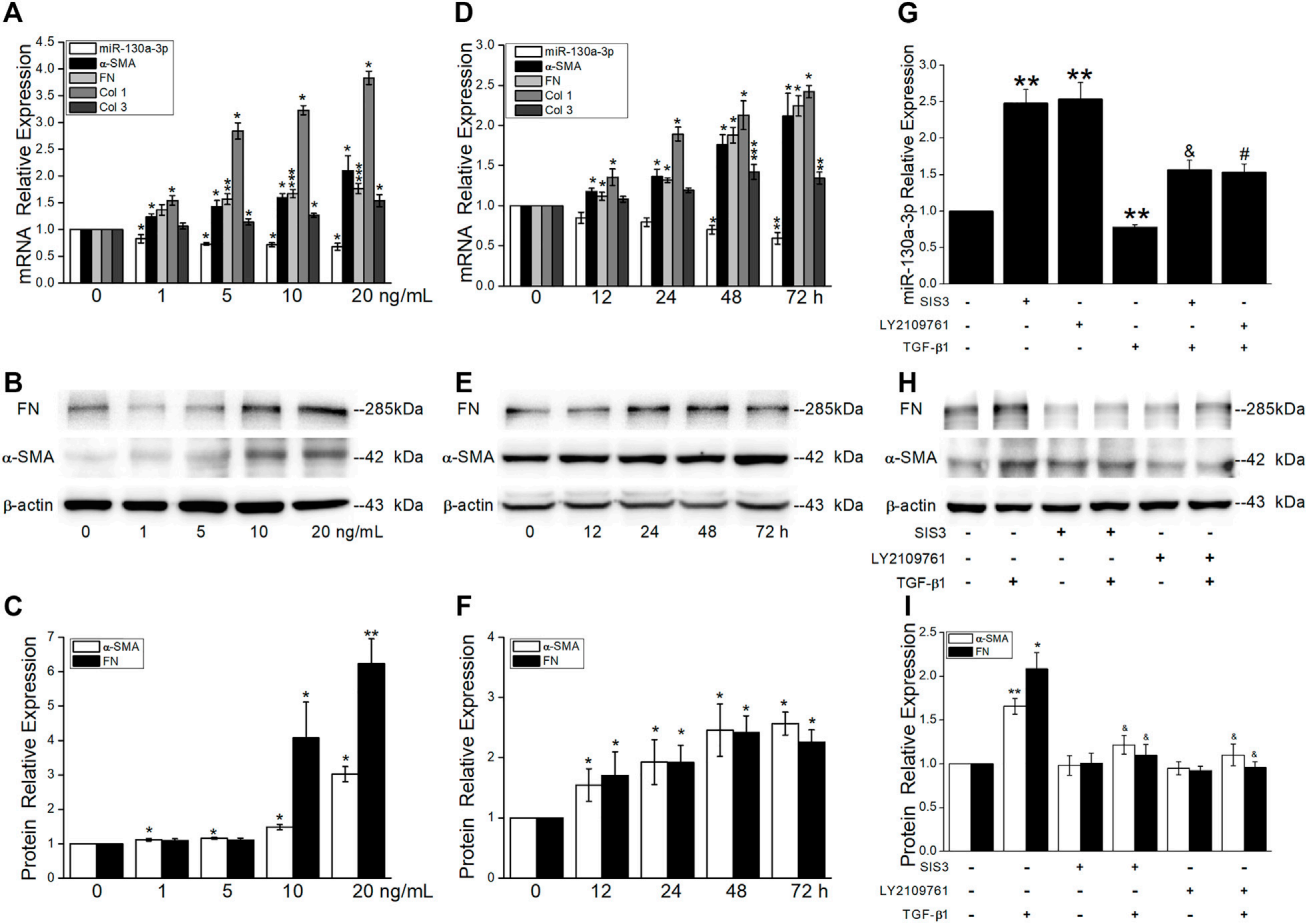
TGF-β1 reduces miR-130a-3p expression via Smad3 signaling pathways. MRC-5 cells were stimulated with various concentrations of TGF-β1 for 48 h or with 5 ng/ml of TGF-β1 for different periods of time. **(A,D)** qRT-PCR quantitation of mRNA expressions of miR-130a-3p and fibrosis-related genes. **(B-C,E-F)** Representative and statistical data of α-SMA and FN proteins. *p < 0.05, **p < 0.01, ***p < 0.001, versus 0 ng/ml or 0 h, by the Mann–Whitney *U* test, *n* = 3–5. MRC-5 cells were pretreated with 10 μM of TGF-βRII inhibitor (LY2109761), Smad3 inhibitor (SIS3), or control (DMSO) for 30 min and then stimulated with 10 ng/ml of TGF-β1 for 24 h. **(G)** qRT-PCR quantitation of mRNA expression of miR-130a-3p. **p < 0.01, versus the control group; ^&^ *p* < 0.05, versus the SISI3 group; ^#^ *p* < 0.05, versus the LY2109761 group, by the Mann–Whitney *U* test, *n* = 4–6. **(H–I)** Representative and statistical data of α-SMA and FN proteins. *p < 0.05, **p < 0.01, versus the control group; ^&^ *p* < 0.05, versus the TGF-β1 group, by the Mann–Whitney *U* test, *n* = 4-5.

TGF-β1, the most critical fibrogenic factor, is involved in the development of PF. Consistent with the upregulation of α-SMA and FN at the protein level, the qRT-PCR results showed a concentration- and time-dependent increase of fibrotic marker expression and decrease of miR-130a-3p (Figures 8A–F). Of note, pretreatment of MRC-5 cells with TGF-βRII inhibitor LY2109761 and/or Smad3 inhibitor SIS3 partially blocked TGF-β1-induced expression of α-SMA and FN while attenuated miR-130a-3p expression (Figures 8G–I).

**FIGURE 9:**
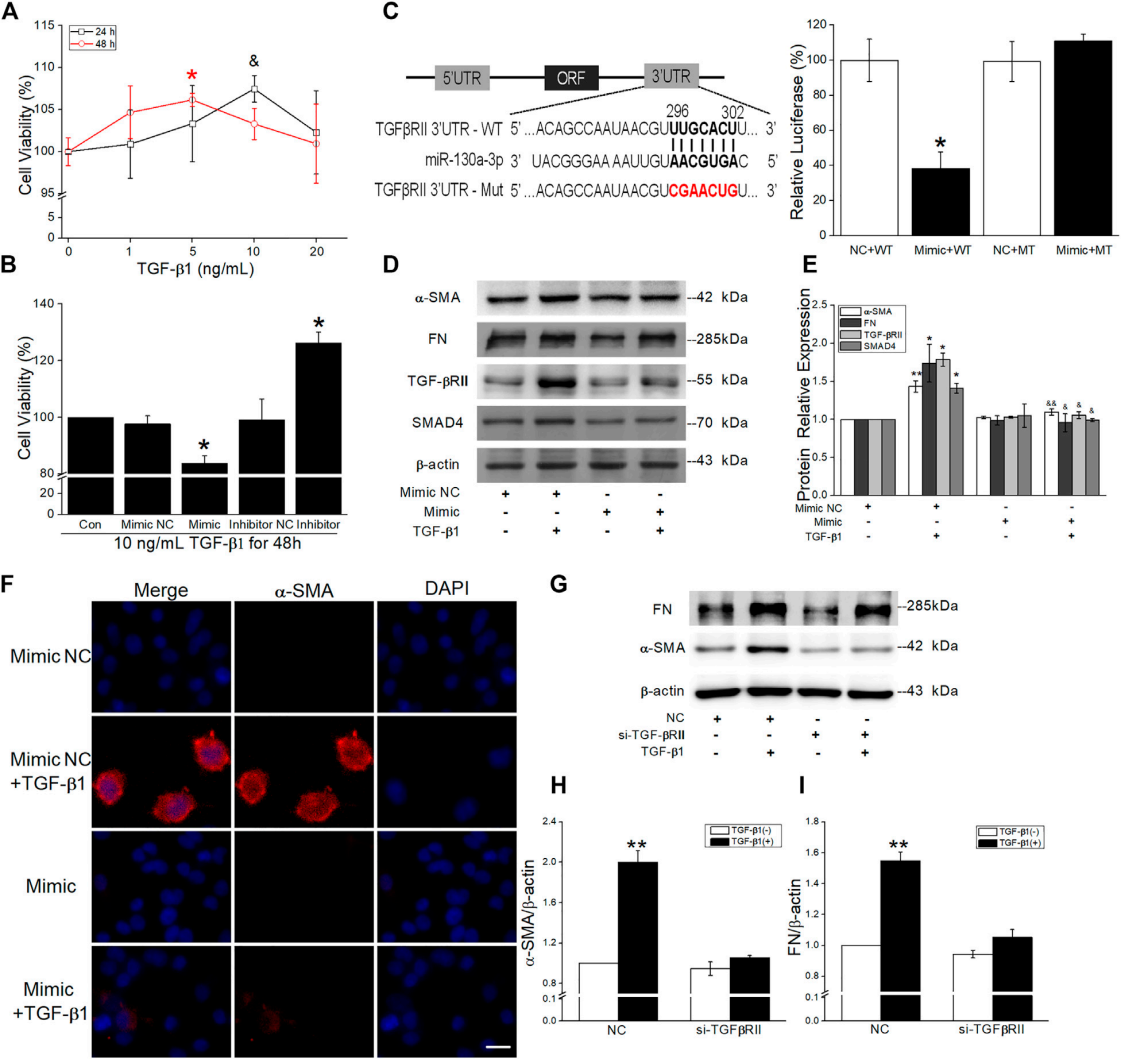
Effect of miR-130a-3p on the differentiation in TGF-β1-induced MRC-5. **(A-B)** CCK-8 assay was used to detect cell viability of MRC-5 cells under TGF-β1 stimulation, and the data for 0 ng/ml or control group was set to 100%. *p < 0.05, versus 0 ng/ml (48 h) or the control group; ^&^ *p* < 0.05, versus 0 ng/ml (24 h), by the Mann–Whitney *U* test, *n* = 3–5. **(C)** Predicted binding sites of miR-130a-3p with TGF-βRII-3′UTR (left). Dual luciferase reporter assays of miR-130a-3p and TGF-βRII. *p < 0.05, versus the NC + WT group, one-way ANOVA followed by Bonferroni’s test, *n* = 3–4. **(D-E)** Representative and statistical data of α-SMA, FN, TGF-βRII, and SMAD4 proteins in MRC-5 after transfection of miR-130a-3p mimic. *p < 0.05, **p < 0.01, versus the Mimic NC group; ^&^ *p* < 0.05, ^&&^ *p* < 0.01, versus the Mimic NC + TGF group, by the Mann–Whitney *U* test, n = 4–6. **(F)** Immunofluorescence staining for α-SMA after miR-130a-3p mimic administration. α-SMA-positive (red) and DAPI (blue). Scale bar = 50 μm. **(G-I)** Representative and statistical data of α-SMA and FN proteins in MRC-5 after transfection of small interfering RNA for TGF-βRII (si-TGF-βRII). **p < 0.01, versus NC group, by the Mann–Whitney *U* test, *n* = 4-5.

The growing evidence demonstrates that both proliferation and differentiation of fibroblasts contribute to the fibrosis formation, and TGF-β1 significantly promoted the viability in MRC-5 at 10 ng/ml for 24 h or 5 ng/ml for 48 h (Figure 9A). To confirm the effect of miR-130a-3p, we transfected MRC-5 with the miR-130a-3p mimic/inhibitor and then disposed with TGF-β1. The CCK-8 assay results showed that the viability was inhibited by miR-130a-3p mimic whereas enhanced by miR-130a-3p inhibitor (Figure 9B). Meanwhile, qRT-PCR and fluorescence assay with CY3-labeled miR-130a-3p were performed to observe transfection efficiency (Supplementary Figure S3).

Target genes of miR-130a-3p were predicted using bioinformatic databases, and the dual luciferase target gene assay proved that miR-130a-3p could directly bind to TGF-βRII (Figure 9C). Meanwhile, Western blot analysis displayed that miR-130a-3p mimic attenuated TGF-β1-induced fibrosis-related protein expression, whereas inhibition of miR-130a-3p enhanced corresponding protein expression in MRC-5 (Figures 9D,E, Supplementary Figure S4). Consistently, miR-130a-3p significantly reduced the immunostaining of TGF-β1-induced α-SMA-positive stress fibers whereas abrogated by miR-130a-3p inhibitor (Figure 9F, Supplementary Figure S5).

In addition, to explore whether miR-130a-3p suppresses the differentiation of MRC-5 by targeting TGF-βRII, we investigated the effect of silencing TGF-βRII on the differentiation of MRC-5. As expected, siTGF-βRII could inhibit the protein expression of fibrotic markers (Figures 9G–I). Taken together, these results indicate that miR-130a-3p plays a vital role in the modulation of fibroblast differentiation, at least partially by targeting TGF-βRII.

## Discussion

PF is a progressive and devastating pulmonary parenchymal disease with a poor prognosis and few curative therapies. The pathogenesis of PF is not definitively identified but thought to be involved with excessive inflammation, oxidative stress, and chronic or repetitive microinjuries of the alveolar epithelium as triggers of the disease (Meyer 2017; Sgalla et al., 2018). Currently, the BLM is the most widely used drug to induce the PF experimental model for developing candidate therapies because the histopathological changes of the lung are similar to those of human PF (Moeller et al., 2008). In this study, we paid attention to the BLM-induced inflammatory and fibrotic phases of PF to evaluate the anti-inflammatory and anti-fibrotic effects of miR-130a-3p.

The development of PF is orchestrated by various cell types that drive a continuous wound-healing response leading to ECM deposition, resulting in eventual loss of function. The upregulated and downregulated DEGs in different cells were subjected to bioinformatics analysis. KEGG pathway analysis for macrophages showed a high enrichment for the PPAR and lysosome signaling pathway, which promotes the inactivation of NF-κB during the inflammatory reaction (Zhu et al., 2016; Korbecki et al., 2019). As for fibroblasts, enrichment for ECM–receptor interaction and ribosome was shown. Of note, the ribosomes and ECM–receptor interaction are closely associated with the progression of PF (Prakash et al., 2019; Hou et al., 2021).

During the pathophysiologic progression of the PF mouse model, the content of inflammatory cytokines increased at seventh day after BLM treatment (Papiris et al., 2018). In addition, the gradual subsidence of inflammatory response along with an increase in fibroproliferation appeared 7–14 days post BLM (Wei et al., 2019; Lv et al., 2020; Ruan and Lv 2020). In addition, we observed that the NF-κB and TGF-β1/Smad signaling cascades were activated, which mediated inflammatory factor release and ECM deposition, respectively.

Many miRNAs are involved in pulmonary diseases, among which miR-130a-3p has been shown to be strongly implicated in pulmonary inflammation (Su et al., 2015; Snyder-Talkington et al., 2016; Parzibut et al., 2021), regeneration, and remodeling of the injured respiratory system (Yu et al., 2019; Shi et al., 2020). However, the detailed mechanism of miR-130a-3p in BLM-induced PF is still lacking (Su et al., 2015; Chen et al., 2018). Following exposure of mice to BLM, a significant increase of miR-130a-3p was observed at day 7 and then declined significantly at day 28 post exposure. Simultaneously, we discovered that miR-130a-3p markedly reduced the number of inflammatory cells, which produced inflammatory cytokines such as IL-1β, IL-6, and TNF-α in BALF (Zhang et al., 1993; Papiris et al., 2018), confirmed by histopathological examination in the inflammatory phase. Tissue damage and inflammation participate in repairing and reconstructing the lung, which are important triggers for fibrosis (Mack 2018). As expected, lung histopathological examination and HYP content demonstrated that BLM caused severe lung inflammation, injury, and fibrosis in the fibrotic phase. MiR-130a-3p observably normalized the alveolar architecture and decreased the area of fibrosis, which were shown in histopathological sections. Moreover, miR-130a-3p could also downregulate HYP content, which indicated that collagen secretion was reduced and ECM deposition was inhibited.

In LPS-induced inflammatory injury, the activated NF-κB signaling pathway would drive numerous inflammation-related gene expressions, including the potent inflammation factor TNF-α, which could conversely activate the canonical and noncanonical NF-κB pathway through TNFR1 and TNFR2 (Borghi et al., 2016). Meanwhile, NF-κB signaling pathway activation may increase the level of miR-130a-3p by binding to the P65 promoter element, which showed a suppression effect on the NF-κB signaling pathway by inhibiting the expression of TNF-α (Chan et al., 2005; Zhou et al., 2010). Among the various cytokines participating in the process of the PF mouse model (Barkauskas and Noble 2014), TGF-β1 is currently the most potent profibrogenic cytokines, which can lead to EMT, provoke fibroblast proliferation, promote myofibroblast formation, and result in ECM deposition (Kim et al., 2018; Lodyga and Hinz 2020). In our study, we found that miR-130a-3p could restrain the TGF-β1/Smad signaling pathway in the fibrotic phase of the PF mouse model.

**FIGURE 10:**
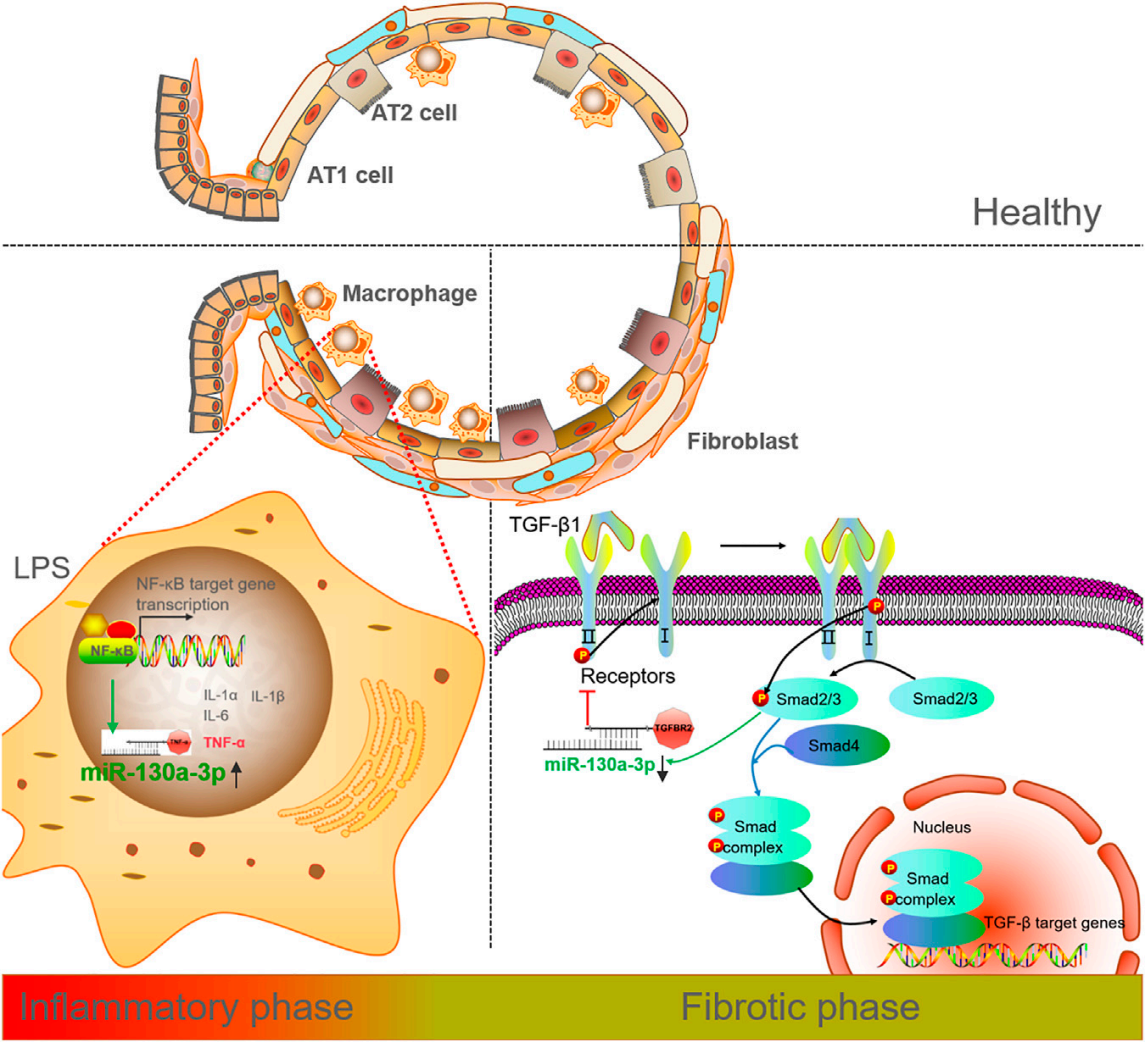
Diagram of miR-130a-3p on PF in inflammatory and fibrotic phases. In the inflammatory phase, activation of the NF-κB signaling pathway increases the expression of miR-130a-3p, while overexpression of miR-130a-3p inhibits LPS-induced production of TNF-α. In the fibrotic phase, TGF-β1 decreases miR-130a-3p expression through the TGF-β1/Smad pathway. Meanwhile, miR-130a-3p targets TGF-βRII and therefore inhibits the TGF-β1/Smad pathway.

To identify the miR-130a-3p target genes responsible for these effects, we used bioinformatics and functional knowledge associated with NF-κB and TGF-β/Smad and chose TNF-α and TGF-βRII as candidate genes for further *in vitro* study. TNF-α, as a central core in the cytokine network, may regulate inflammation in the innate immunity (Bradley 2008; Zelová and Hošek 2013). Subsequently, we observed that miR-130a-3p inhibited fibroblast proliferation and reduced ECM deposition. The downregulation of miR-130a-3p by TGF-β1 was time- and concentration-dependent, which could be attenuated by both TGF-βRII and Smad3 inhibitors. Moreover, overexpression of miR-130a-3p restrained TGF-β1-induced α-SMA, which was the marker of the cell differentiation phenotype. Meanwhile, according to the result of dual luciferase target gene assay and evidence for TGF-βRII downregulation after miR-130a-3p administration, it was testified that miR-130a-3p desensitized TGF-β1 signaling pathways by directly targeting TGF-βRII to downregulate profibrotic genes (Figure 10). Considering that the NF-κB and TGF-β1/Smad signaling cascades were activated during inflammatory and fibrotic phases, respectively, the therapeutic strategy targeting these two pathways may provide a promising effect on PF treatment.

## Conclusion

MiR-130a-3p exerts an anti-inflammatory and anti-fibrotic effect in BLM-induced PF by suppressing the proinflammatory factor TNF-α and profibrogenic activity of TGF-β1 signaling, implying an underlying therapeutic agent in the therapy of PF patients.

## Data Availability

The datasets presented in this study can be found in online repositories. The names of the repository/repositories and accession number(s) can be found below: Gene Expression Omnibus with accession code GSE141259.
