# Supplementary material for: MiR-130a-3p Alleviates Inflammatory and Fibrotic Phases of Pulmonary Fibrosis Through Proinflammatory Factor TNF-α and Profibrogenic Receptor TGF-βRII
Source: Front Pharmacol. 2022 Mar 30;13:863646. doi: 10.3389/fphar.2022.863646 (PMC9006815; doi:10.3389/fphar.2022.863646)
Supplement: Supplementary file 1 [file DataSheet1.ZIP › Supplementary materials/Supplementary_Material.docx]

*Supplementary Material*

MiR-130a-3p Alleviates Inflammatory and Fibrotic Phases of Pulmonary Fibrosis through Proinflammatory Factor TNF-α and Profibrogenic Receptor TGF-βII

Yan Ding ^1^, Yapeng Hou ^1^, Yanhong Liu ^1^, Tong Yu ^1^, Yong Cui ^2^, Hongguang Nie ^1*^

^1^Department of Stem Cells and Regenerative Medicine, College of Basic Medical Science, China Medical University, Shenyang, 110122, China

^2^Department of Anesthesiology, the First Hospital of China Medical University, Shenyang, 110001, China

*** Correspondence:**Hongguang Nie

[hgnie@cmu.edu.cn](mailto:hgnie@cmu.edu.cn)

**Supplementary Table 1.**

Primer sequences of qRT-PCR

| Gene | Forward primer sequence | Reverse primer sequence |
| --- | --- | --- |
| TGF-β1  α-SMA  FN  TNF-α  IL1β  IL6  P50  IκBα  CD14  CD19  CD68  TGF-βRⅠ  TGF-βRⅡ  SMAD2  SMAD3  Col 1  Col 3  E-cadherin  GAPDH  miR-130a-3p  U6 | 5’-CCA CCT GCA AGA CCA TCG AC-3’  5’-CTA TGA AGG CTA TGC CCT GCC-3’  5’-CGA GGT GAC AGA GAC CAC AA-3’  5’-CTA TGG CCC AGA CCC TCA CA-3’  5’-CAG GCA GGC AGT ATC ACT CA-3’  5’-TGT GCA ATG GCA ATT CTG AT-3’  5’-AAA TGG GAA ACC GTA TGA-3’  5’-ACC AAC CAG CCA GGA ATT-3’  5’-TCT TGA ACC TCC GCA ACG-3’  5’-TGG TGG AGG TAG AAG AGG G-3’  5’-ATC TTG CTA GGA CCG CTT AT-3’  5’-AAA CTT GCT CTG TCC ACG G-3’  5’-GTA ATA GGA CTG CCC ATC CAC-3’  5’-AGC AGA ATA CCG AAG GCA G-3’  5’-GCT GCT CTC CAA TGT CAA CAG-3’  5’-TTG TGC GAT GAC GTG ATC TGT-3’  5’-TGG AAA CTG GGG AAA CAT GC-3’  5’-CAG GTC TCC TCA TGG CTT TGC-3’  5’-UUC UCC GAA CGU GUC ACG UTT-3’  5’-CAG UGC AAU GUU AAA AGG GCA U-3’  5’-GGA ACG ATA CAG AGA AGA TTA GC-3’ | 5’-CTG GCG AGC CTT AGT TTG GAC-3’  5’-GCT TCT CCT TGA TGT CTC GCA C-3’  5’-CTG GAG TCA AGC CAG ACA CA-3’  5’-TTG AGA TCC ATG CCG TTG G-3’  5’-AGG TGC TCA TGT CCT CAT CC-3’  5’-TCC AGT TTG GTA GCA TCC ATC-3’  5’-TAG CCT CGT GTC TTC TGT C-3’  5’-CAC AGG CAA GAT GTA GAG GG-3’  5’-TGG GCA ATA CTC AGT ACC TTG A-3’  5’-GGA AGG GTG TTG ACT GGT TA-3’  5’-GTG GCT GTA GGT GTC ATC GT-3’  5’-AAT GGC TGG CTT TCC TTG-3’  5’-GAT TTC TGG TTG TCA CAG GTG-3’  5’-TTT GTC CAA CCA CTG TAG AGG T-3’  5’-TCT TCC GAT GTG TCT CCG T -3’  5’-TTG GTC GGT GGG TGA CTC TG-3’  5’-GGA TTG CCG TAG CTA AAC TGA-3’  5’-CTT CCG AAA AGA AGG CTG TCC-3’  5’-ACG UGA CAC GUU CGG AGA ATT-3’  5’-GCC CUU UUA ACA UUG CAC UGU U-3’  5’-TGG AAC GCT TCA CGA ATT TGC G-3’ |

**
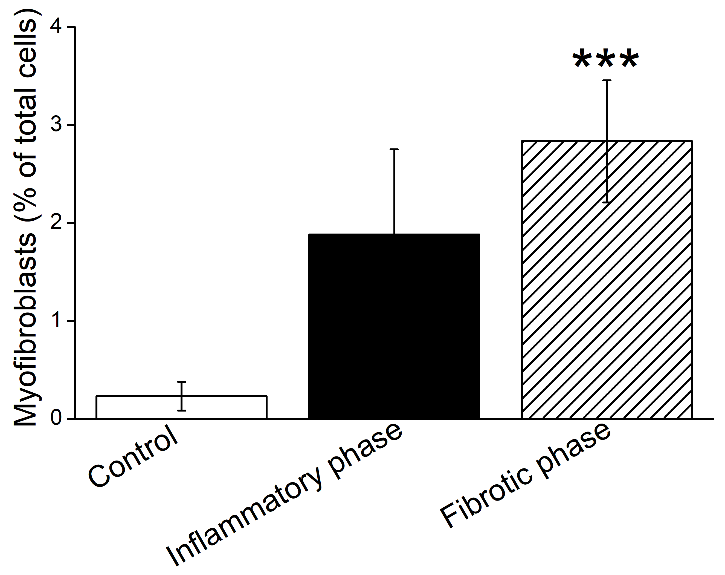
**

**Supplementary Figure 1.** The percentage of myofibroblasts in BLM-induced PF inflammatory and fibrotic phases. ****P* < 0.001, *versus* Control group. Mann-Whitney U test was used to analyze the difference of the means for significance.

**
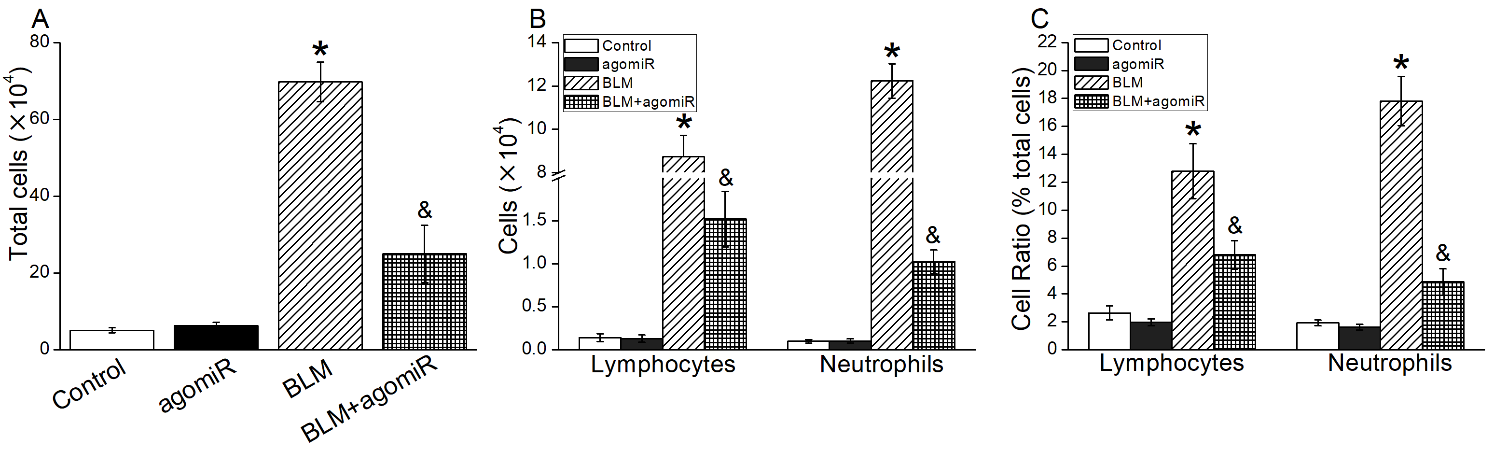
**

**Supplementary Figure 2.** Cell counts and classification in BALF. **(A)** Total cells. **(B)** Cell counts of lymphocytes and neutrophils. **(C)** Cell ratio of lymphocytes and neutrophils (% total cells). **P* < 0.05, *versus* Control group; ^&^*P* < 0.05, *versus* BLM group, *n* = 3-4. Mann-Whitney U test was used to analyze the difference of the means for significance.

**
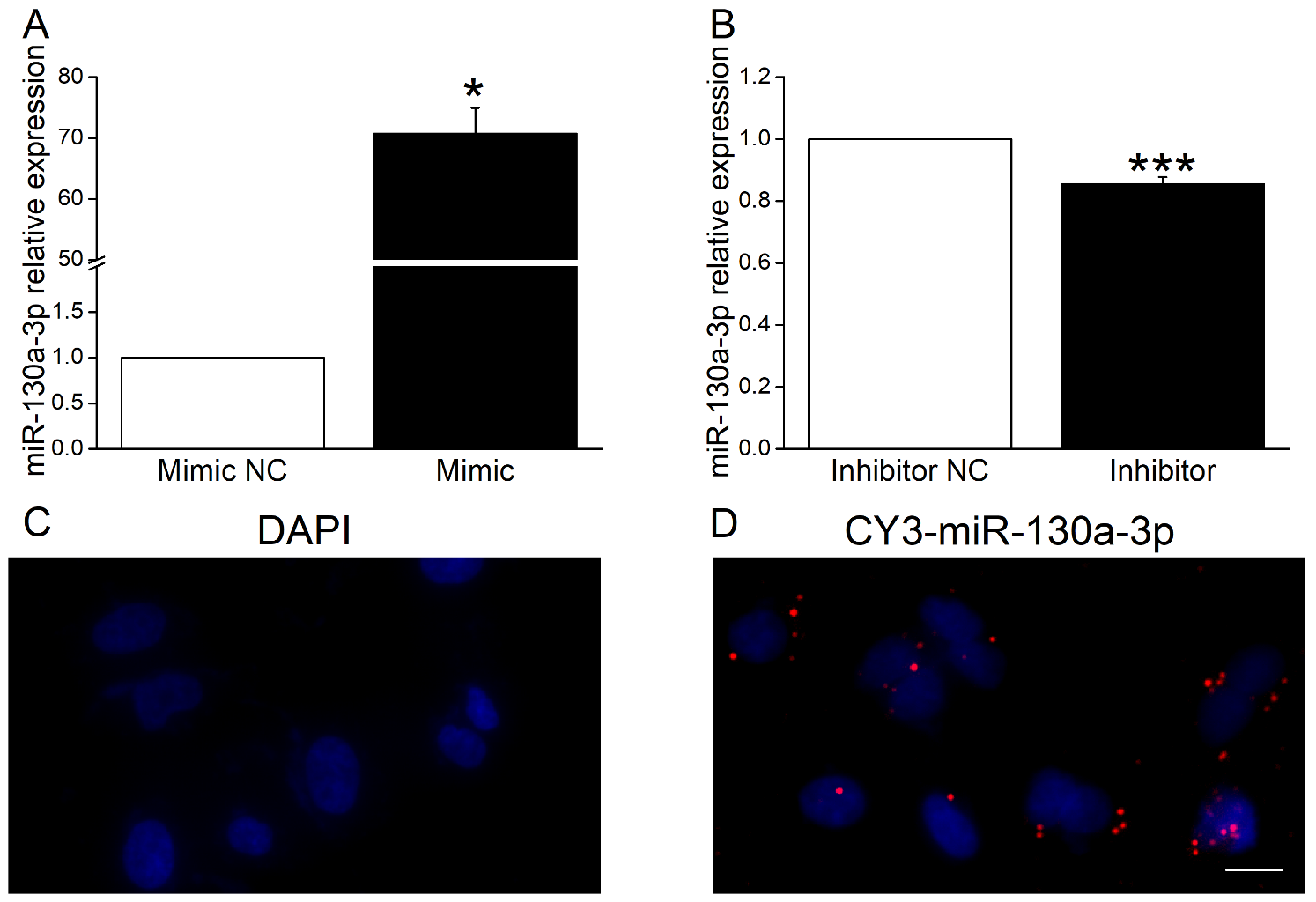
**

**Supplementary Figure 3.** The transfection efficiency of miR-130a-3p in MRC-5 cells. **(A)** MiR-130a-3p mimic (Mimic), negative control (Mimic NC) were transfected in MRC-5 cells, and the transfection efficiency was shown. **P* < 0.05, *versus* Mimic NC group, *n* = 4. Mann-Whitney U test was used to analyze the difference of the means for significance. **(B)** MiR-130a-3p inhibitor (Inhibitor), and inhibitor NC (Inhibitor NC) were transfected in MRC-5 cells, and the transfection efficiency was shown. ****P* < 0.001, *versus* Inhibitor NC group, *n* = 4-5. One-way ANOVA followed by Bonferroni’s test was used to analyze the difference of the means for significance. **(C-D)** MRC-5 cells were transfected with CY3-miR-130a-3p, and cultured for 24 h. A representative CY3-miR-130a-3p and DAPI merged image was seen (red and blue). Scale bar = 10 μm.

**
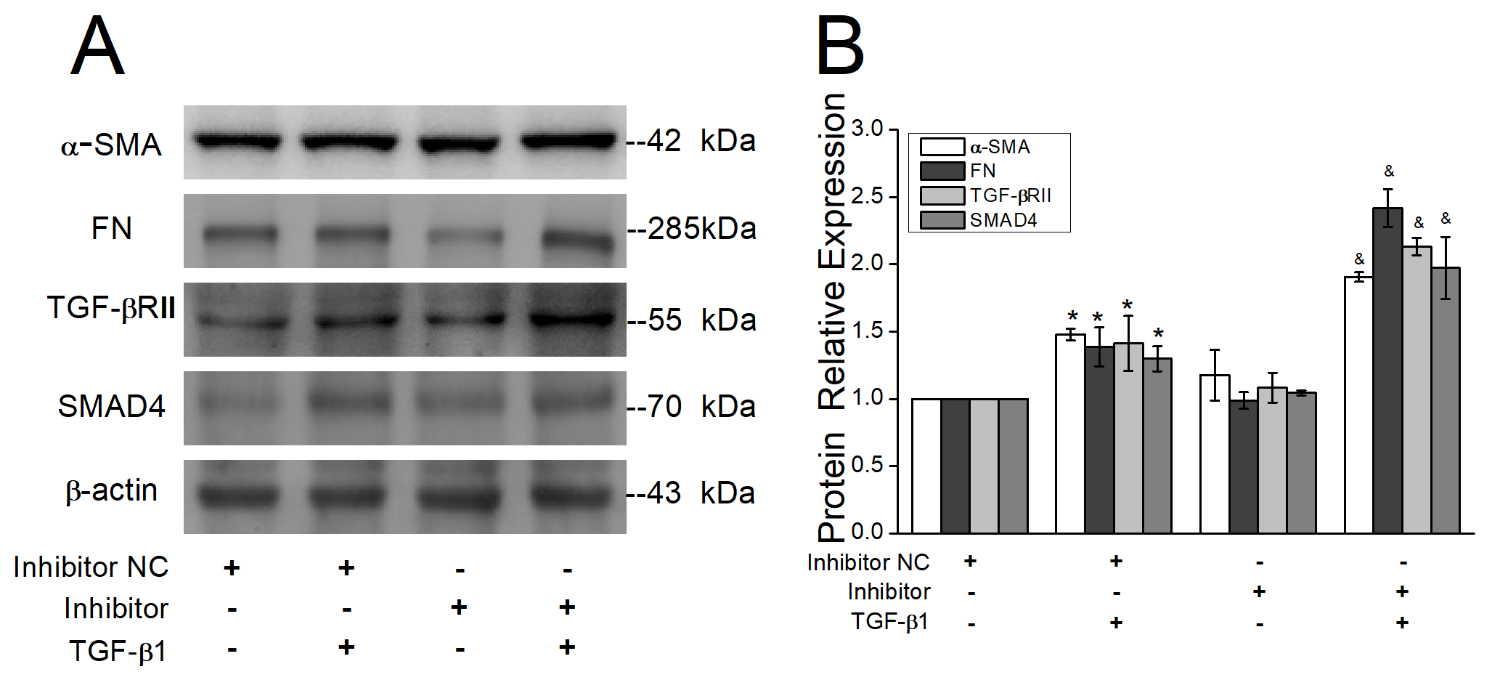
**

**Supplementary Figure 4. (A-B)** Representative and statistical data of α-SMA, FN, TGF-βRII and SMAD4 proteins in MRC-5 after transfection of miR-130a-3p inhibitor. *P < 0.05, *versus* Inhibitor NC group; ^&^P < 0.05, *versus* Inhibitor NC + TGF group, n = 4-6. Mann-Whitney U test was used to analyze the difference of the means for significance.

**
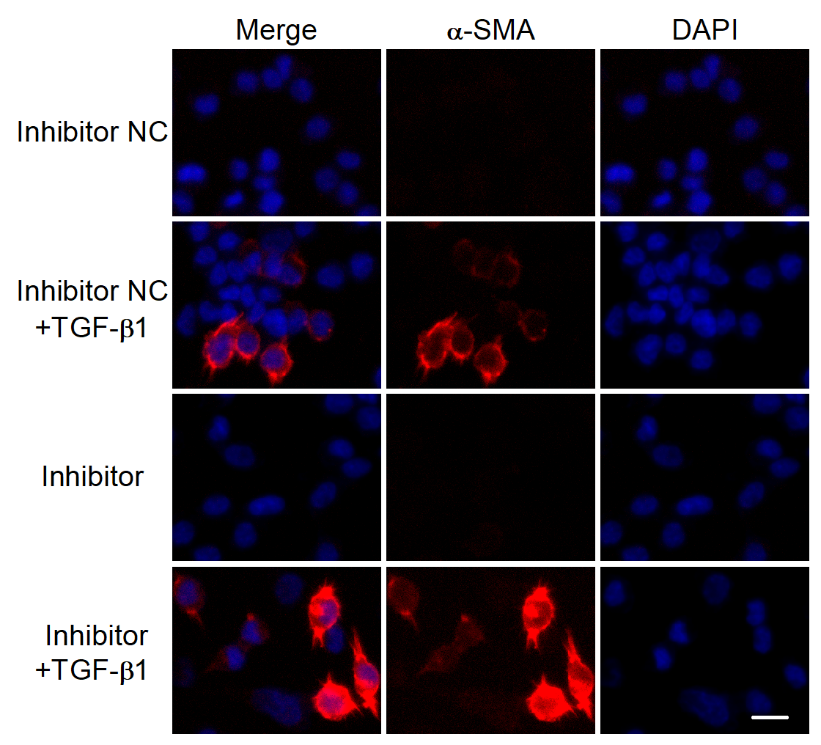
**

**Supplementary Figure 5.** The immunofluorescence staining for α-SMA after miR-130a-3p inhibitor and/or TGF-β1 administration. α-SMA-positive (red) and DAPI (blue). Scale bar = 50 μm.
