# Supplementary figures and images for: MiR-130a-3p Alleviates Inflammatory and Fibrotic Phases of Pulmonary Fibrosis Through Proinflammatory Factor TNF-α and Profibrogenic Receptor TGF-βRII (part 2 of 2)
Source: Front Pharmacol. 2022 Mar 30;13:863646. doi: 10.3389/fphar.2022.863646 (PMC9006815; doi:10.3389/fphar.2022.863646)

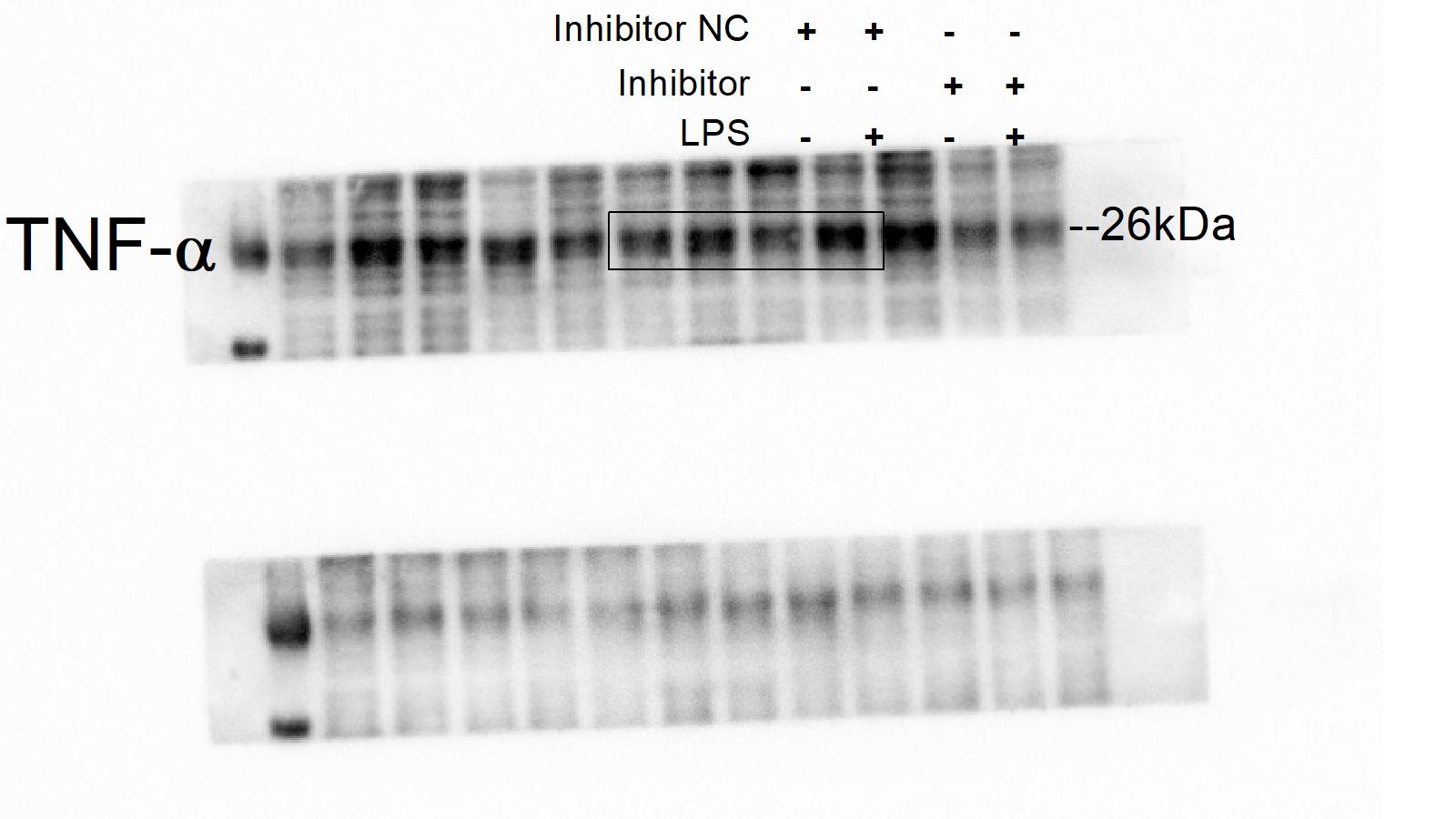

Supplement: Supplementary file 1 [file DataSheet1.ZIP › Supplementary materials/Original source data/uncropped images/Fig.7/Fig.7K TNF-a.tiff]

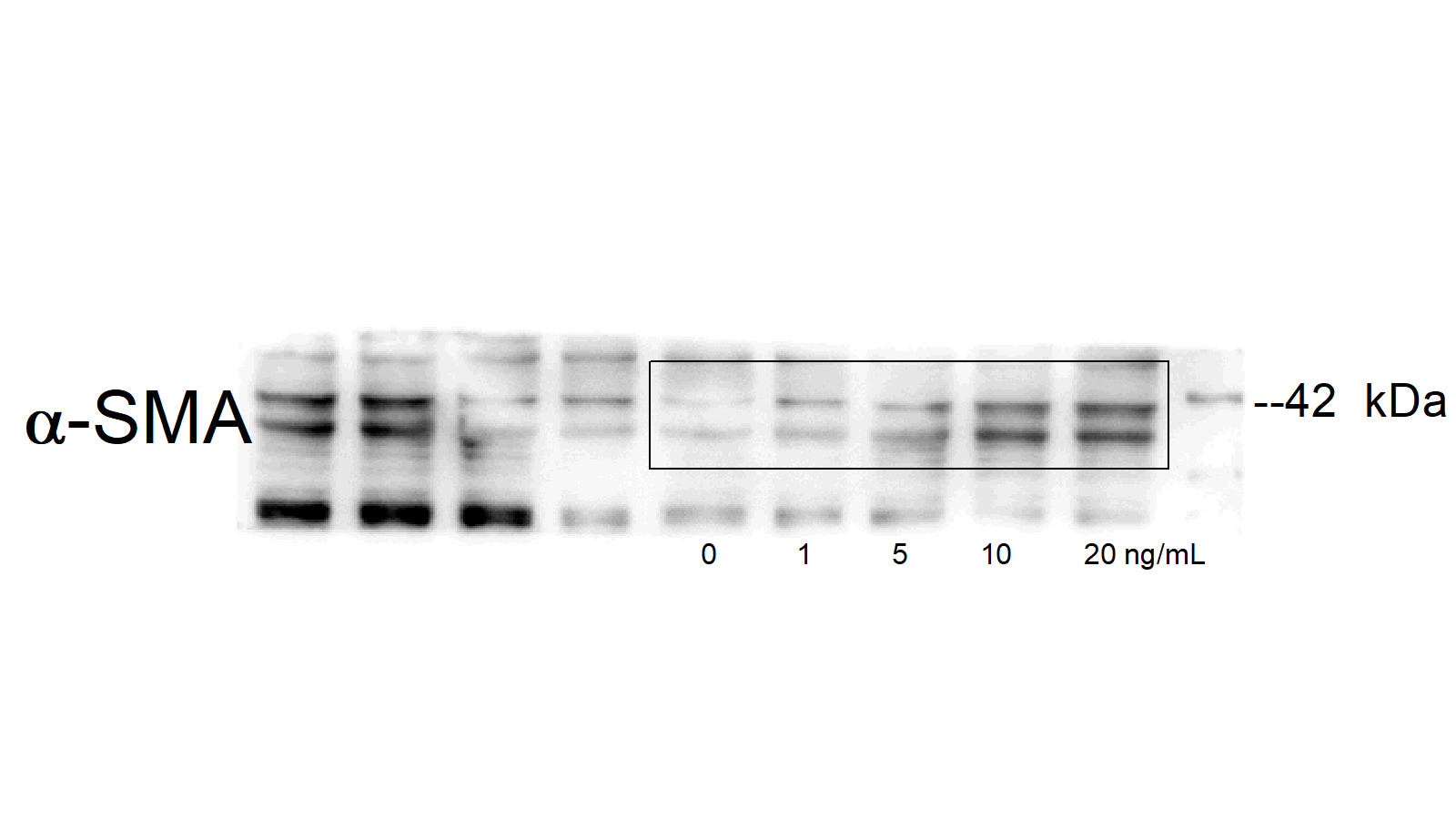

Supplement: Supplementary file 1 [file DataSheet1.ZIP › Supplementary materials/Original source data/uncropped images/Fig.8/Fig.8B a-SMA.tiff]

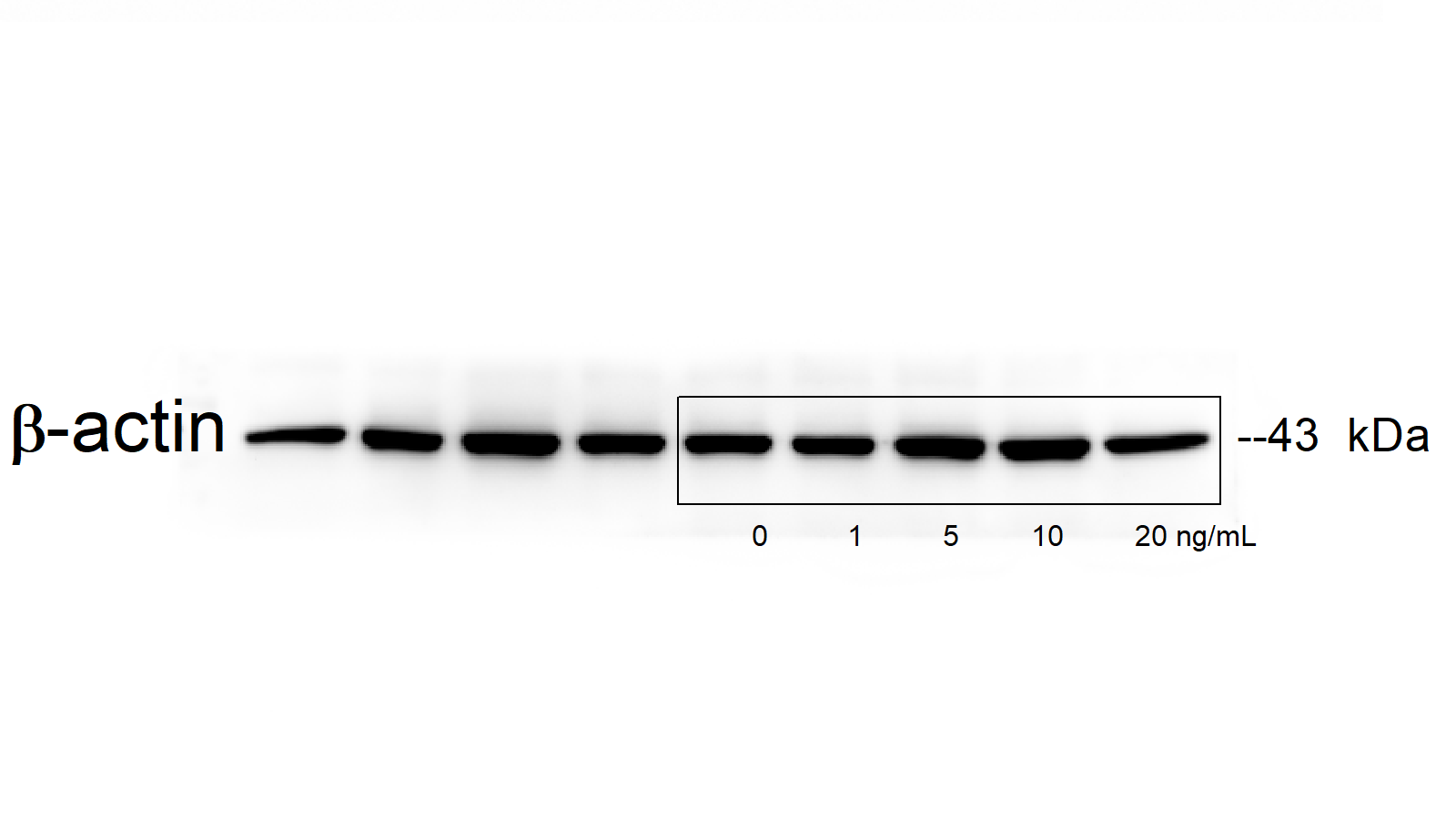

Supplement: Supplementary file 1 [file DataSheet1.ZIP › Supplementary materials/Original source data/uncropped images/Fig.8/Fig.8B b-actin.tiff]

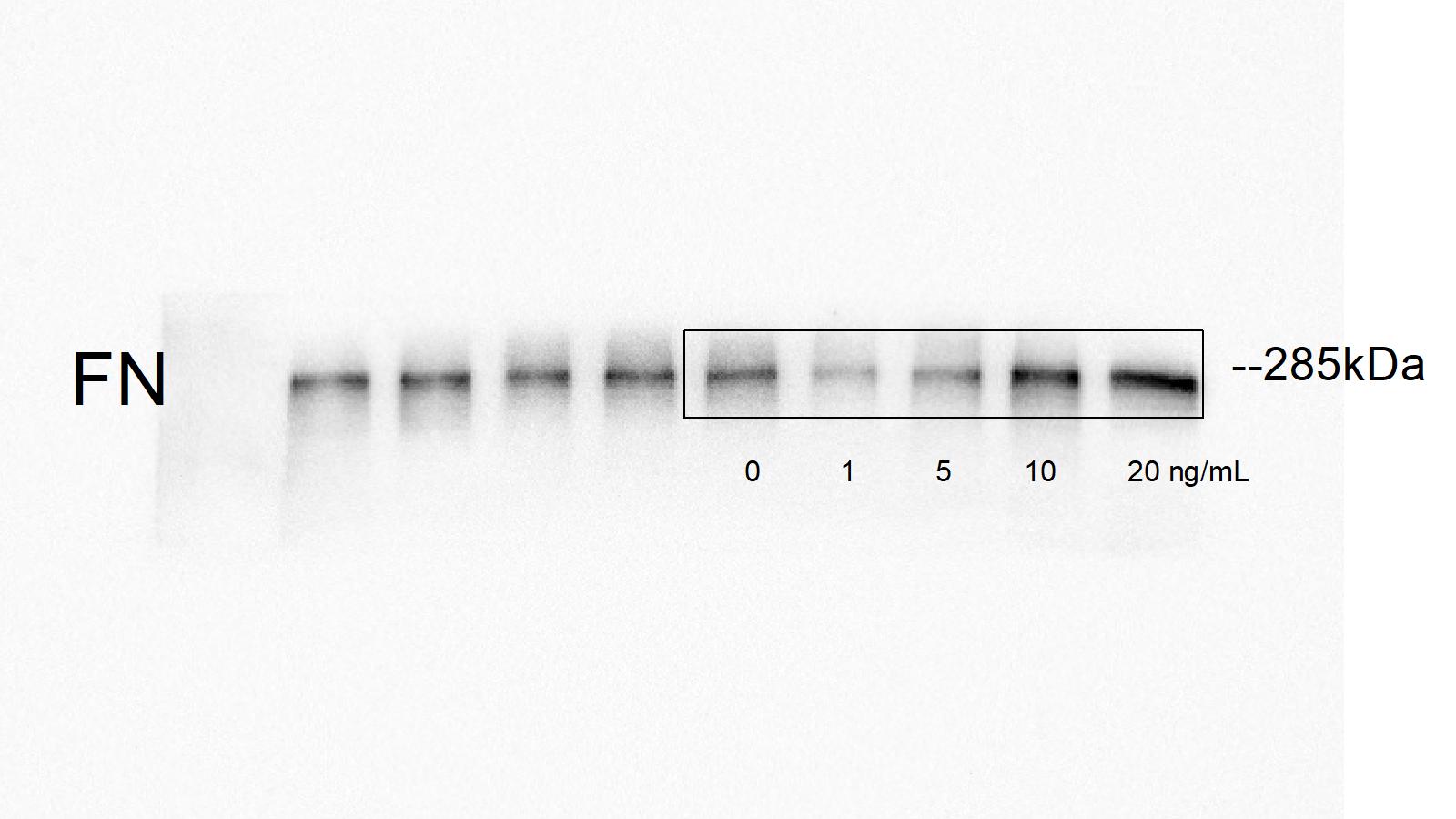

Supplement: Supplementary file 1 [file DataSheet1.ZIP › Supplementary materials/Original source data/uncropped images/Fig.8/Fig.8B FN.tiff]

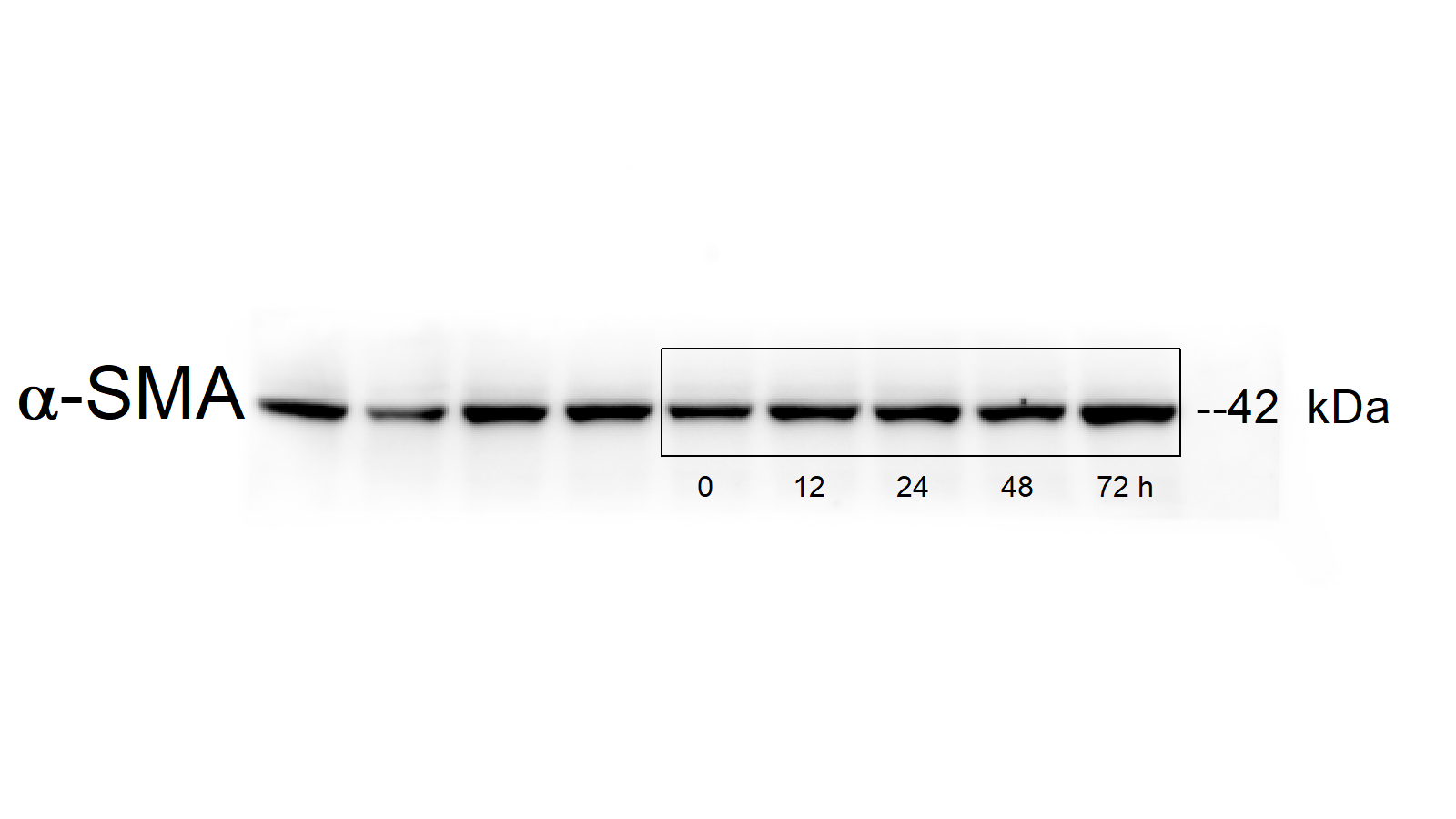

Supplement: Supplementary file 1 [file DataSheet1.ZIP › Supplementary materials/Original source data/uncropped images/Fig.8/Fig.8E a-SMA.tiff]

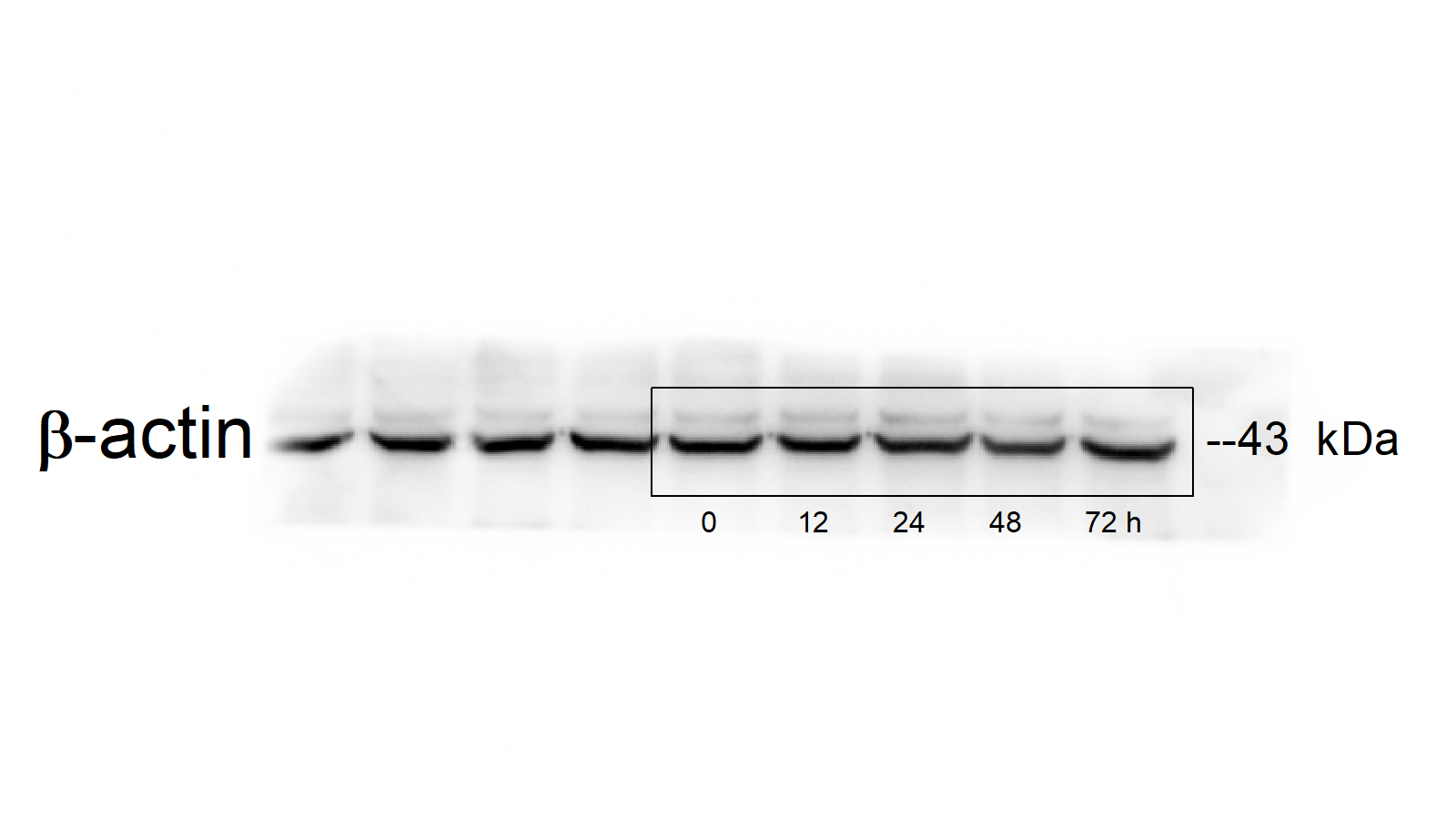

Supplement: Supplementary file 1 [file DataSheet1.ZIP › Supplementary materials/Original source data/uncropped images/Fig.8/Fig.8E b-actin.tiff]

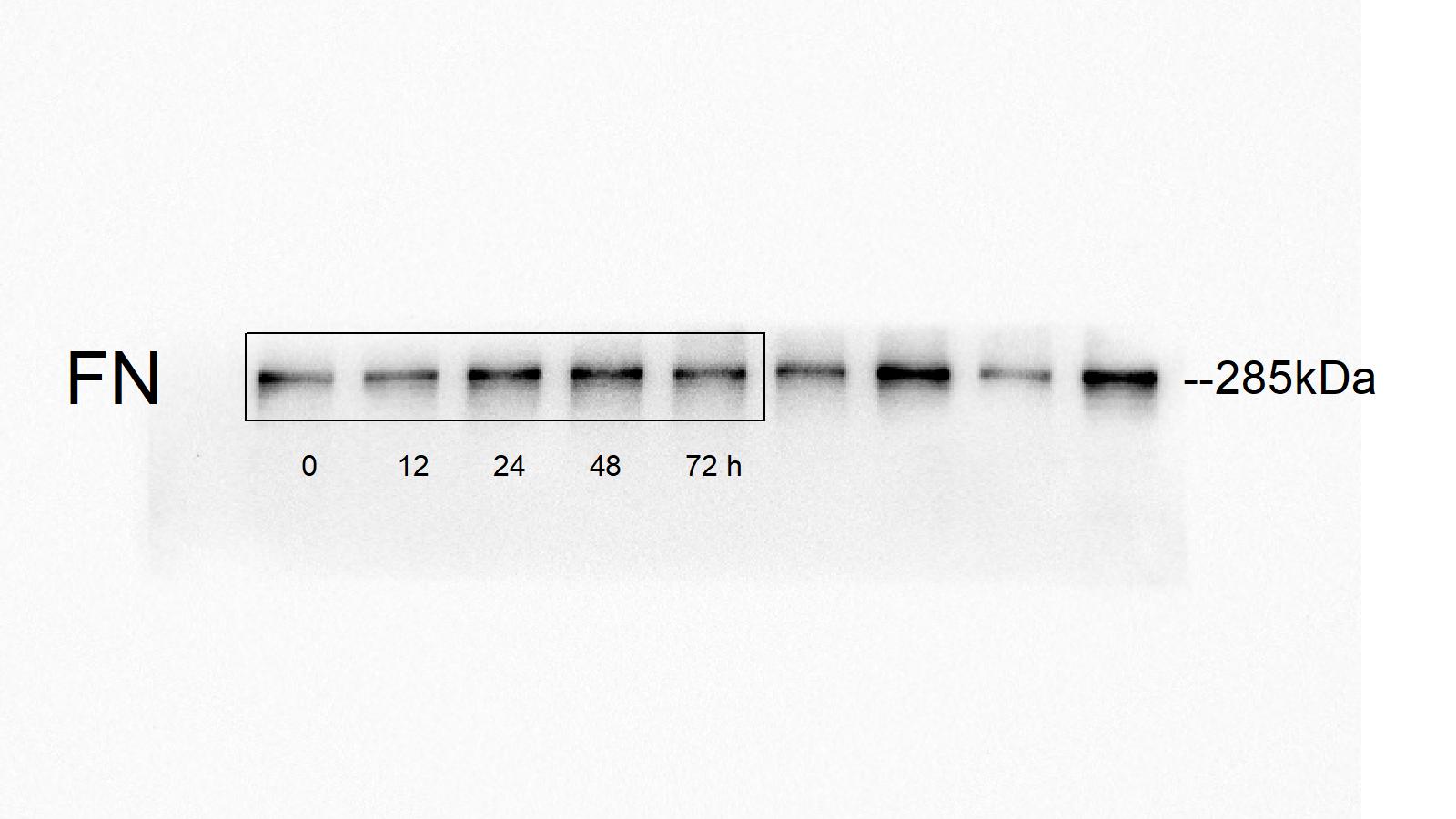

Supplement: Supplementary file 1 [file DataSheet1.ZIP › Supplementary materials/Original source data/uncropped images/Fig.8/Fig.8E FN.tiff]

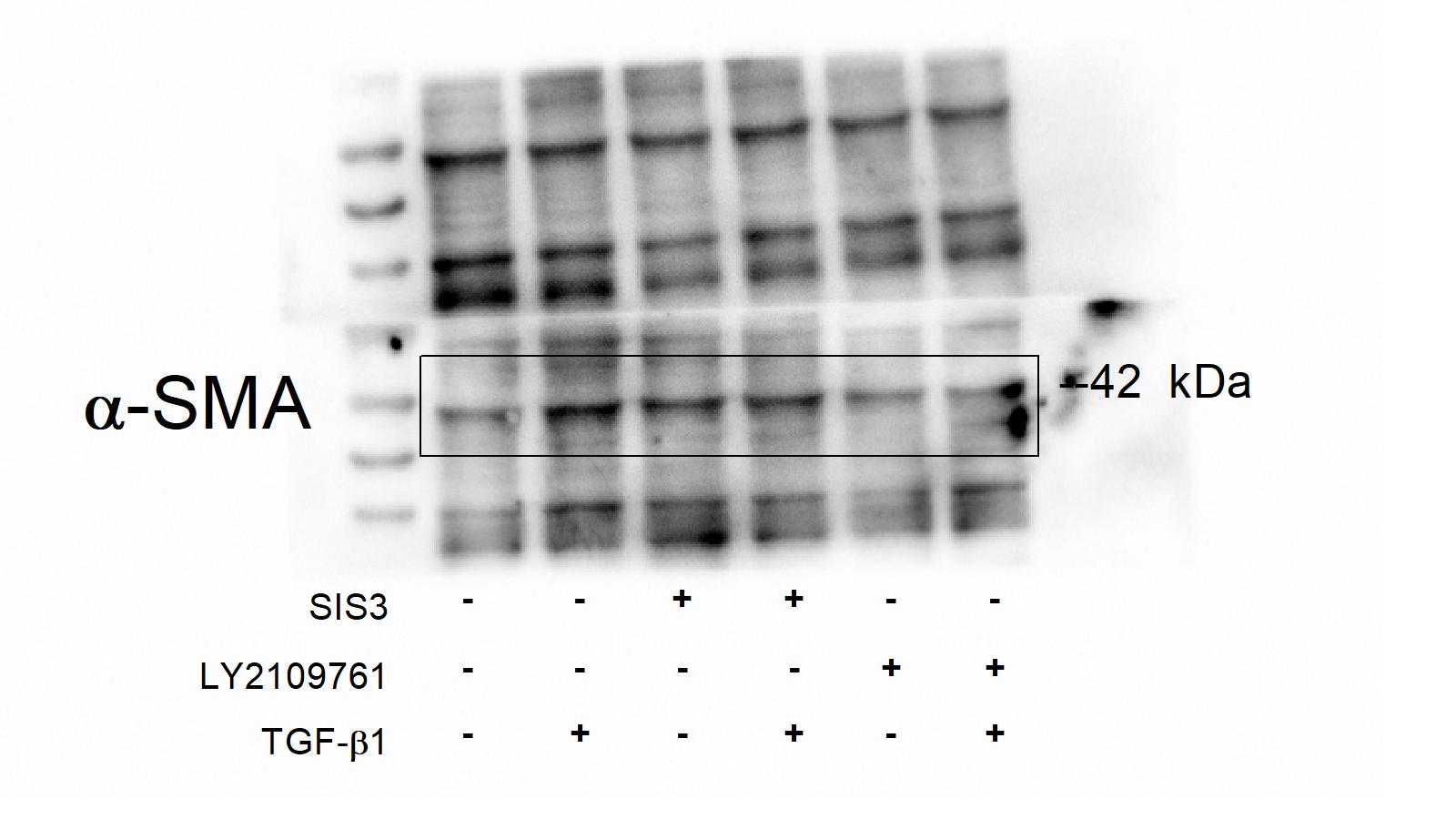

Supplement: Supplementary file 1 [file DataSheet1.ZIP › Supplementary materials/Original source data/uncropped images/Fig.8/Fig.8H a-SMA.tiff]

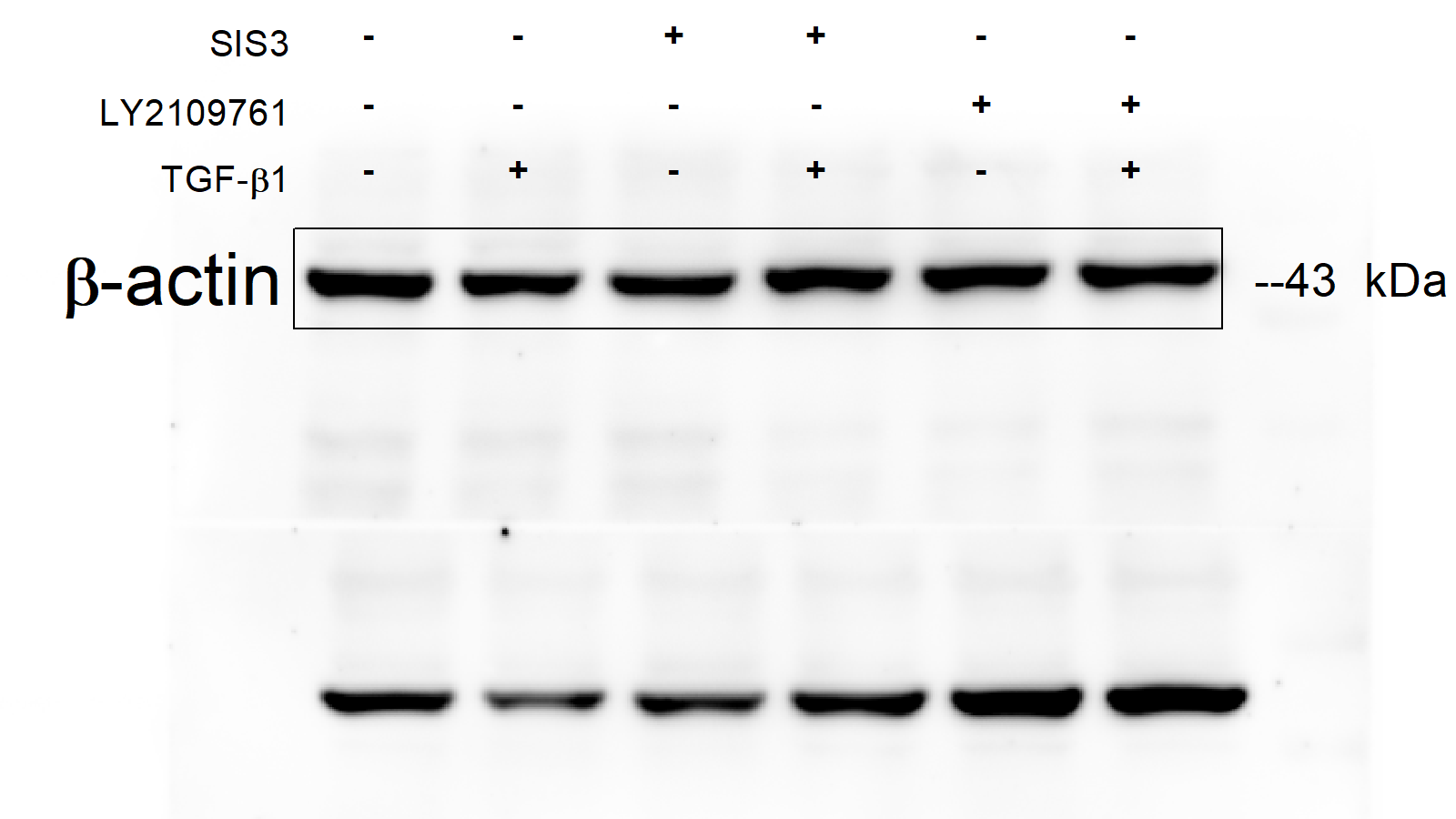

Supplement: Supplementary file 1 [file DataSheet1.ZIP › Supplementary materials/Original source data/uncropped images/Fig.8/Fig.8H b-actin.tiff]

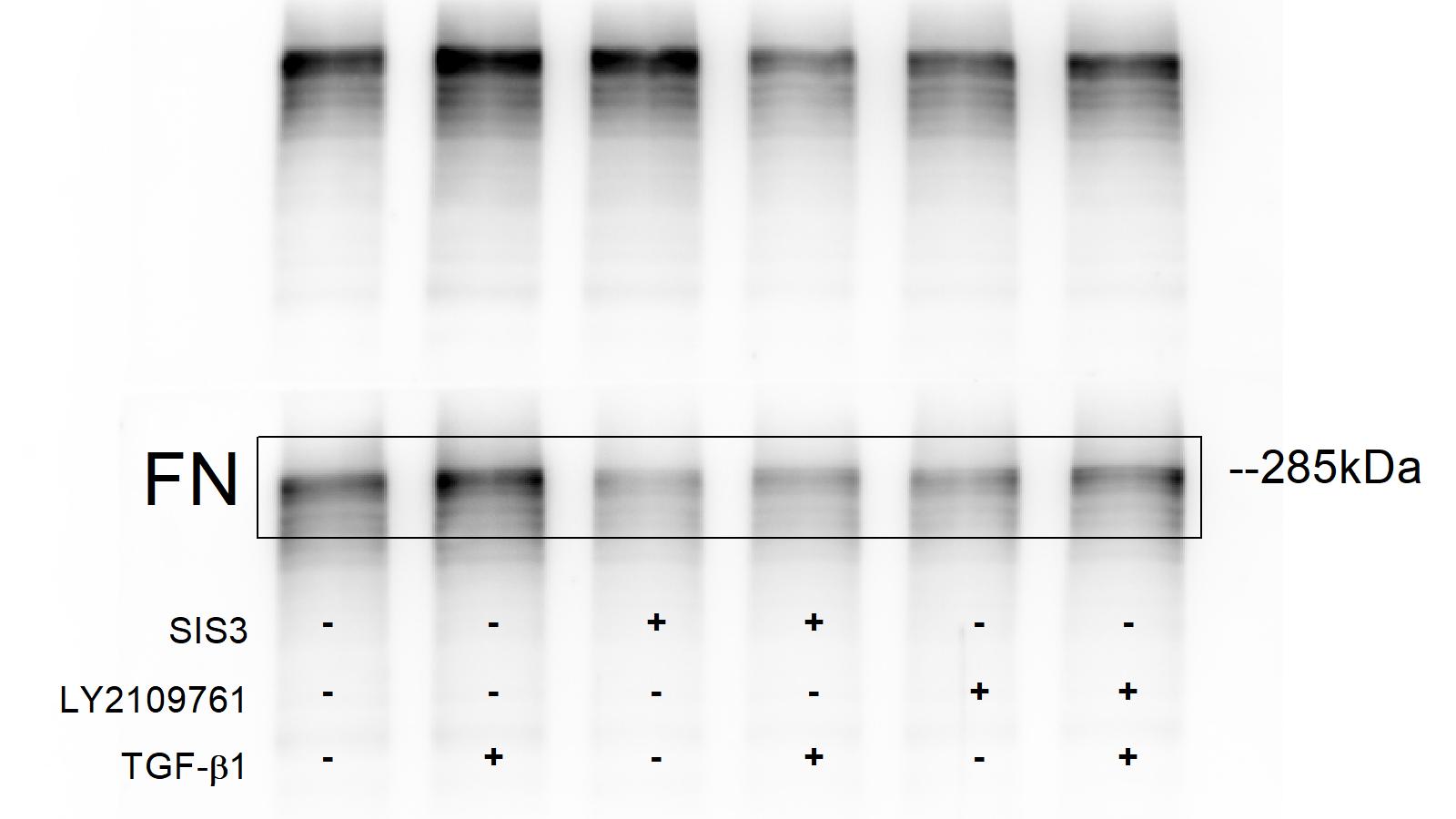

Supplement: Supplementary file 1 [file DataSheet1.ZIP › Supplementary materials/Original source data/uncropped images/Fig.8/Fig.8H FN.tiff]

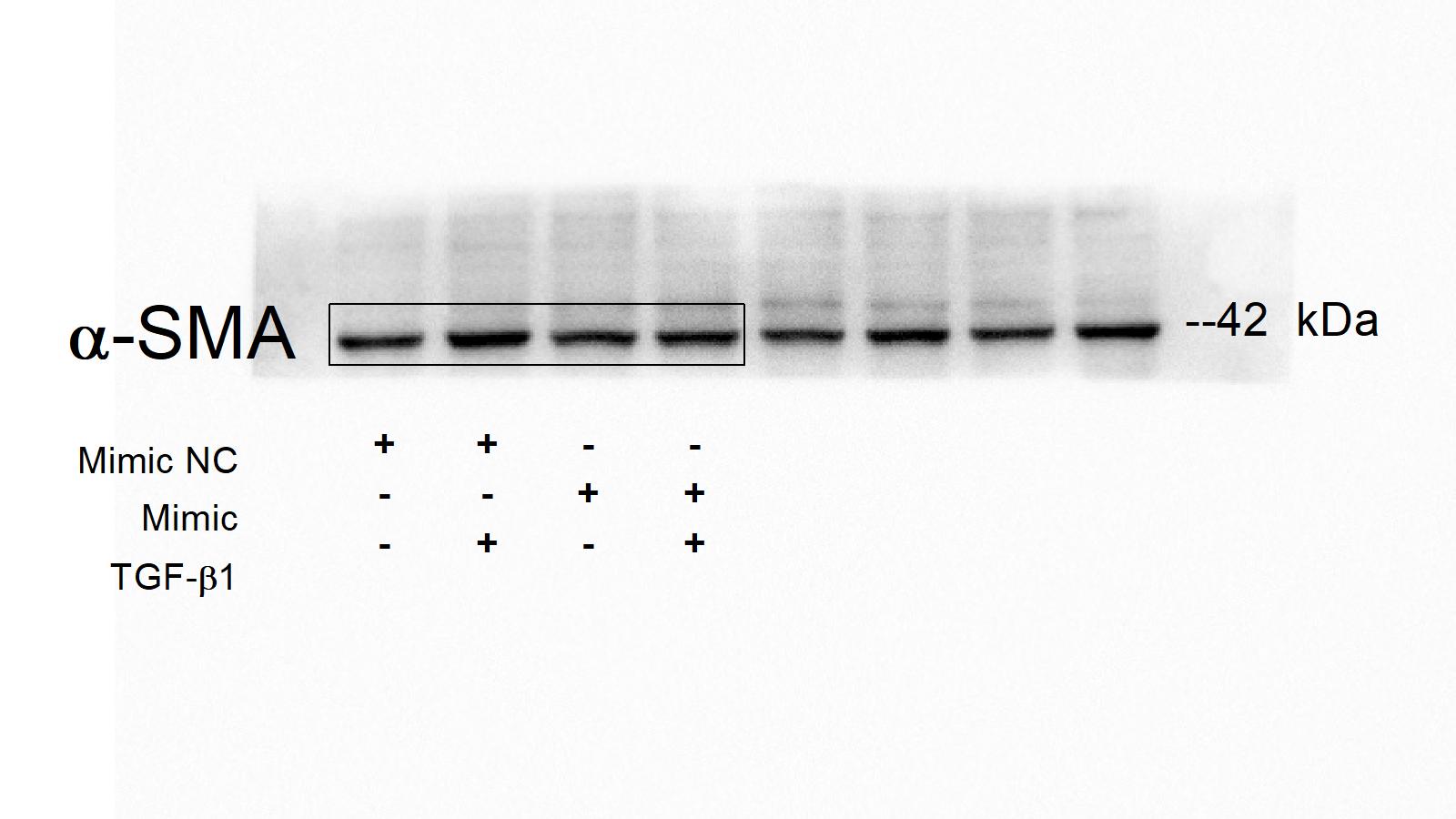

Supplement: Supplementary file 1 [file DataSheet1.ZIP › Supplementary materials/Original source data/uncropped images/Fig.9/Fig.9D a-SMA.tiff]

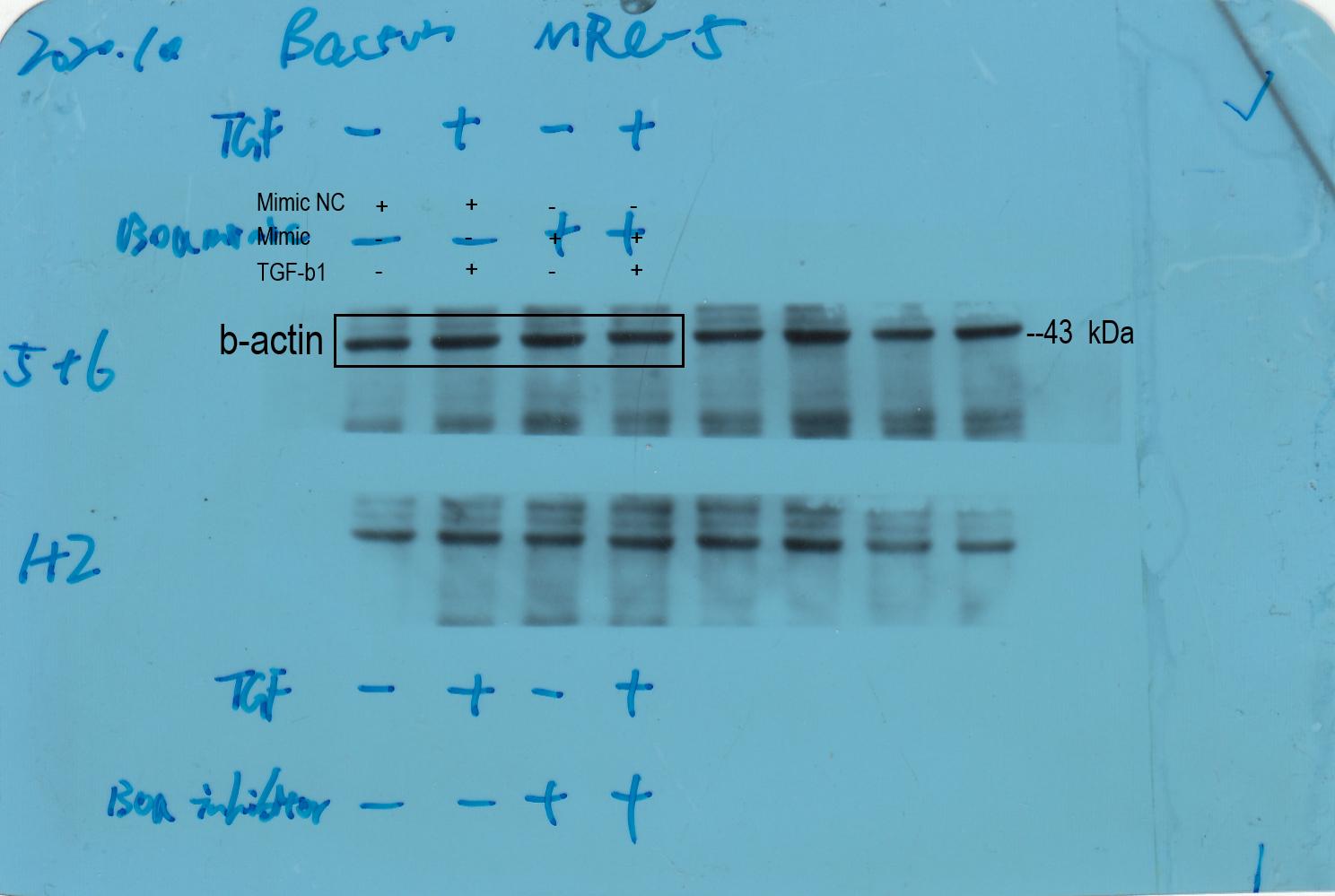

Supplement: Supplementary file 1 [file DataSheet1.ZIP › Supplementary materials/Original source data/uncropped images/Fig.9/Fig.9D b-actin.tif]

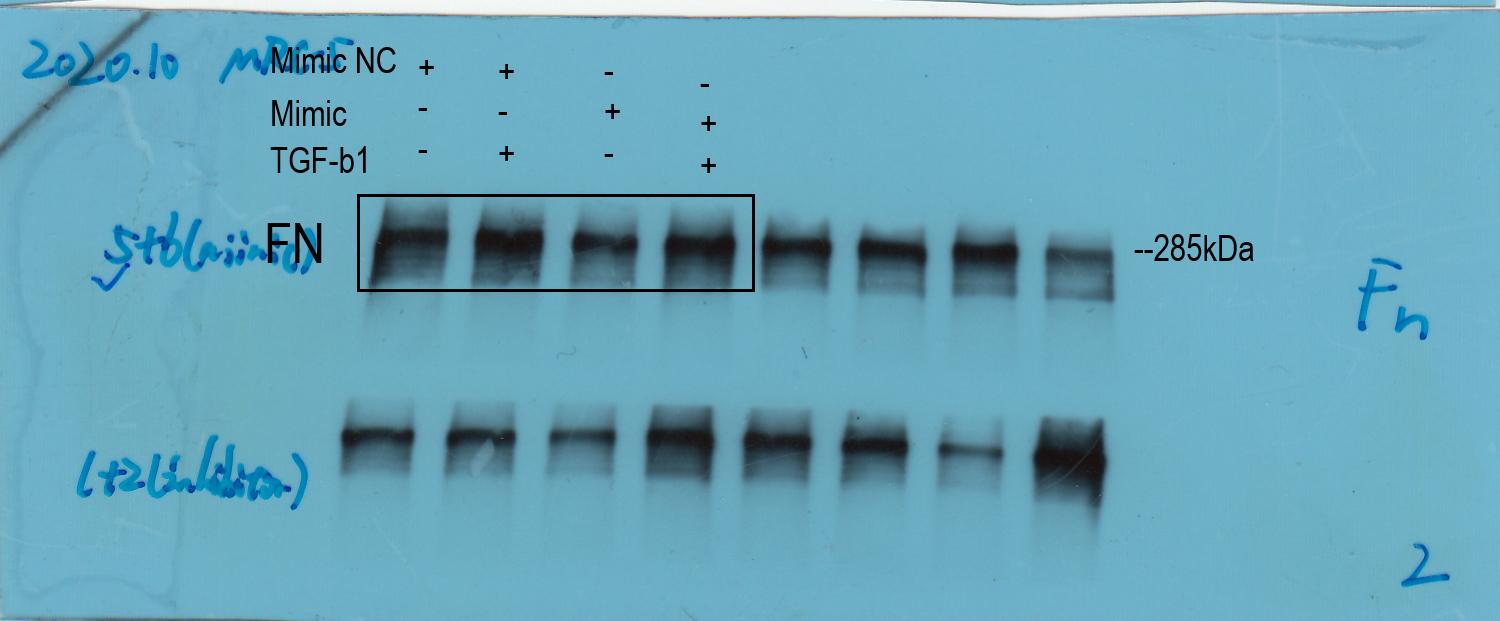

Supplement: Supplementary file 1 [file DataSheet1.ZIP › Supplementary materials/Original source data/uncropped images/Fig.9/Fig.9D FN.tif]

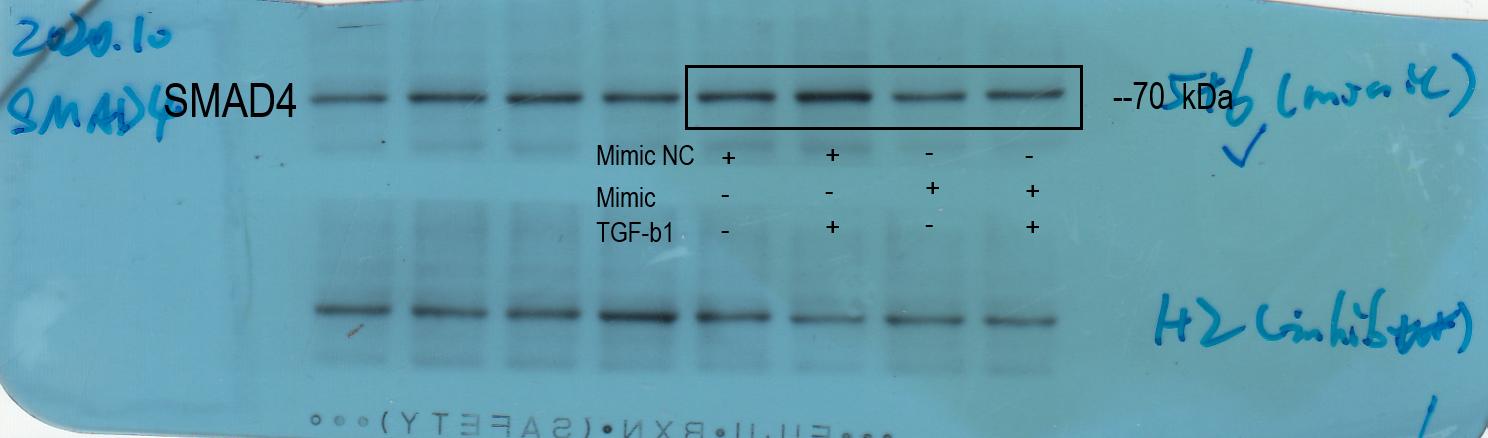

Supplement: Supplementary file 1 [file DataSheet1.ZIP › Supplementary materials/Original source data/uncropped images/Fig.9/Fig.9D SMAD4.tif]

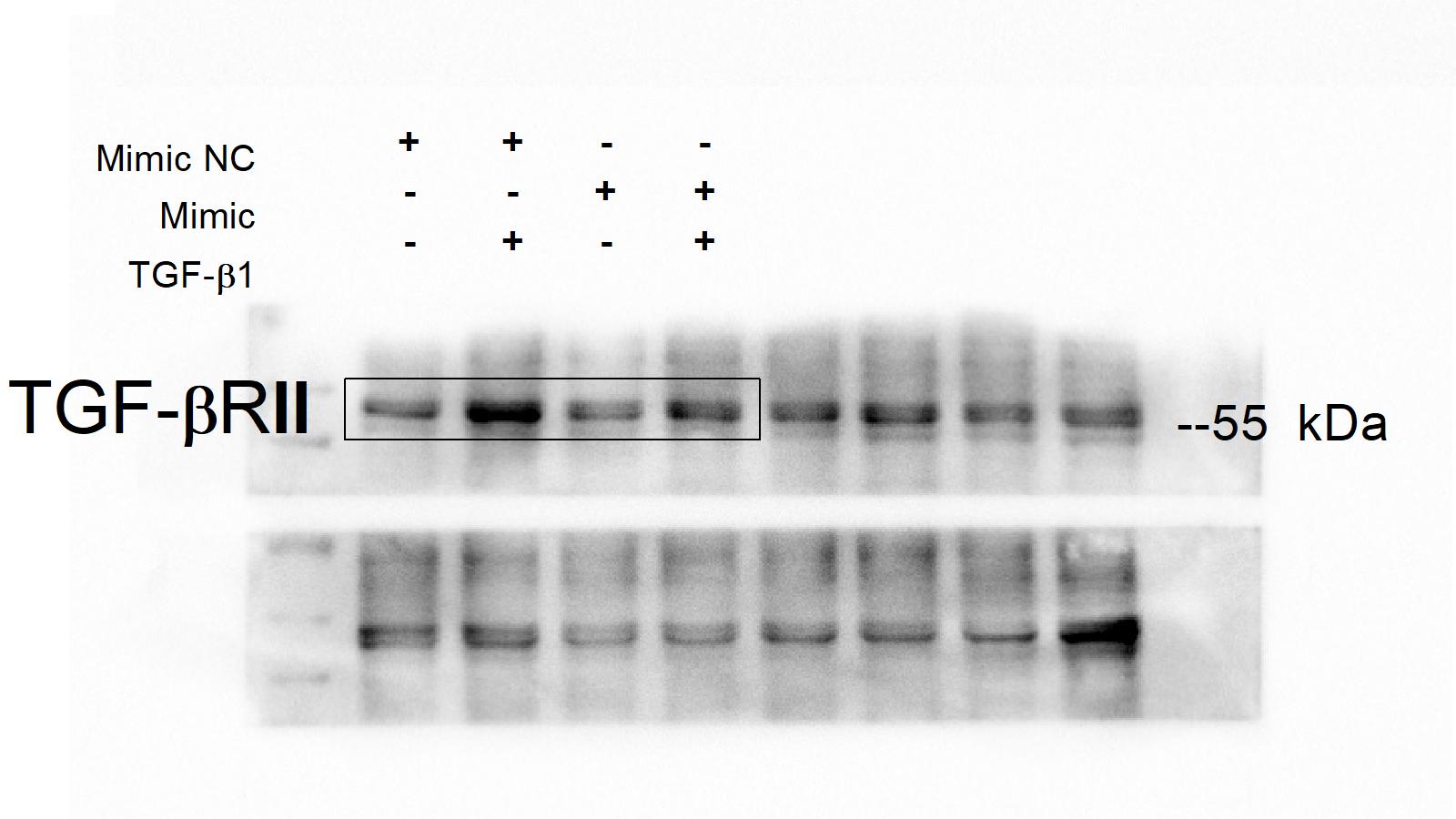

Supplement: Supplementary file 1 [file DataSheet1.ZIP › Supplementary materials/Original source data/uncropped images/Fig.9/Fig.9D TGF-bRII.tiff]

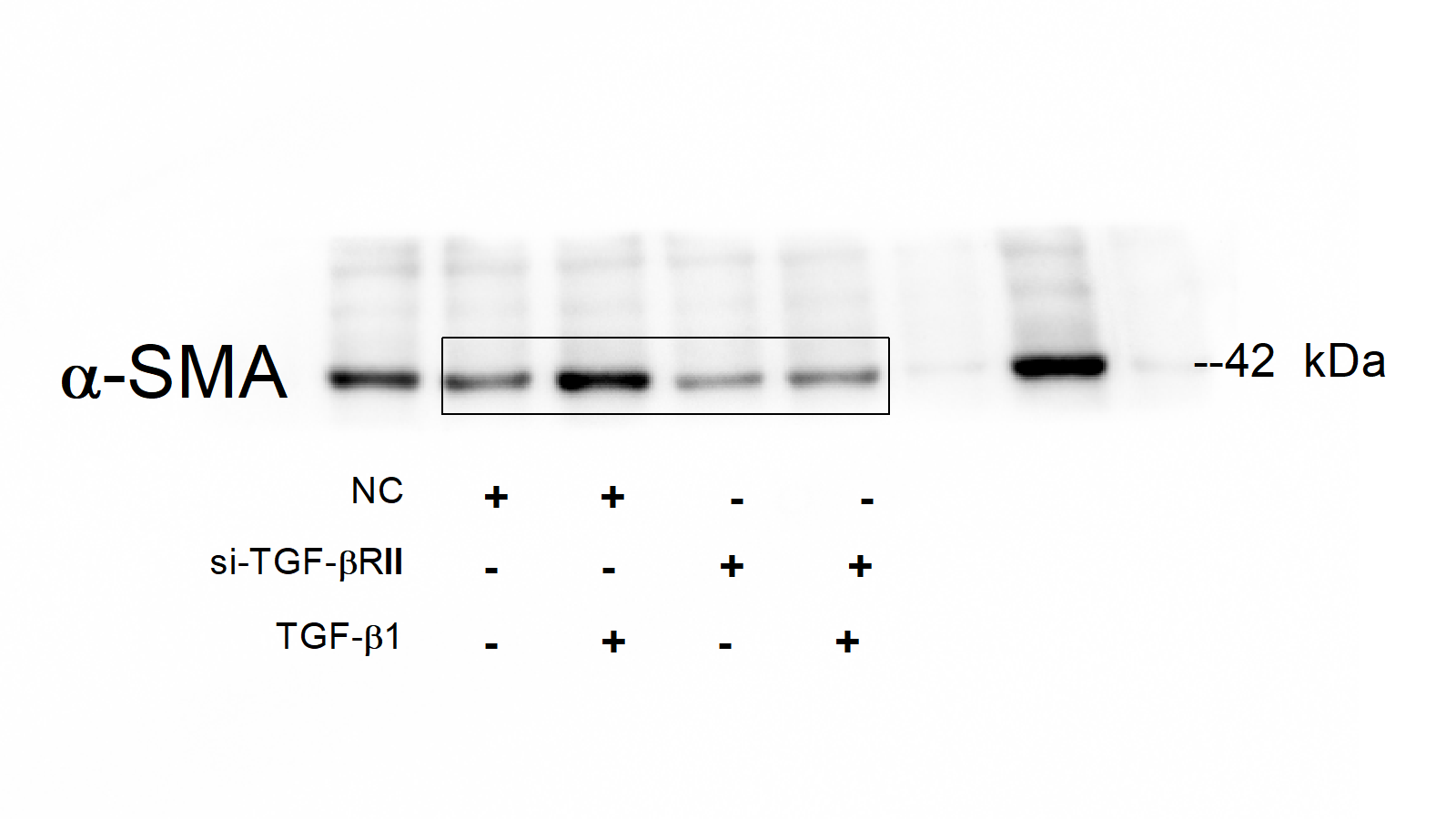

Supplement: Supplementary file 1 [file DataSheet1.ZIP › Supplementary materials/Original source data/uncropped images/Fig.9/Fig.9G a-SMA.tiff]

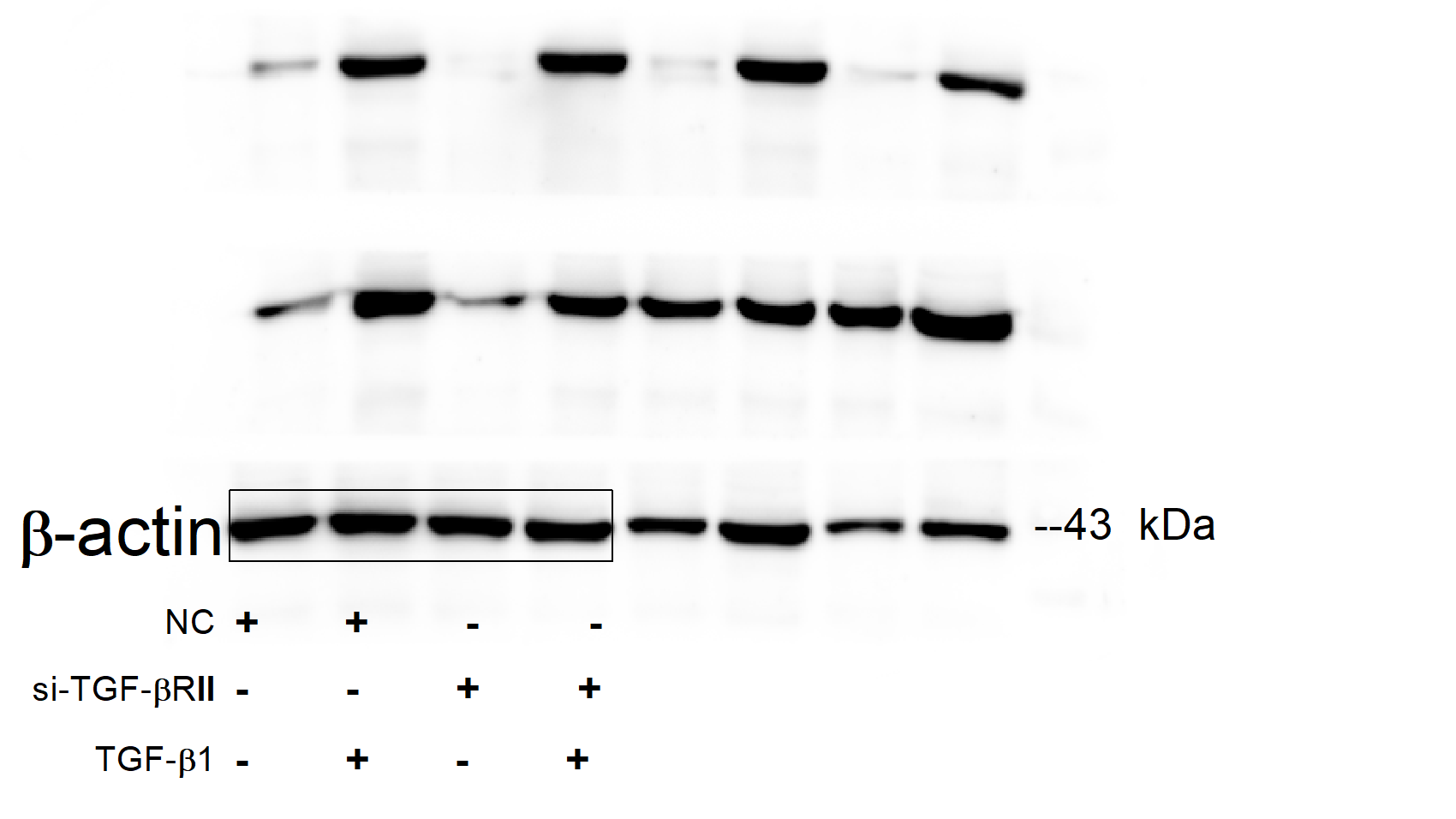

Supplement: Supplementary file 1 [file DataSheet1.ZIP › Supplementary materials/Original source data/uncropped images/Fig.9/Fig.9G b-actin.tiff]

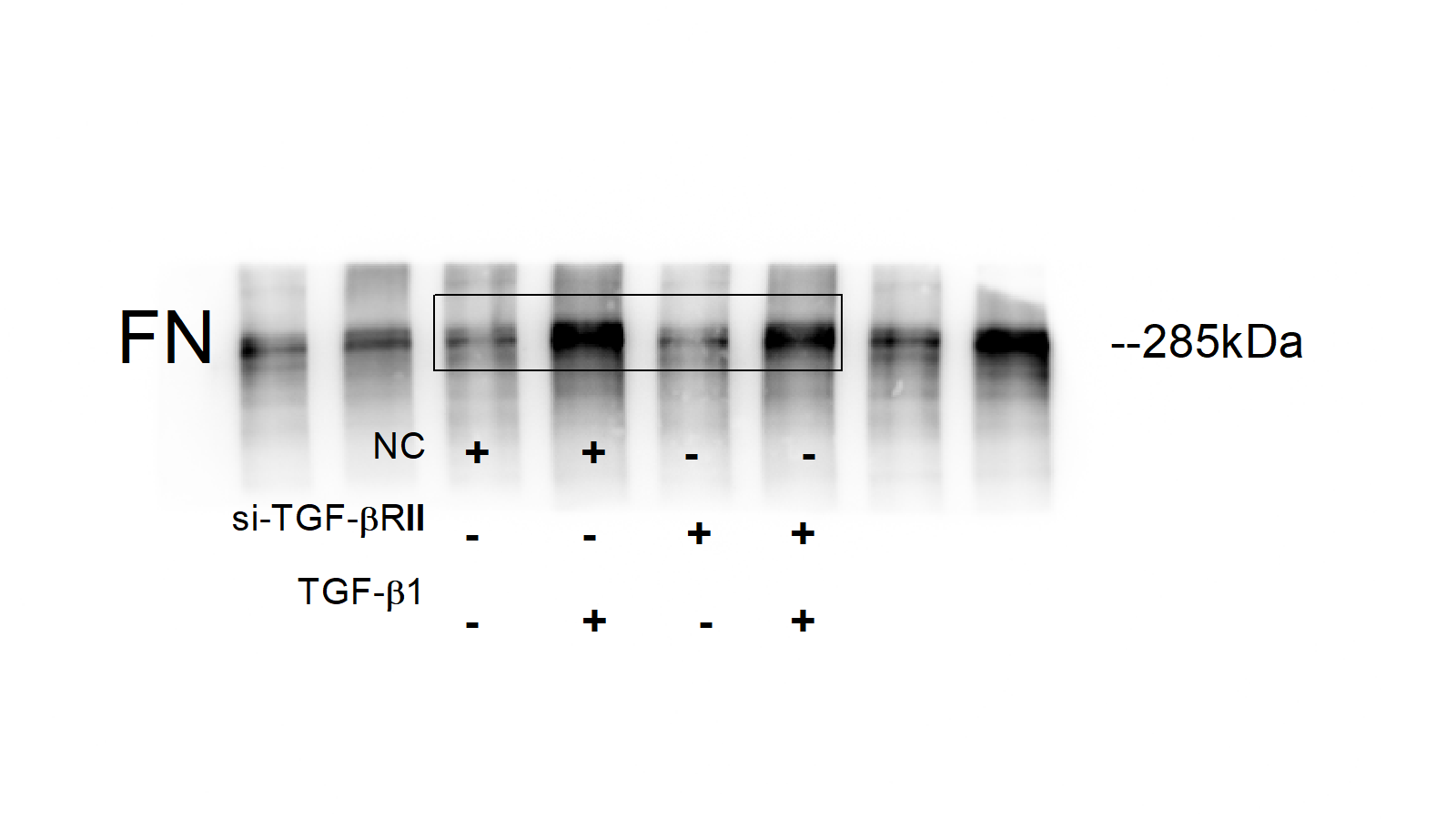

Supplement: Supplementary file 1 [file DataSheet1.ZIP › Supplementary materials/Original source data/uncropped images/Fig.9/Fig.9G FN.tiff]

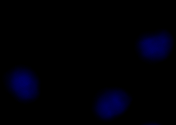

Supplement: Supplementary file 1 [file DataSheet1.ZIP › Supplementary materials/Original source data/uncropped images/Fig.9/Immunofluorescence/Fig.9F Mimic NC+TGF-DAPI.tif]

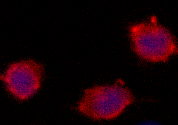

Supplement: Supplementary file 1 [file DataSheet1.ZIP › Supplementary materials/Original source data/uncropped images/Fig.9/Immunofluorescence/Fig.9F Mimic NC+TGF-Merge.tif]

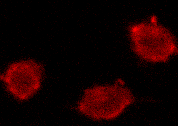

Supplement: Supplementary file 1 [file DataSheet1.ZIP › Supplementary materials/Original source data/uncropped images/Fig.9/Immunofluorescence/Fig.9F Mimic NC+TGF-SMA.tif]

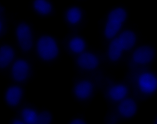

Supplement: Supplementary file 1 [file DataSheet1.ZIP › Supplementary materials/Original source data/uncropped images/Fig.9/Immunofluorescence/Fig.9F Mimic NC-DAPI.tif]

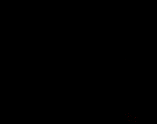

Supplement: Supplementary file 1 [file DataSheet1.ZIP › Supplementary materials/Original source data/uncropped images/Fig.9/Immunofluorescence/Fig.9F Mimic NC-SMA.tif]

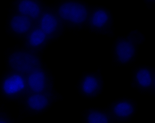

Supplement: Supplementary file 1 [file DataSheet1.ZIP › Supplementary materials/Original source data/uncropped images/Fig.9/Immunofluorescence/Fig.9F Mimic+TGF-DAPI.tif]

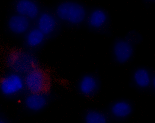

Supplement: Supplementary file 1 [file DataSheet1.ZIP › Supplementary materials/Original source data/uncropped images/Fig.9/Immunofluorescence/Fig.9F Mimic+TGF-Merge.tif]

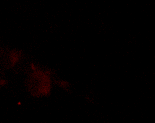

Supplement: Supplementary file 1 [file DataSheet1.ZIP › Supplementary materials/Original source data/uncropped images/Fig.9/Immunofluorescence/Fig.9F Mimic+TGF-SMA.tif]

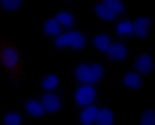

Supplement: Supplementary file 1 [file DataSheet1.ZIP › Supplementary materials/Original source data/uncropped images/Fig.9/Immunofluorescence/Fig.9F Mimic-DAPI.tif]

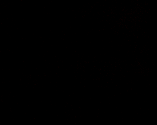

Supplement: Supplementary file 1 [file DataSheet1.ZIP › Supplementary materials/Original source data/uncropped images/Fig.9/Immunofluorescence/Fig.9F Mimic-SMA.tif]

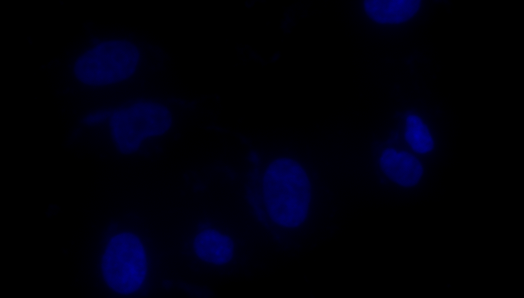

Supplement: Supplementary file 1 [file DataSheet1.ZIP › Supplementary materials/Original source data/uncropped images/Fig.S2/Fig.S2C DAPI.tif]

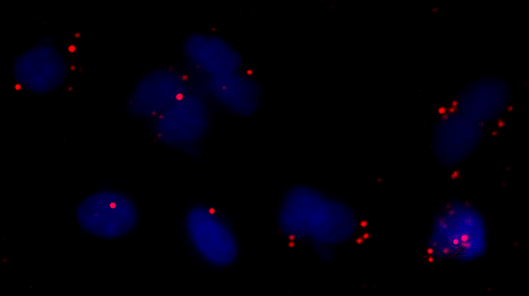

Supplement: Supplementary file 1 [file DataSheet1.ZIP › Supplementary materials/Original source data/uncropped images/Fig.S2/Fig.S2D CY3-miR-130a-3p.tif]

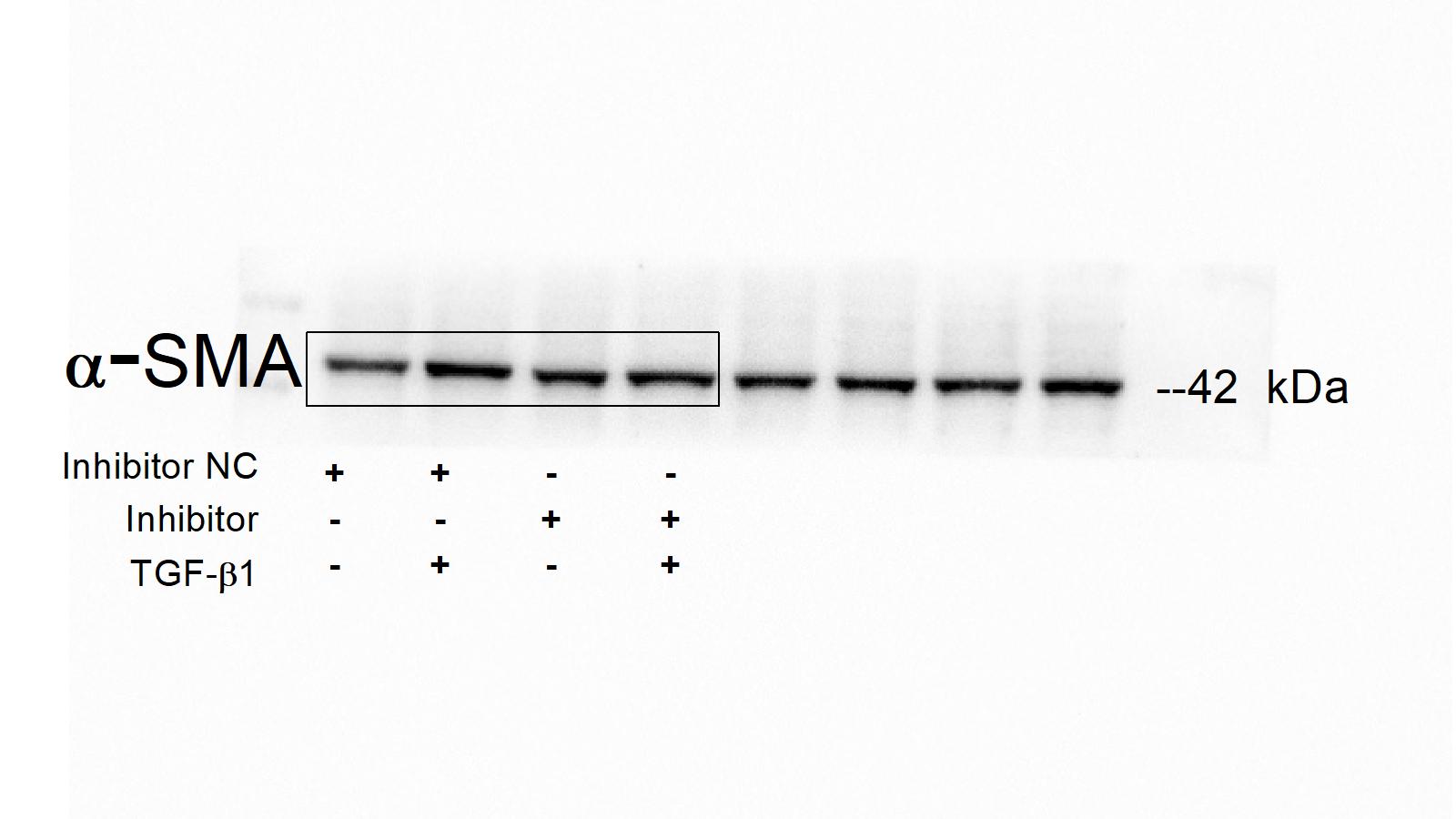

Supplement: Supplementary file 1 [file DataSheet1.ZIP › Supplementary materials/Original source data/uncropped images/Fig.S3/Fig.S3 a-SMA.tiff]

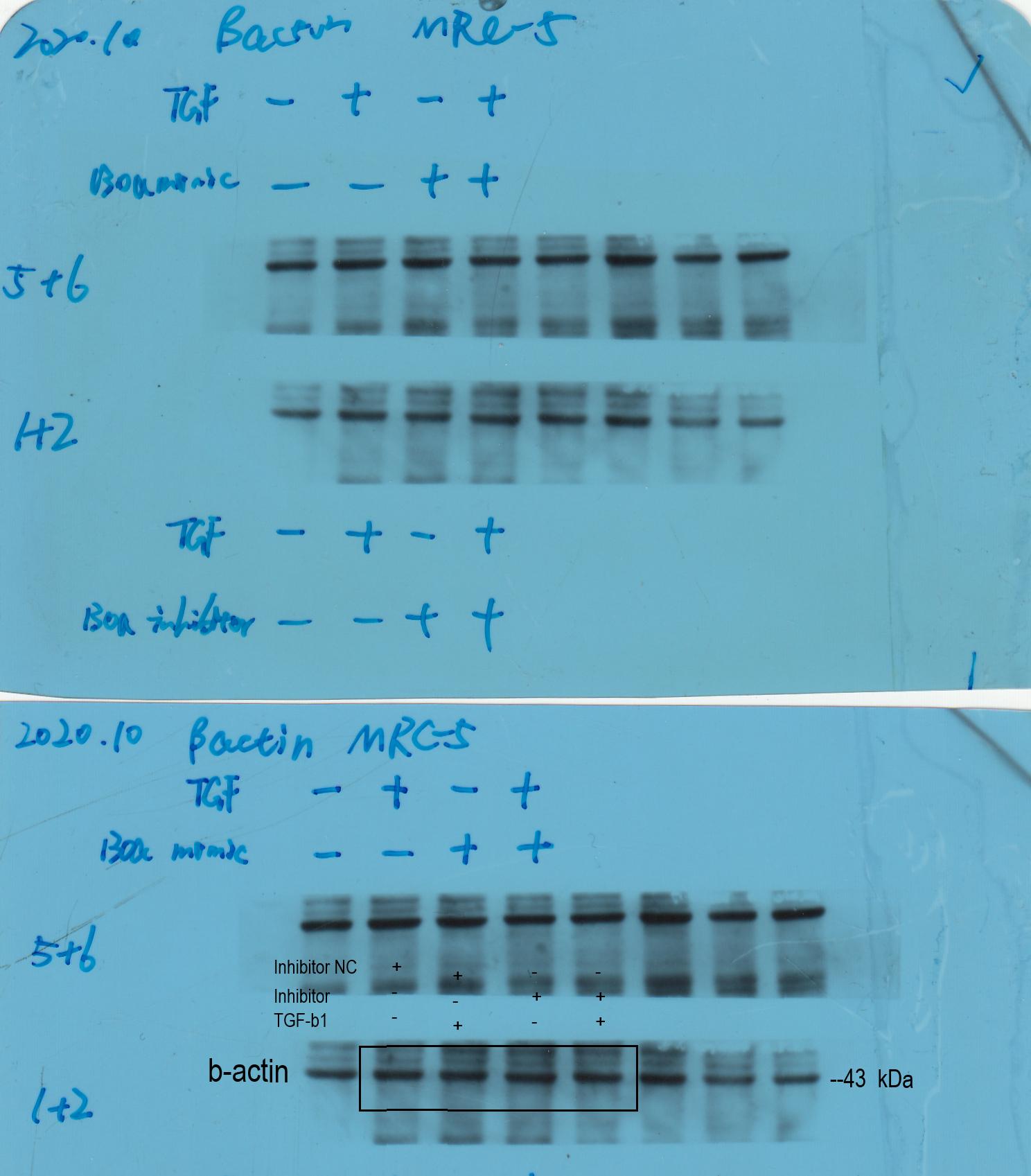

Supplement: Supplementary file 1 [file DataSheet1.ZIP › Supplementary materials/Original source data/uncropped images/Fig.S3/Fig.S3 b-actin.tiff]

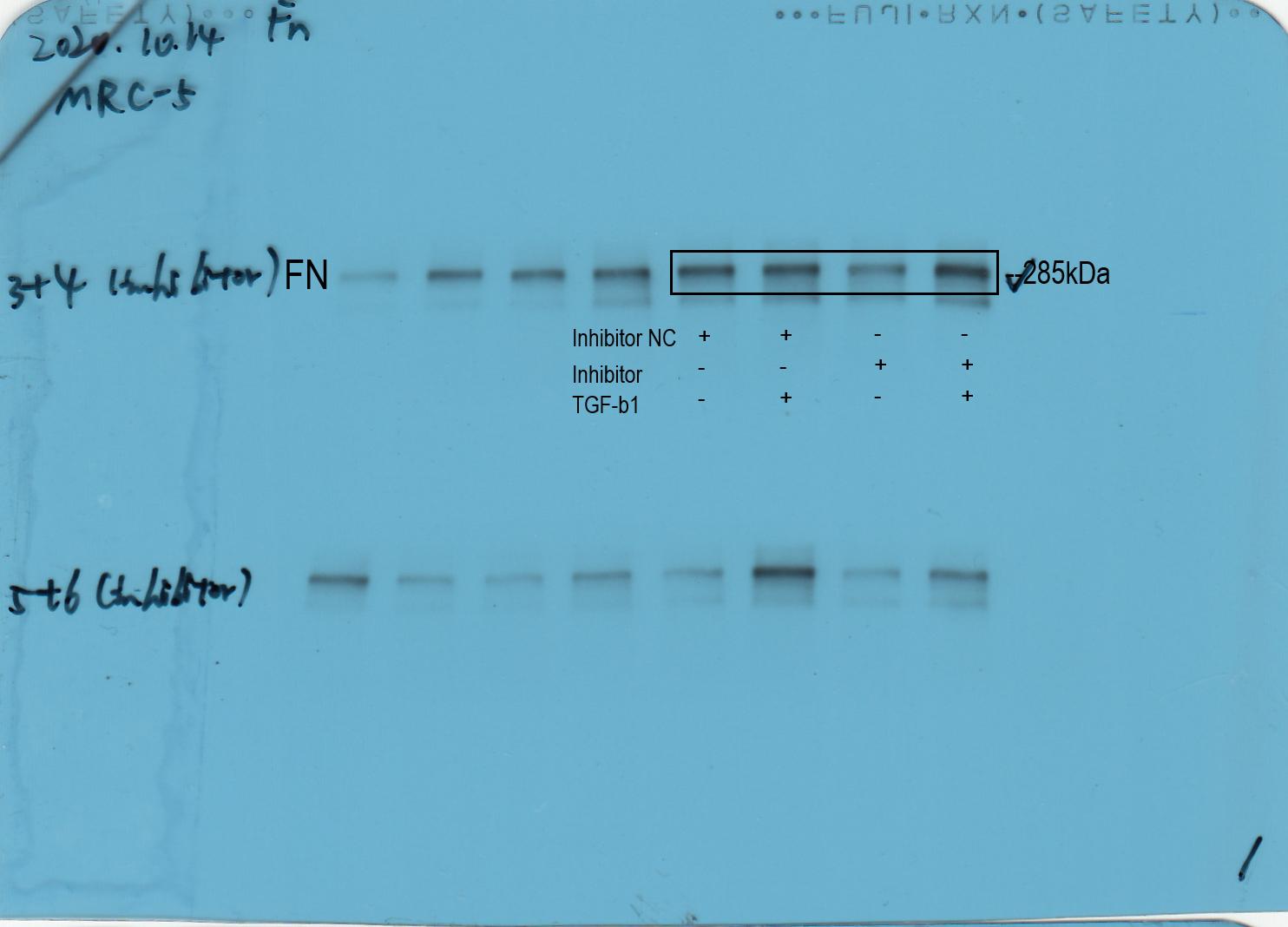

Supplement: Supplementary file 1 [file DataSheet1.ZIP › Supplementary materials/Original source data/uncropped images/Fig.S3/Fig.S3 FN.tiff]

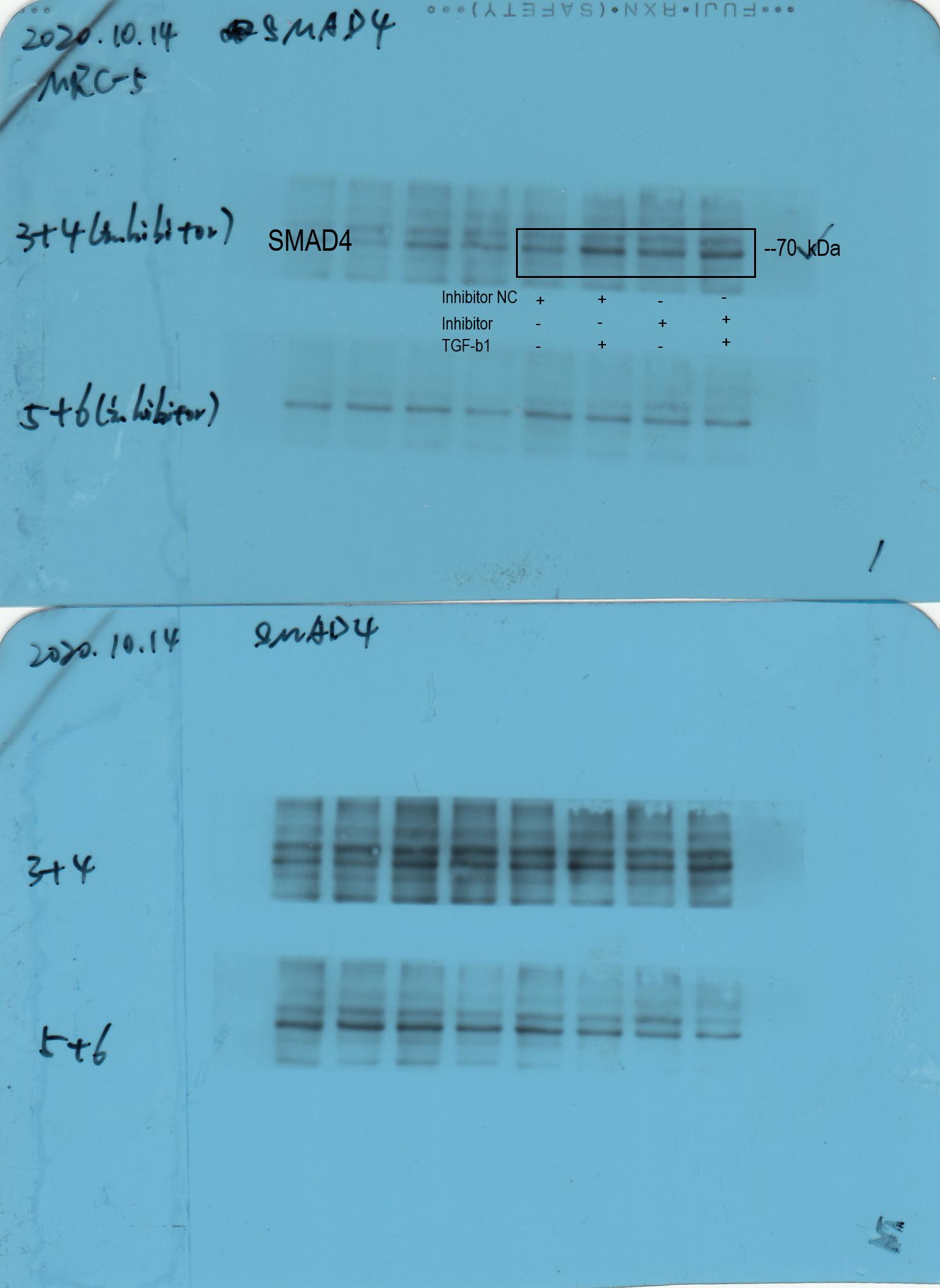

Supplement: Supplementary file 1 [file DataSheet1.ZIP › Supplementary materials/Original source data/uncropped images/Fig.S3/Fig.S3 SMAD4.tiff]

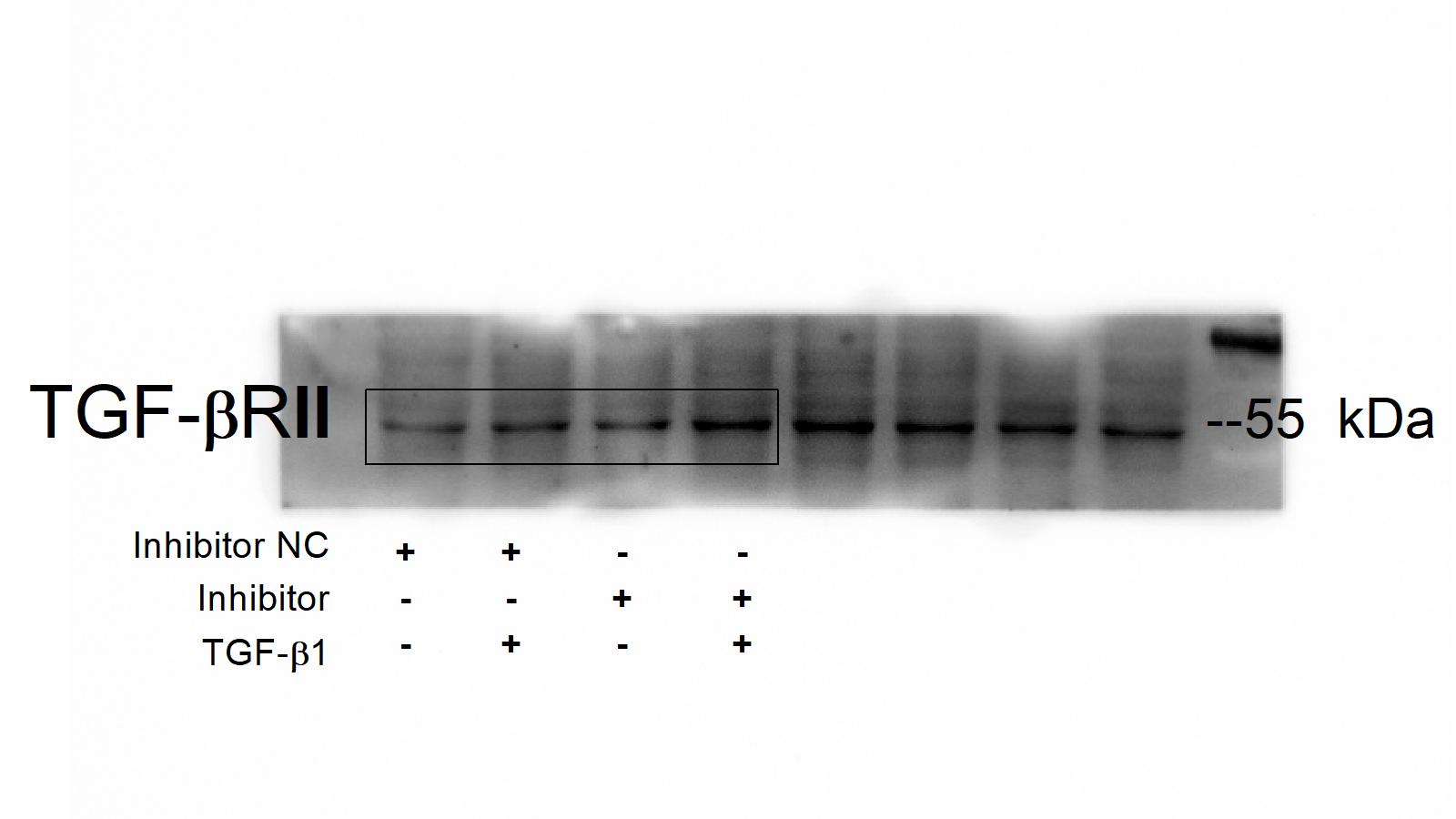

Supplement: Supplementary file 1 [file DataSheet1.ZIP › Supplementary materials/Original source data/uncropped images/Fig.S3/Fig.S3 TGF-bRII.tiff]

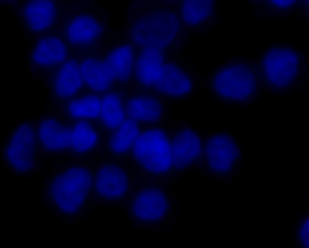

Supplement: Supplementary file 1 [file DataSheet1.ZIP › Supplementary materials/Original source data/uncropped images/Fig.S4/Fig.S4 Inhibitor NC+TGF-DAPI.tif]

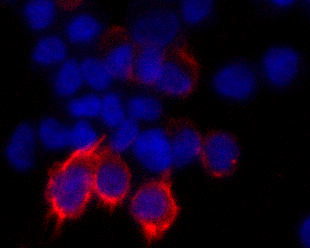

Supplement: Supplementary file 1 [file DataSheet1.ZIP › Supplementary materials/Original source data/uncropped images/Fig.S4/Fig.S4 Inhibitor NC+TGF-Merge.tif]

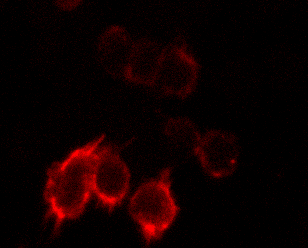

Supplement: Supplementary file 1 [file DataSheet1.ZIP › Supplementary materials/Original source data/uncropped images/Fig.S4/Fig.S4 Inhibitor NC+TGF-SMA.tif]

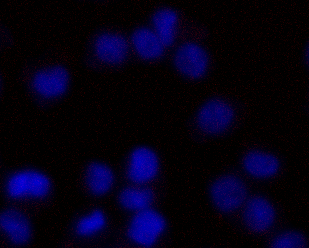

Supplement: Supplementary file 1 [file DataSheet1.ZIP › Supplementary materials/Original source data/uncropped images/Fig.S4/Fig.S4 Inhibitor NC-DAPI.tif]

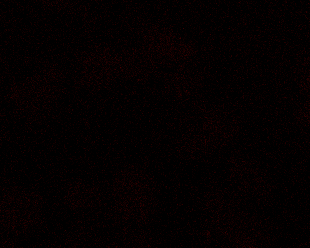

Supplement: Supplementary file 1 [file DataSheet1.ZIP › Supplementary materials/Original source data/uncropped images/Fig.S4/Fig.S4 Inhibitor NC-SMA.tif]

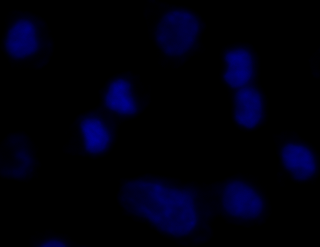

Supplement: Supplementary file 1 [file DataSheet1.ZIP › Supplementary materials/Original source data/uncropped images/Fig.S4/Fig.S4 Inhibitor+TGF-DAPI.tif]

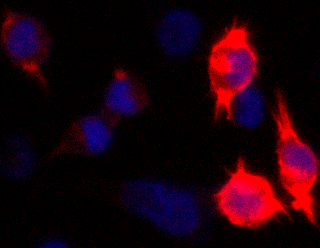

Supplement: Supplementary file 1 [file DataSheet1.ZIP › Supplementary materials/Original source data/uncropped images/Fig.S4/Fig.S4 Inhibitor+TGF-Merge.tif]

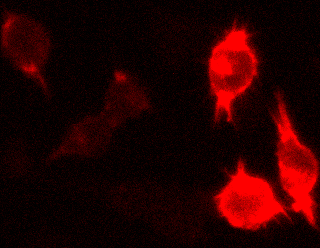

Supplement: Supplementary file 1 [file DataSheet1.ZIP › Supplementary materials/Original source data/uncropped images/Fig.S4/Fig.S4 Inhibitor+TGF-SMA.tif]

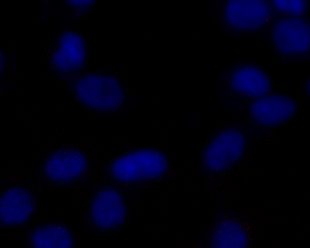

Supplement: Supplementary file 1 [file DataSheet1.ZIP › Supplementary materials/Original source data/uncropped images/Fig.S4/Fig.S4 Inhibitor-DAPI.tif]

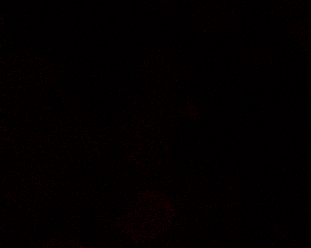

Supplement: Supplementary file 1 [file DataSheet1.ZIP › Supplementary materials/Original source data/uncropped images/Fig.S4/Fig.S4 Inhibitor-SMA.tif]
