# Supplementary figures and images for: MiR-130a-3p Alleviates Inflammatory and Fibrotic Phases of Pulmonary Fibrosis Through Proinflammatory Factor TNF-α and Profibrogenic Receptor TGF-βRII (part 1 of 2)
Source: Front Pharmacol. 2022 Mar 30;13:863646. doi: 10.3389/fphar.2022.863646 (PMC9006815; doi:10.3389/fphar.2022.863646)

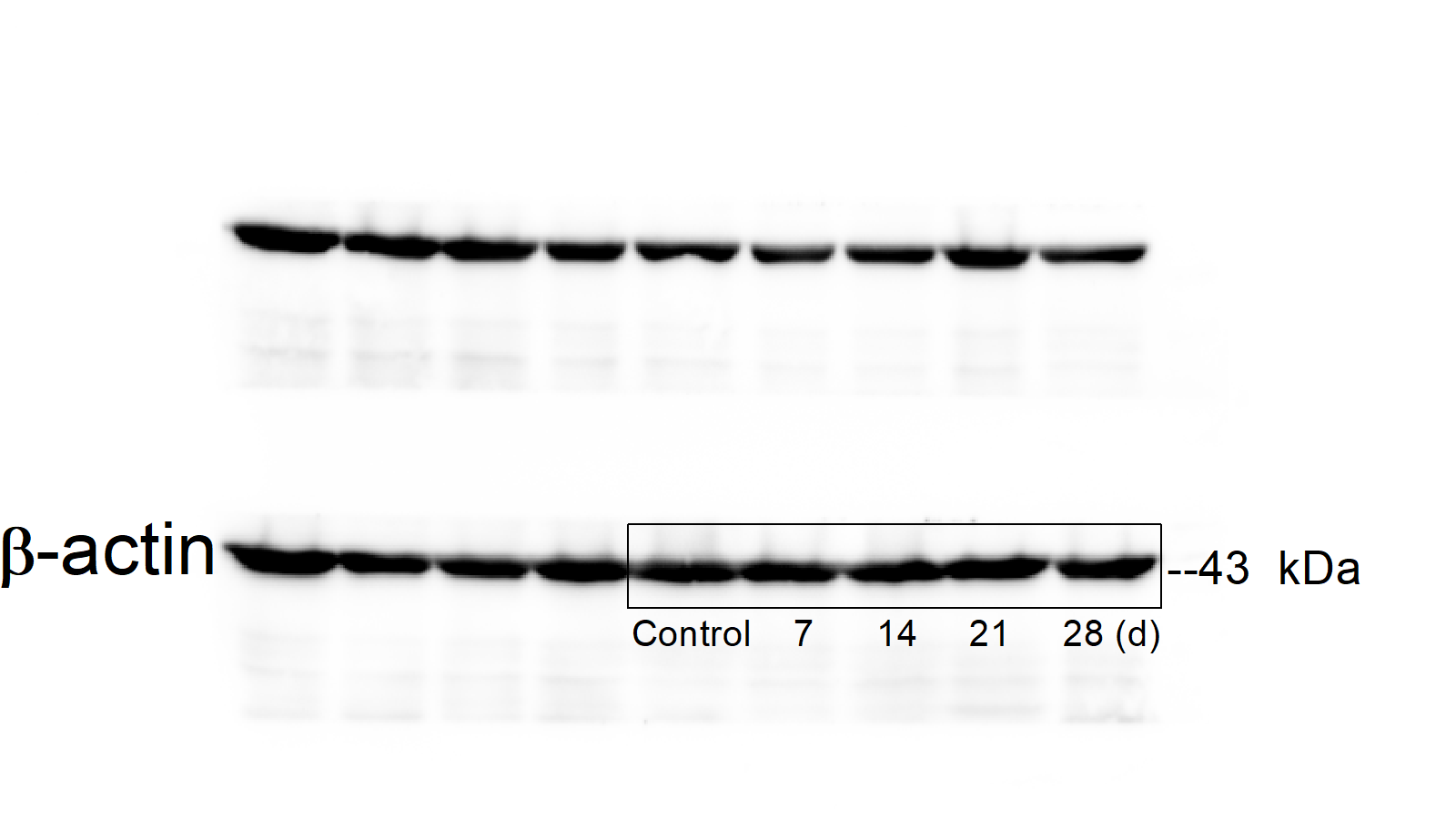

Supplement: Supplementary file 1 [file DataSheet1.ZIP › Supplementary materials/Original source data/uncropped images/Fig.2/Fig.2C b-actin.tiff]

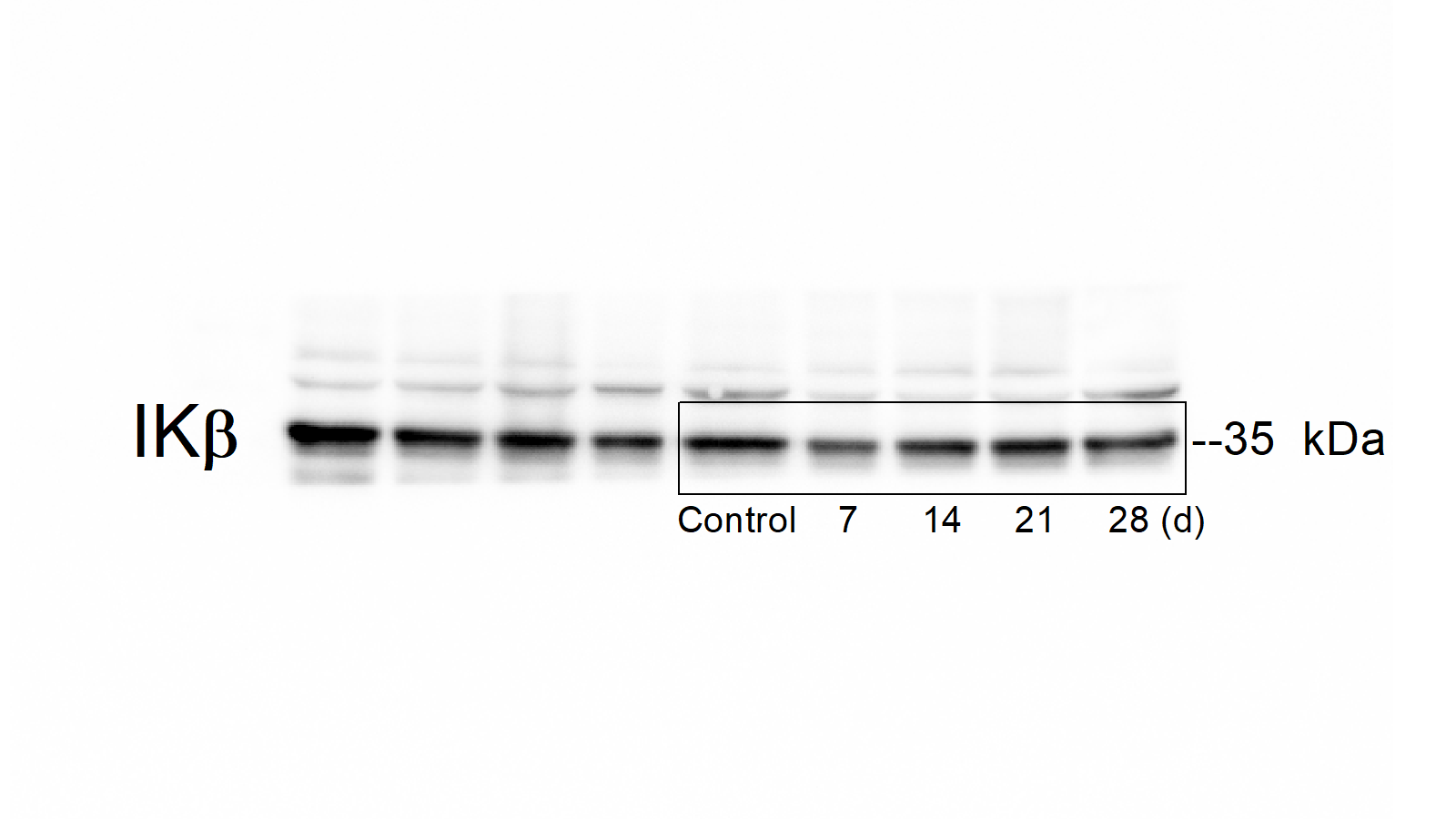

Supplement: Supplementary file 1 [file DataSheet1.ZIP › Supplementary materials/Original source data/uncropped images/Fig.2/Fig.2C IKb.tiff]

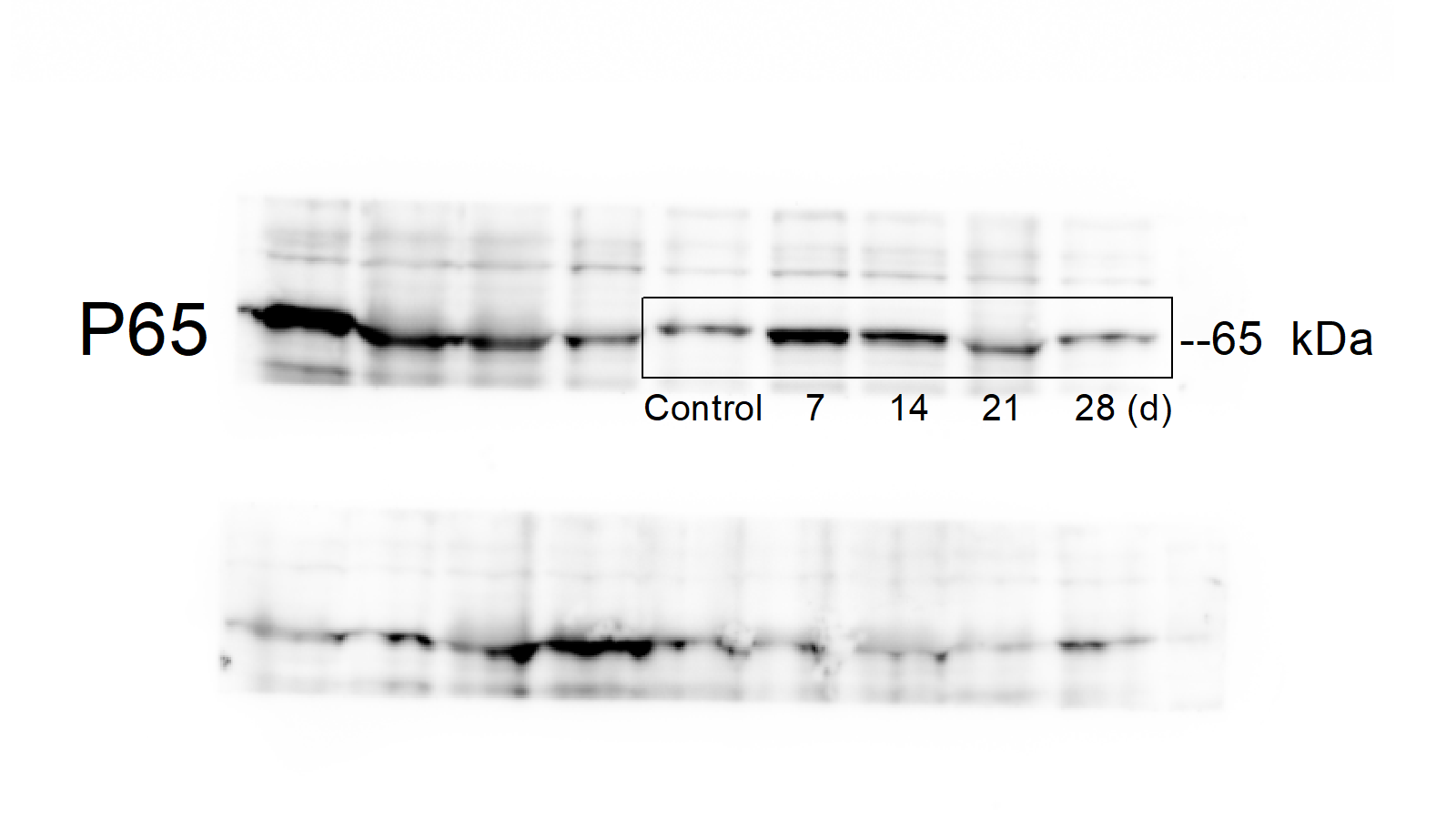

Supplement: Supplementary file 1 [file DataSheet1.ZIP › Supplementary materials/Original source data/uncropped images/Fig.2/Fig.2C P65.tiff]

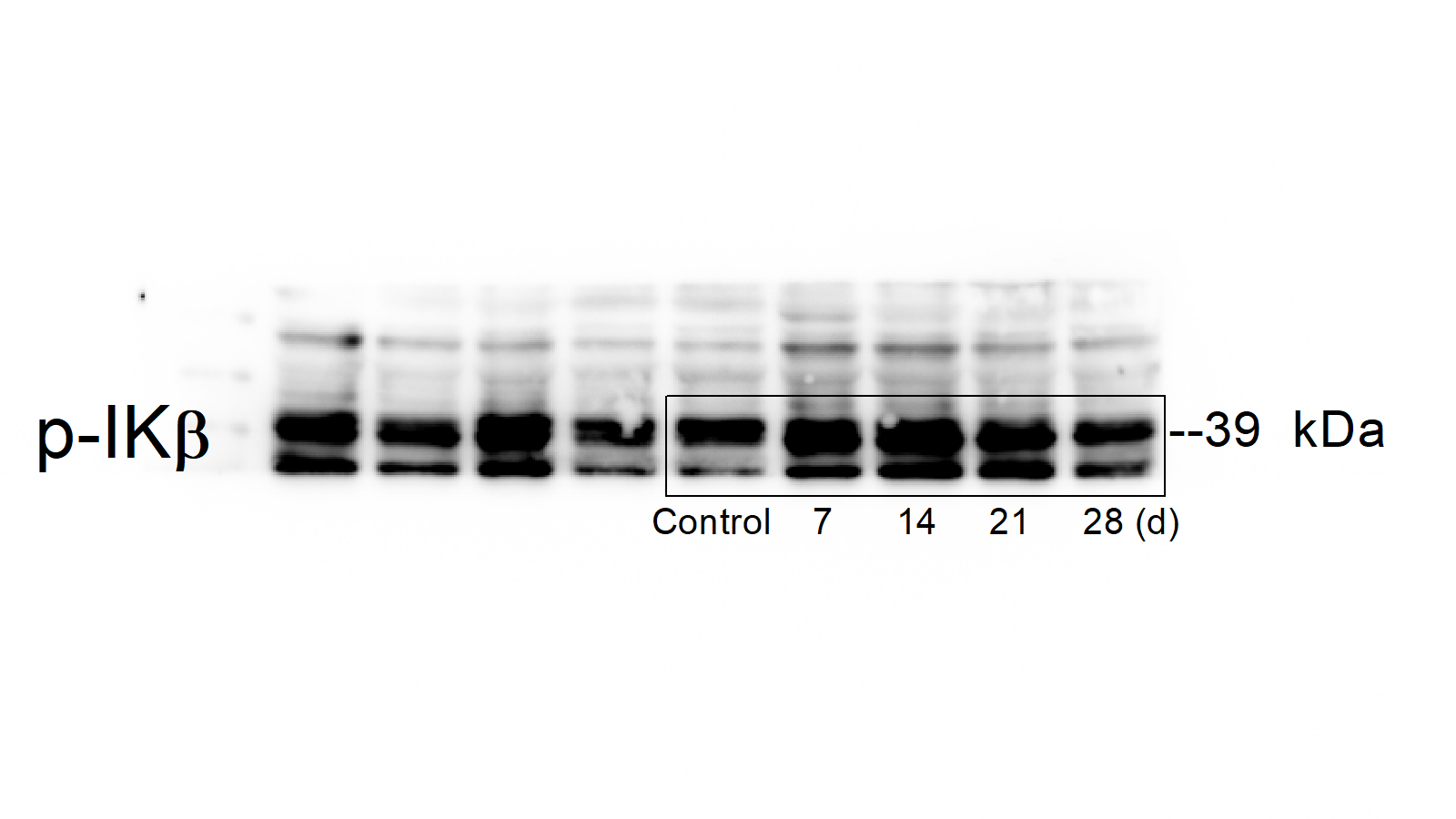

Supplement: Supplementary file 1 [file DataSheet1.ZIP › Supplementary materials/Original source data/uncropped images/Fig.2/Fig.2C p-IKb.tiff]

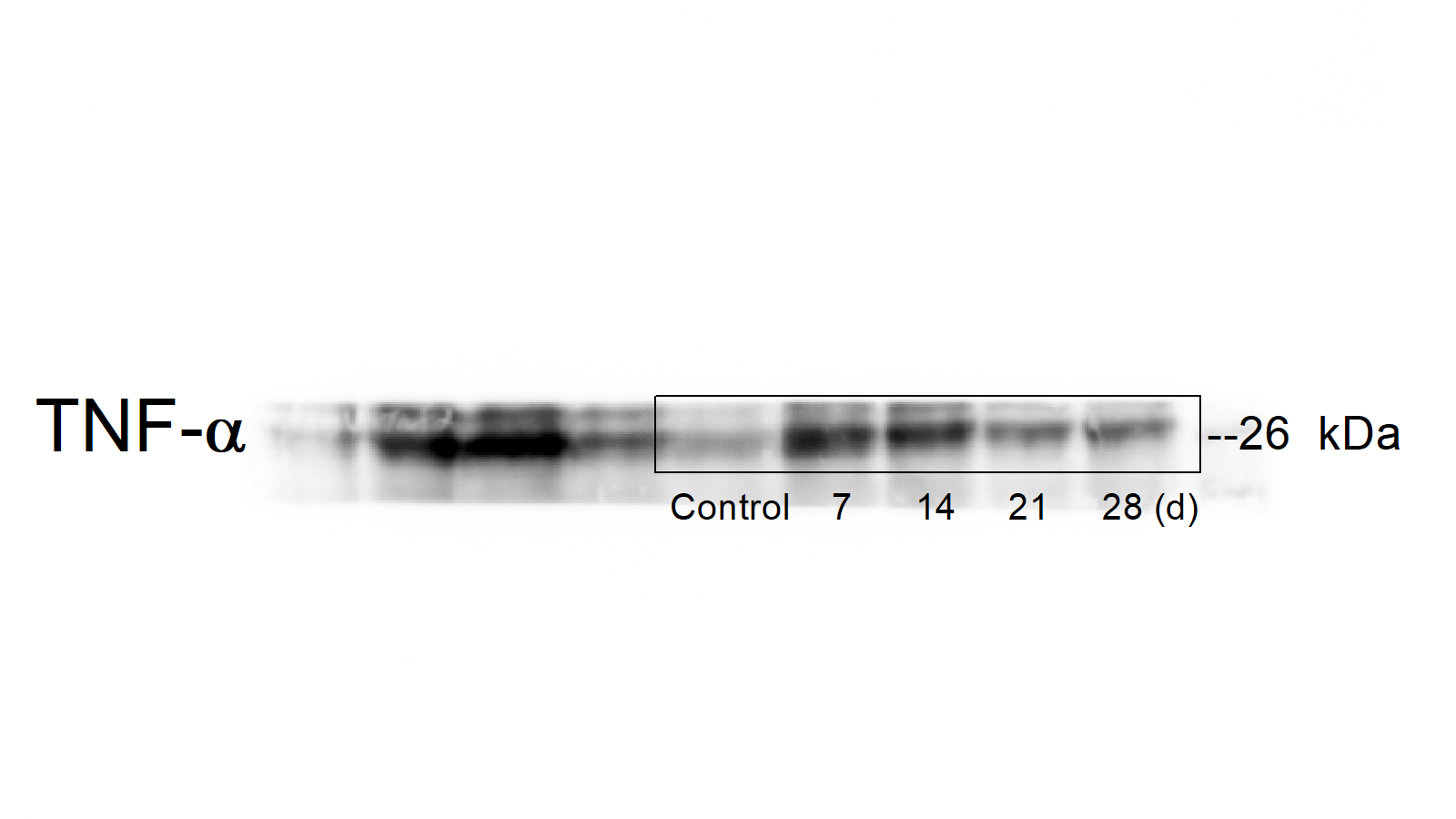

Supplement: Supplementary file 1 [file DataSheet1.ZIP › Supplementary materials/Original source data/uncropped images/Fig.2/Fig.2C TNF-a.tiff]

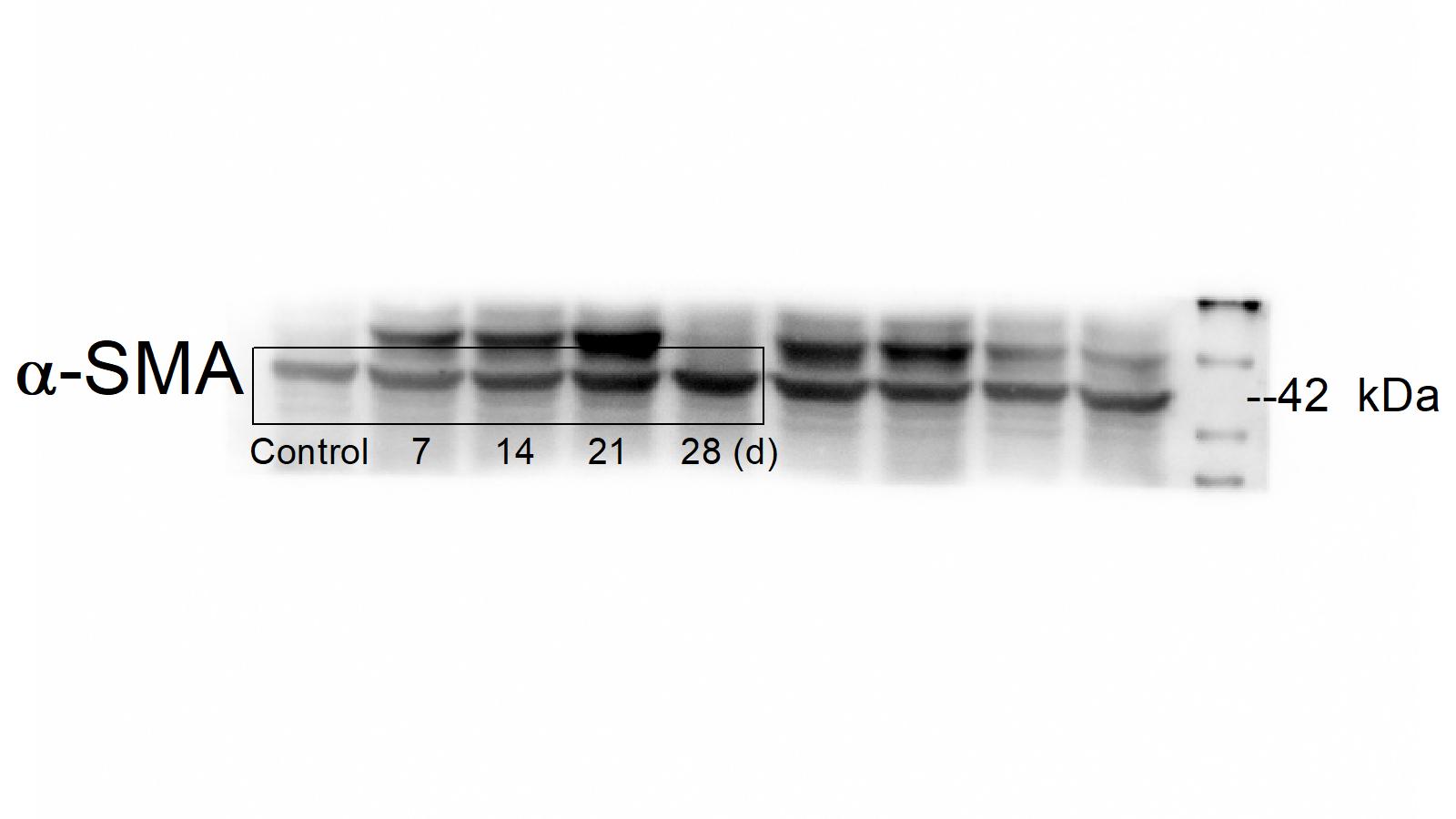

Supplement: Supplementary file 1 [file DataSheet1.ZIP › Supplementary materials/Original source data/uncropped images/Fig.2/Fig.2E a-SMA.tiff]

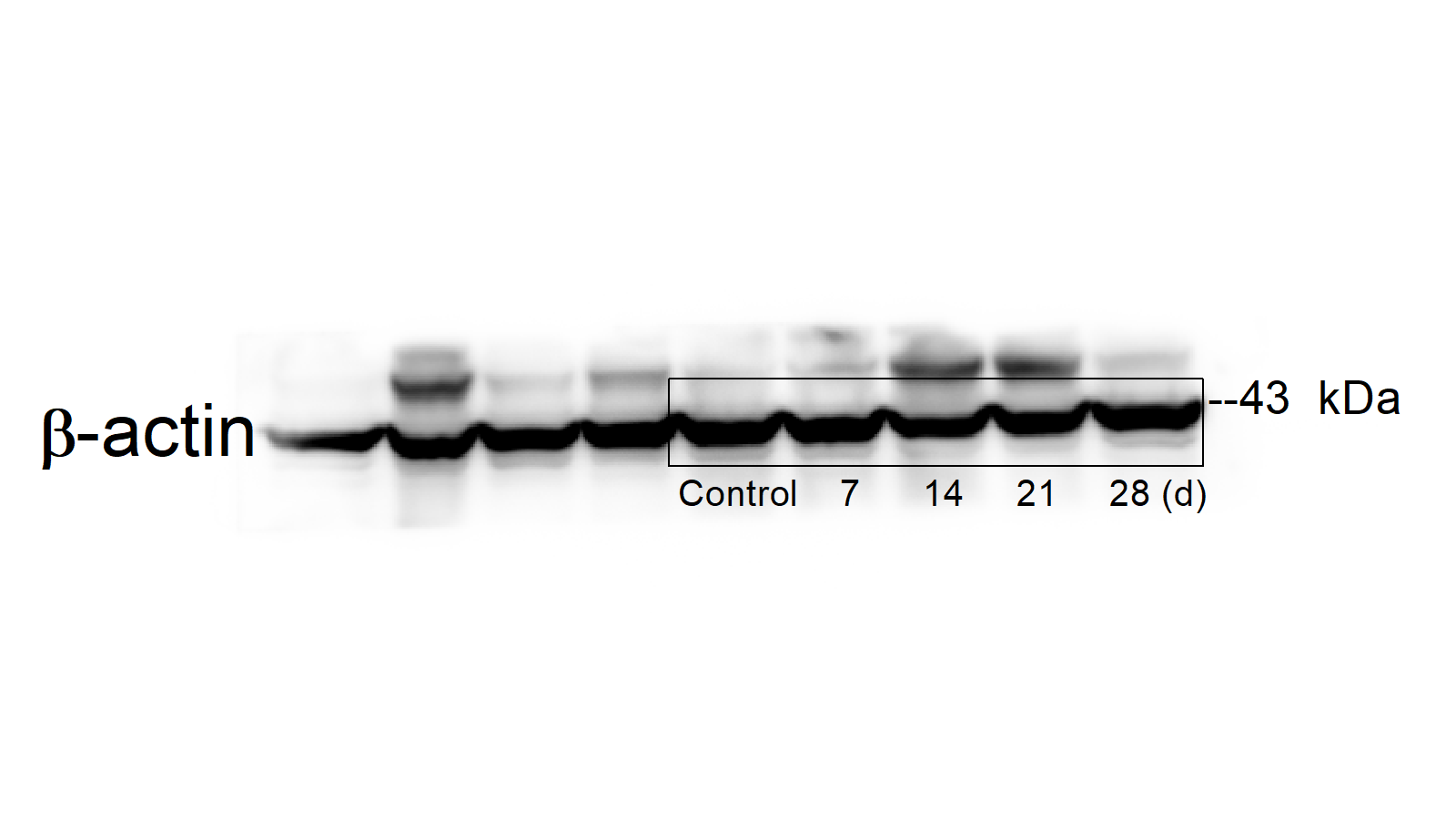

Supplement: Supplementary file 1 [file DataSheet1.ZIP › Supplementary materials/Original source data/uncropped images/Fig.2/Fig.2E b-actin.tiff]

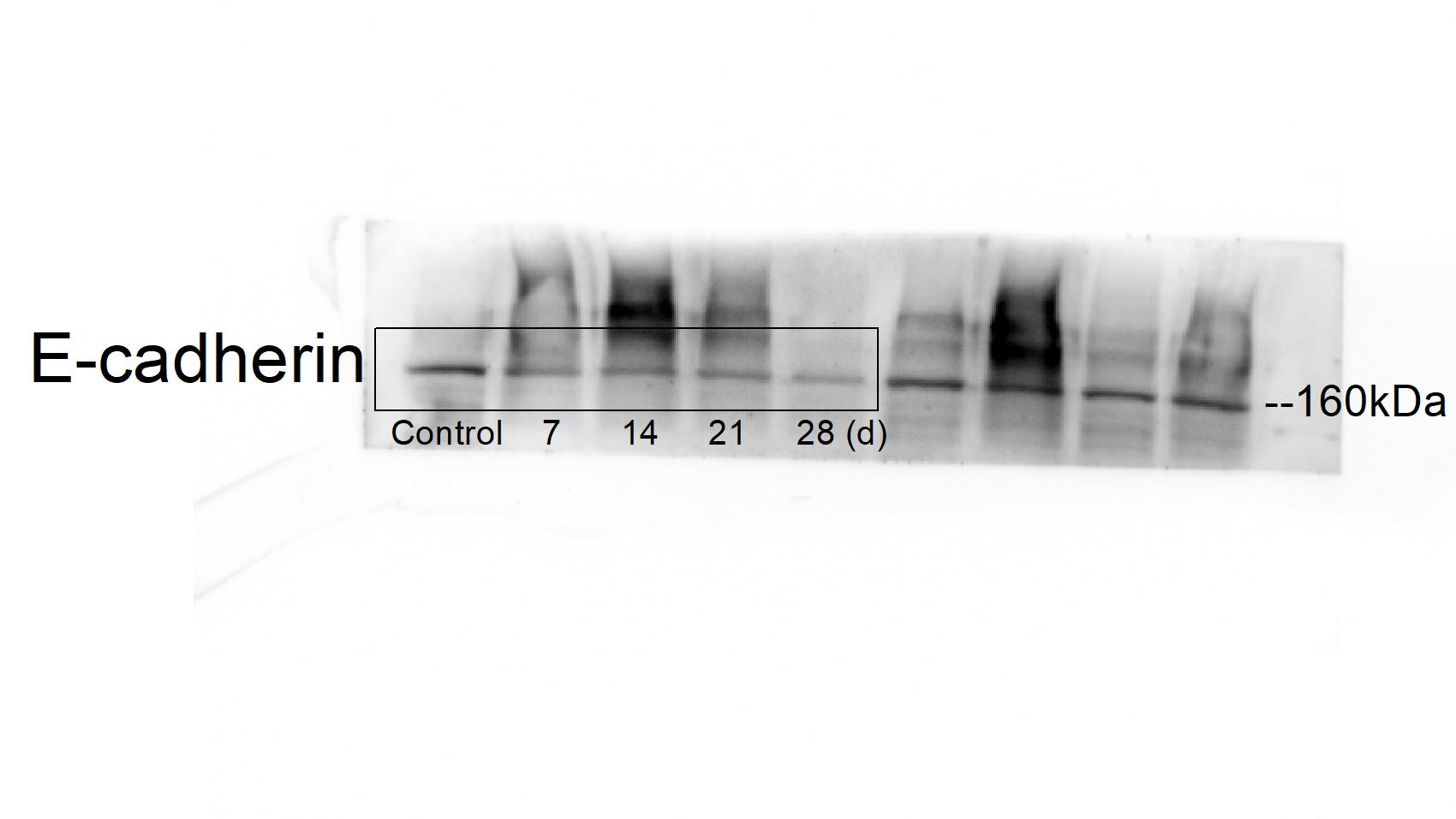

Supplement: Supplementary file 1 [file DataSheet1.ZIP › Supplementary materials/Original source data/uncropped images/Fig.2/Fig.2E E-cadherin.tiff]

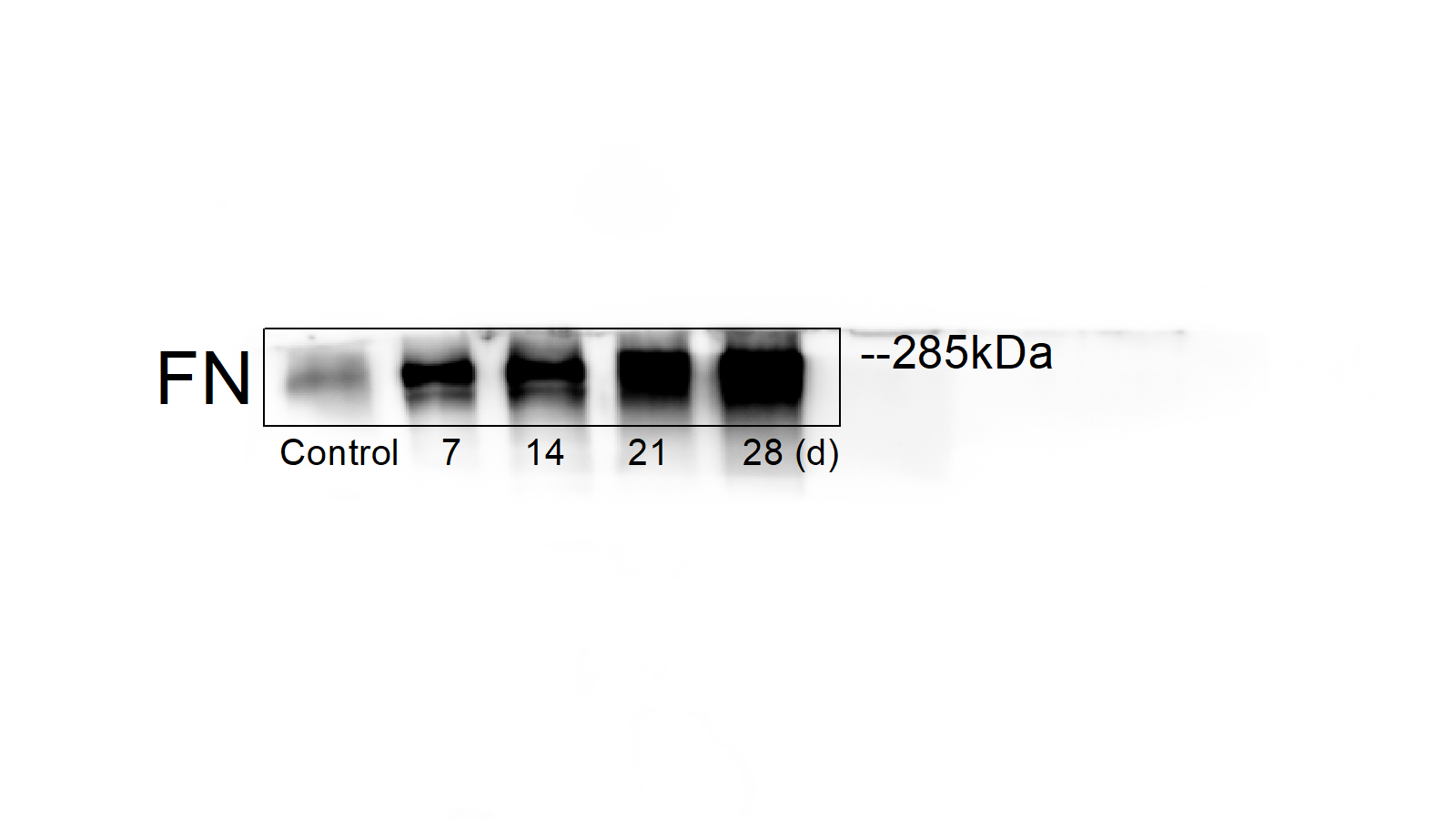

Supplement: Supplementary file 1 [file DataSheet1.ZIP › Supplementary materials/Original source data/uncropped images/Fig.2/Fig.2E FN.tiff]

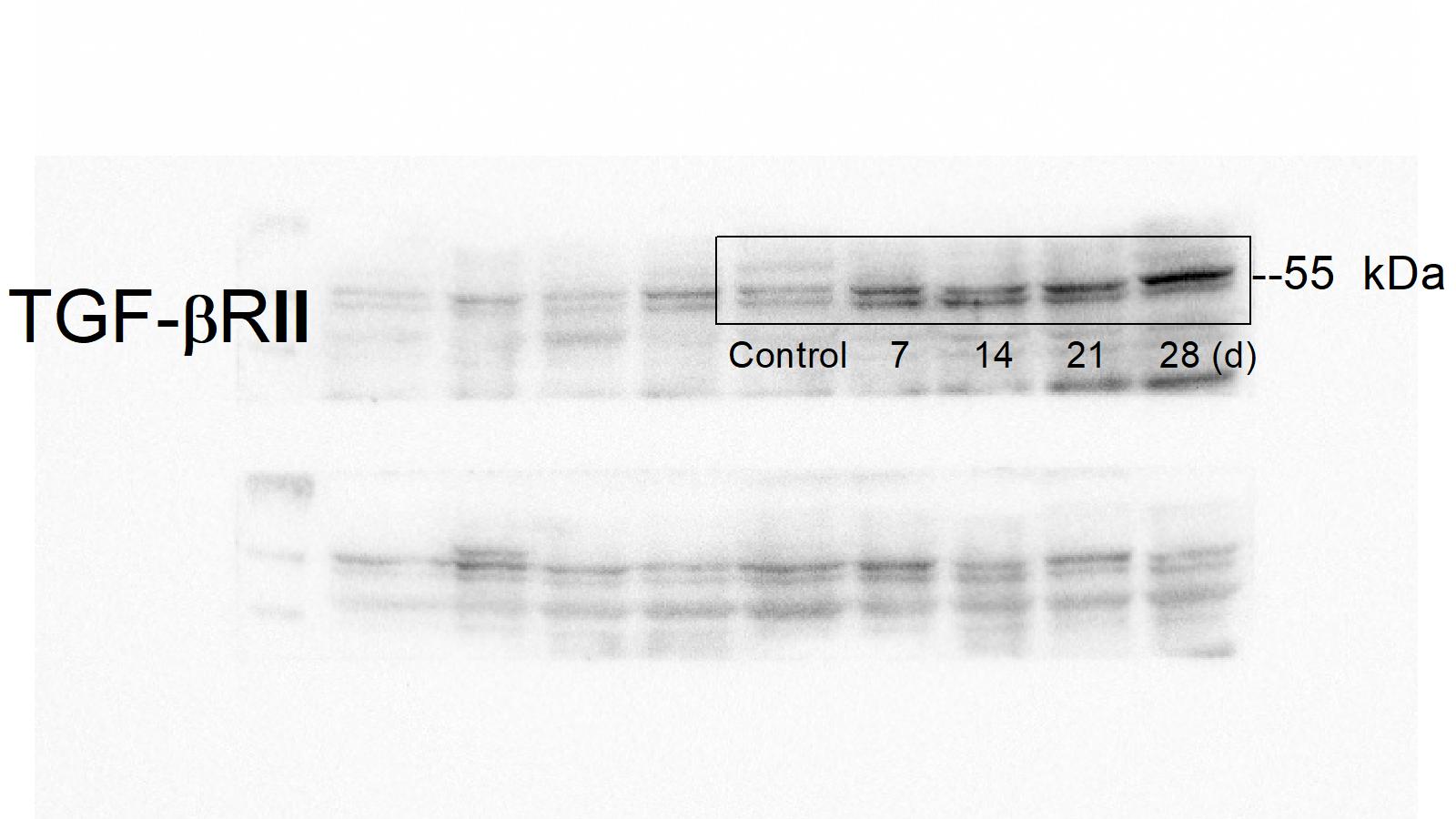

Supplement: Supplementary file 1 [file DataSheet1.ZIP › Supplementary materials/Original source data/uncropped images/Fig.2/Fig.2E TGF-bRII.tiff]

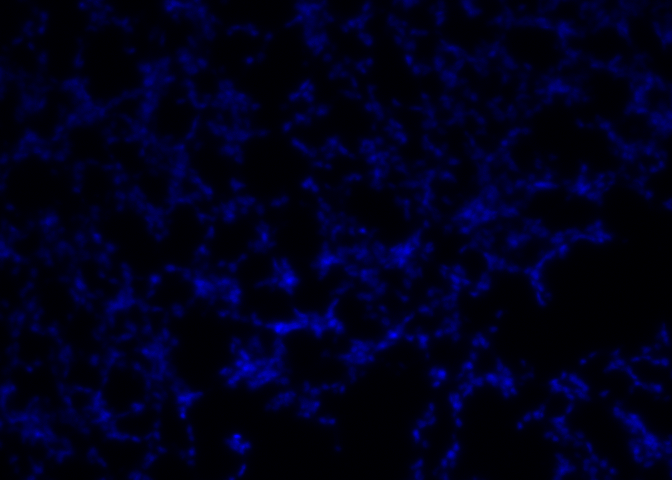

Supplement: Supplementary file 1 [file DataSheet1.ZIP › Supplementary materials/Original source data/uncropped images/Fig.3/Fig.3D agomiR-DAPI.tif]

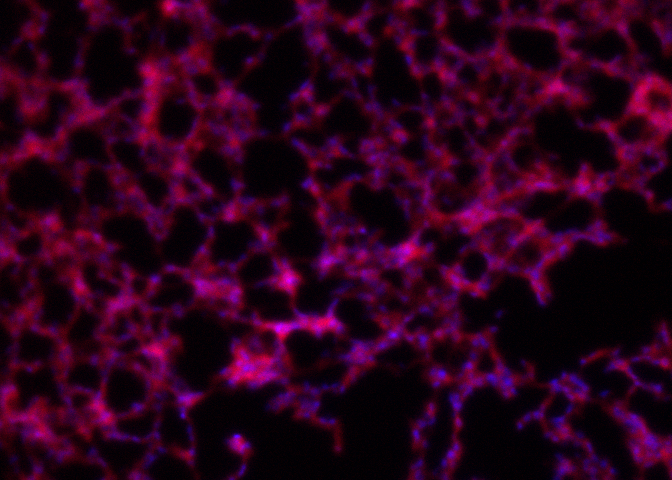

Supplement: Supplementary file 1 [file DataSheet1.ZIP › Supplementary materials/Original source data/uncropped images/Fig.3/Fig.3D agomiR-Merge.tif]

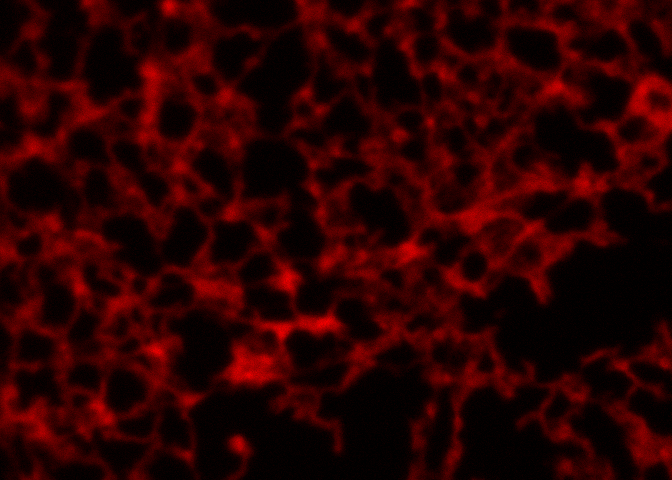

Supplement: Supplementary file 1 [file DataSheet1.ZIP › Supplementary materials/Original source data/uncropped images/Fig.3/Fig.3D agomiR-miR-130a-3p.tif]

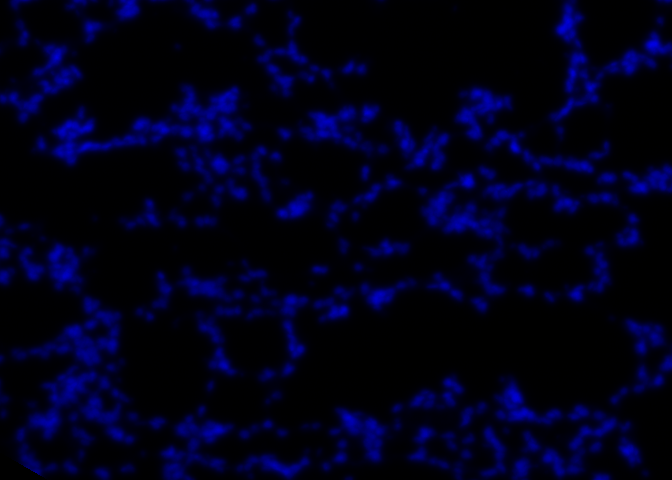

Supplement: Supplementary file 1 [file DataSheet1.ZIP › Supplementary materials/Original source data/uncropped images/Fig.3/Fig.3D BLM+agomiR-DAPI.tif]

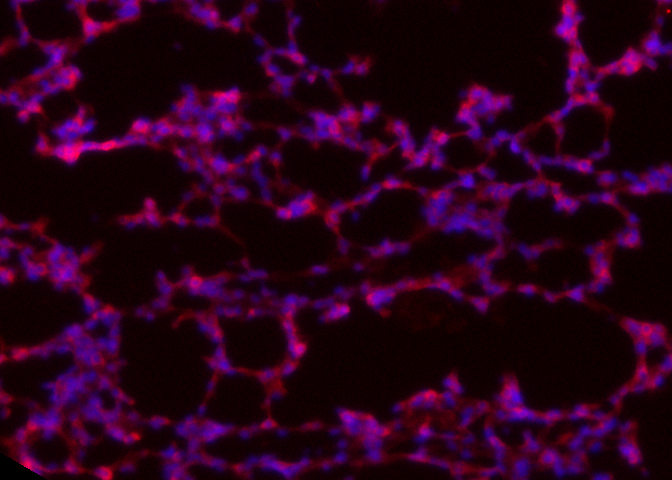

Supplement: Supplementary file 1 [file DataSheet1.ZIP › Supplementary materials/Original source data/uncropped images/Fig.3/Fig.3D BLM+agomiR-Merge.tif]

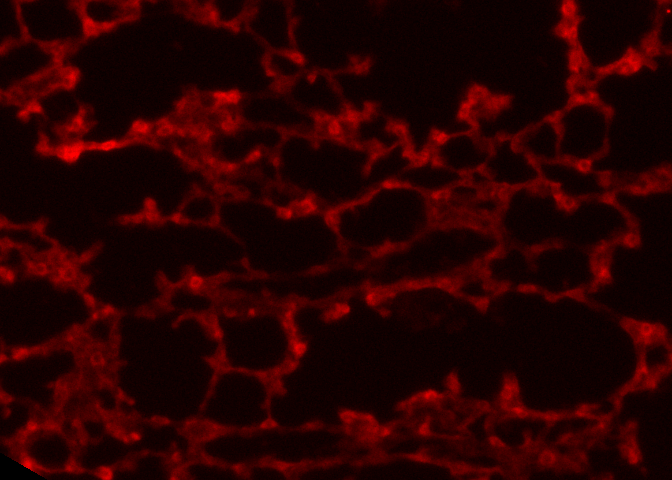

Supplement: Supplementary file 1 [file DataSheet1.ZIP › Supplementary materials/Original source data/uncropped images/Fig.3/Fig.3D BLM+agomiR-miR-130a-3p.tif]

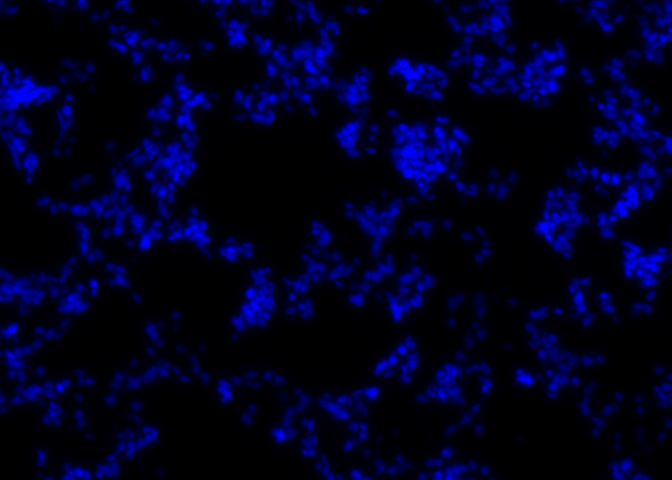

Supplement: Supplementary file 1 [file DataSheet1.ZIP › Supplementary materials/Original source data/uncropped images/Fig.3/Fig.3D BLM-DAPI.tif]

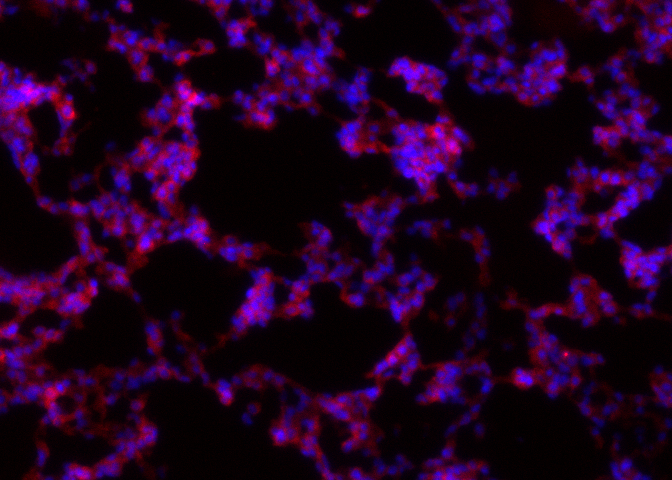

Supplement: Supplementary file 1 [file DataSheet1.ZIP › Supplementary materials/Original source data/uncropped images/Fig.3/Fig.3D BLM-Merge.tif]

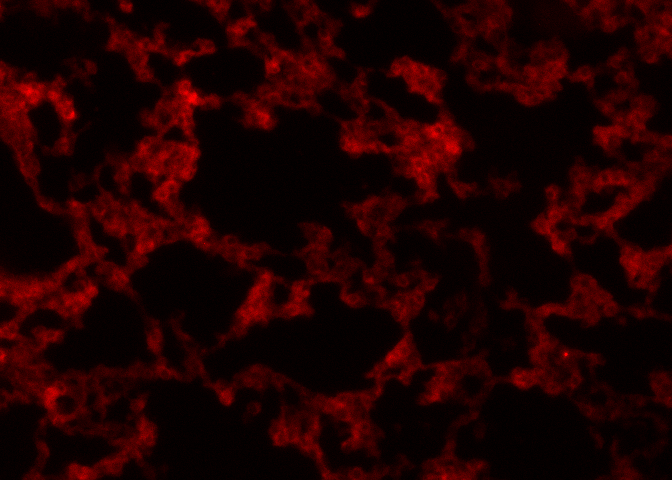

Supplement: Supplementary file 1 [file DataSheet1.ZIP › Supplementary materials/Original source data/uncropped images/Fig.3/Fig.3D BLM-miR-130a-3p.tif]

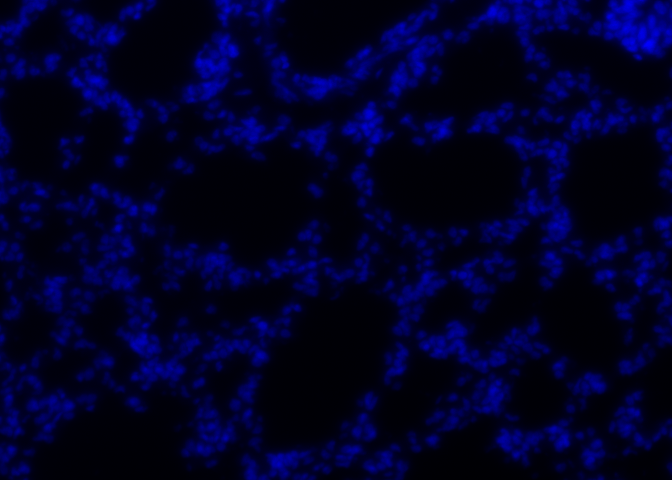

Supplement: Supplementary file 1 [file DataSheet1.ZIP › Supplementary materials/Original source data/uncropped images/Fig.3/Fig.3D Control-DAPI.tif]

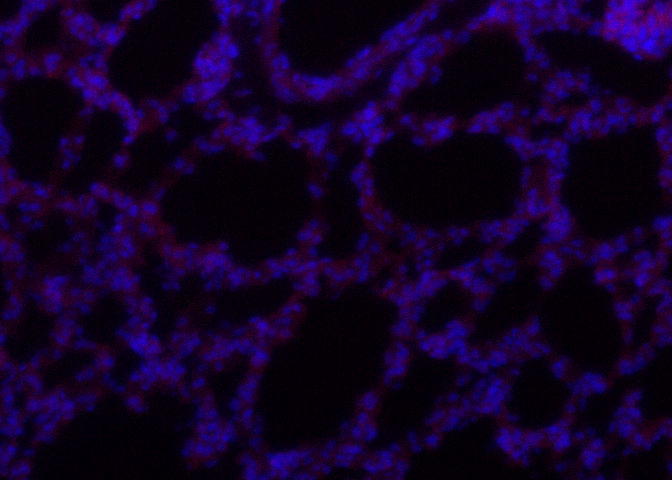

Supplement: Supplementary file 1 [file DataSheet1.ZIP › Supplementary materials/Original source data/uncropped images/Fig.3/Fig.3D Control-Merge.tif]

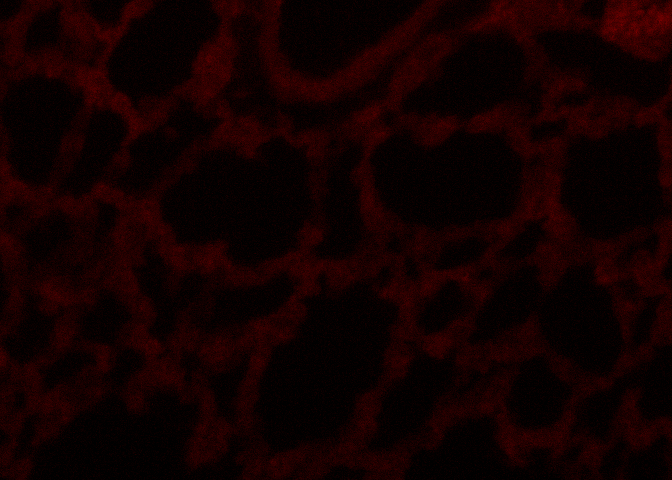

Supplement: Supplementary file 1 [file DataSheet1.ZIP › Supplementary materials/Original source data/uncropped images/Fig.3/Fig.3D Control-miR-130a-3p.tif]

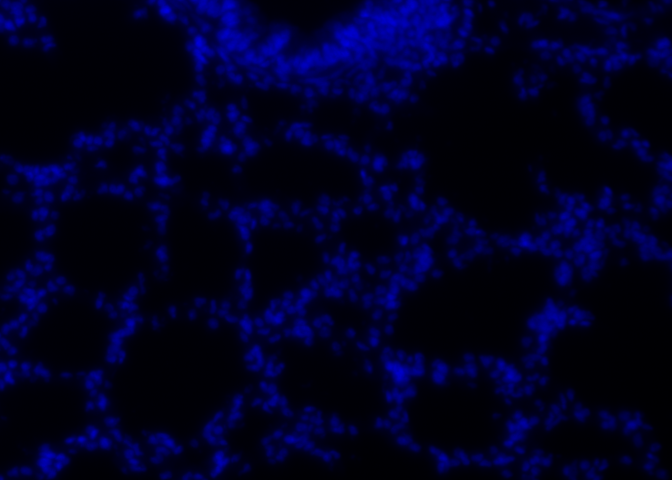

Supplement: Supplementary file 1 [file DataSheet1.ZIP › Supplementary materials/Original source data/uncropped images/Fig.3/Fig.3E agomiR-DAPI.tif]

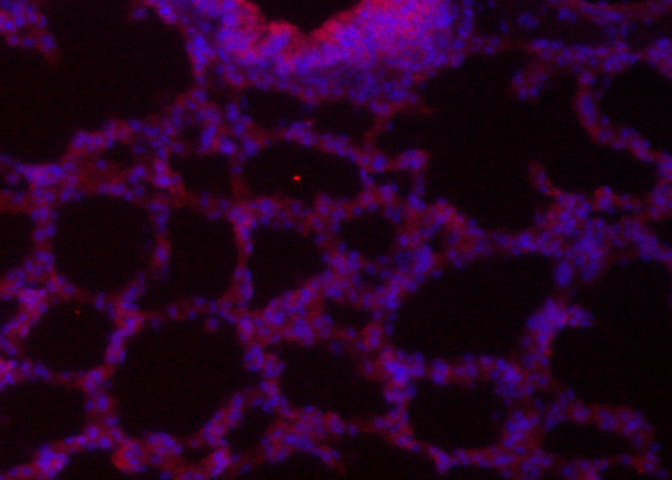

Supplement: Supplementary file 1 [file DataSheet1.ZIP › Supplementary materials/Original source data/uncropped images/Fig.3/Fig.3E agomiR-Merge.tif]

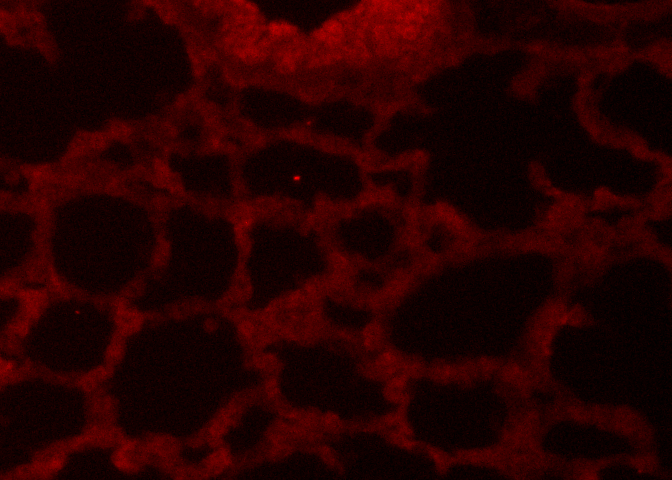

Supplement: Supplementary file 1 [file DataSheet1.ZIP › Supplementary materials/Original source data/uncropped images/Fig.3/Fig.3E agomiR-miR-130a-3p.tif]

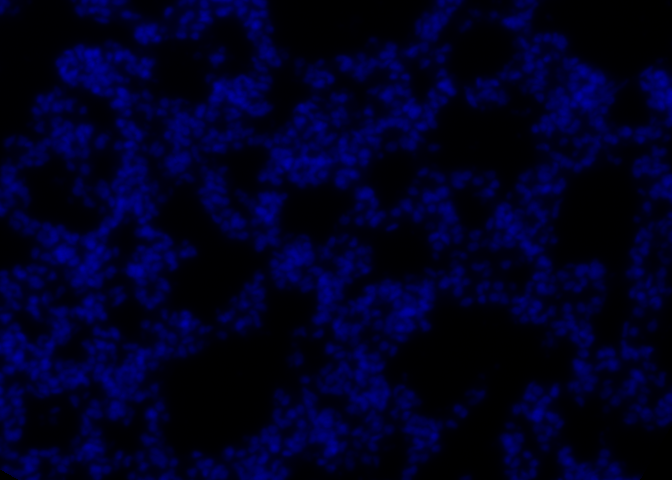

Supplement: Supplementary file 1 [file DataSheet1.ZIP › Supplementary materials/Original source data/uncropped images/Fig.3/Fig.3E BLM+agomiR-DAPI.tif]

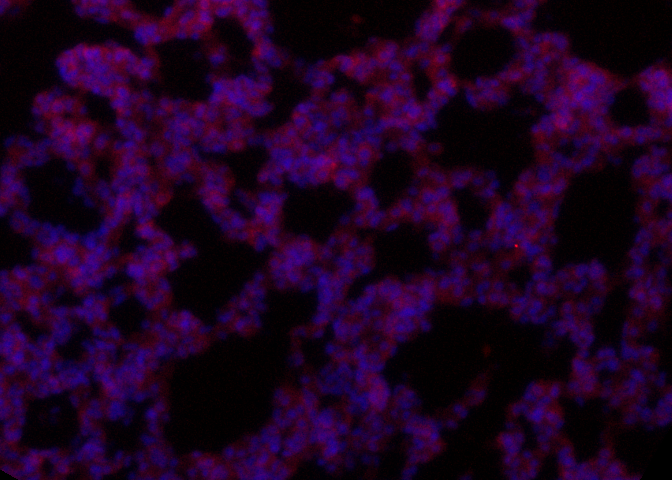

Supplement: Supplementary file 1 [file DataSheet1.ZIP › Supplementary materials/Original source data/uncropped images/Fig.3/Fig.3E BLM+agomiR-Merge.tif]

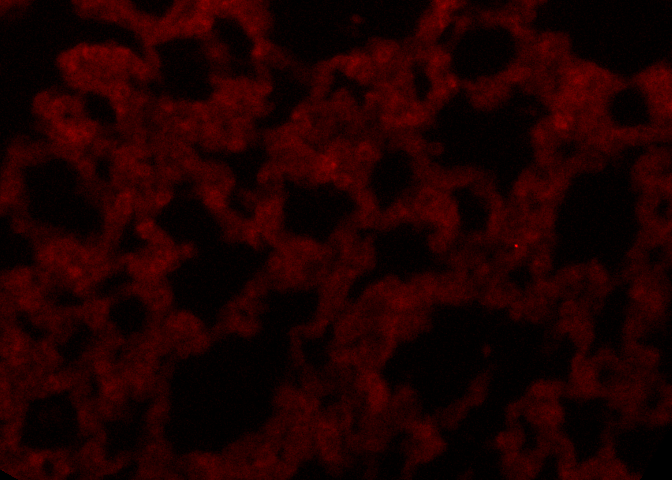

Supplement: Supplementary file 1 [file DataSheet1.ZIP › Supplementary materials/Original source data/uncropped images/Fig.3/Fig.3E BLM+agomiR-miR-130a-3p.tif]

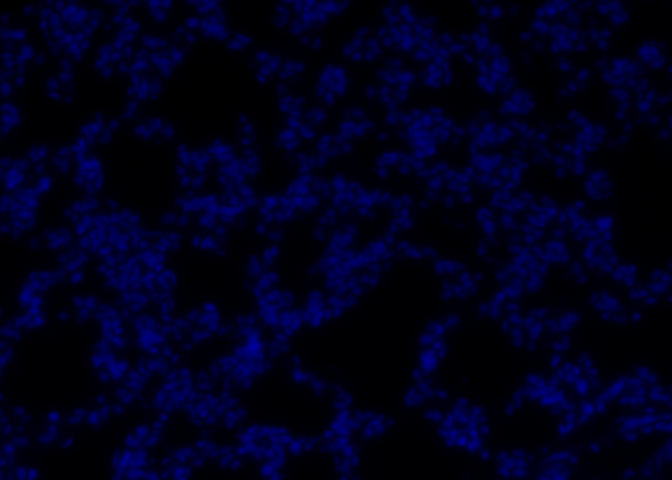

Supplement: Supplementary file 1 [file DataSheet1.ZIP › Supplementary materials/Original source data/uncropped images/Fig.3/Fig.3E BLM-DAPI.tif]

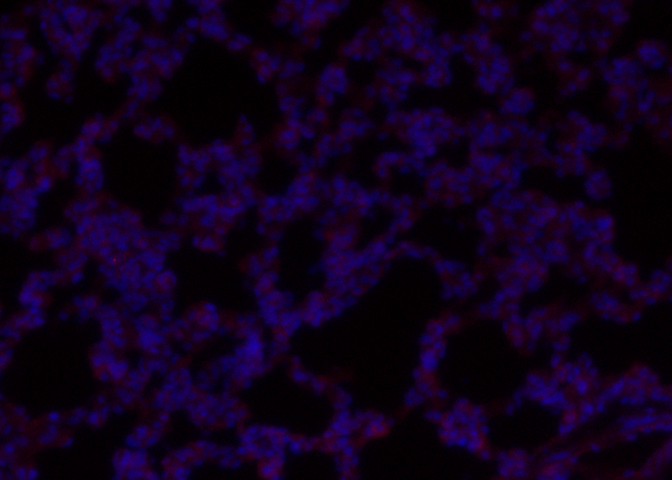

Supplement: Supplementary file 1 [file DataSheet1.ZIP › Supplementary materials/Original source data/uncropped images/Fig.3/Fig.3E BLM-Merge.tif]

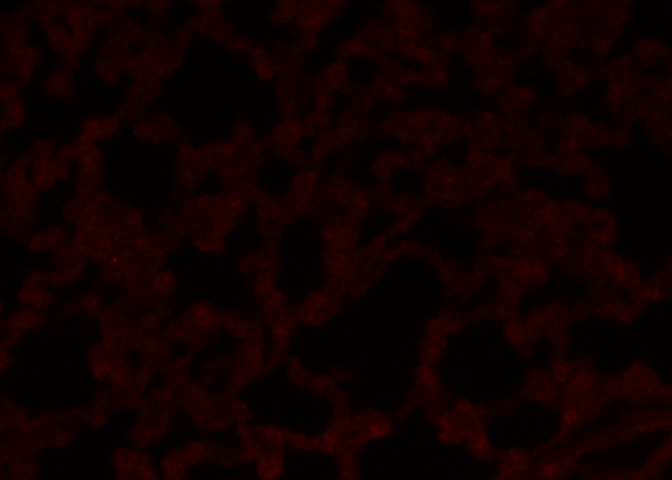

Supplement: Supplementary file 1 [file DataSheet1.ZIP › Supplementary materials/Original source data/uncropped images/Fig.3/Fig.3E BLM-miR-130a-3p.tif]

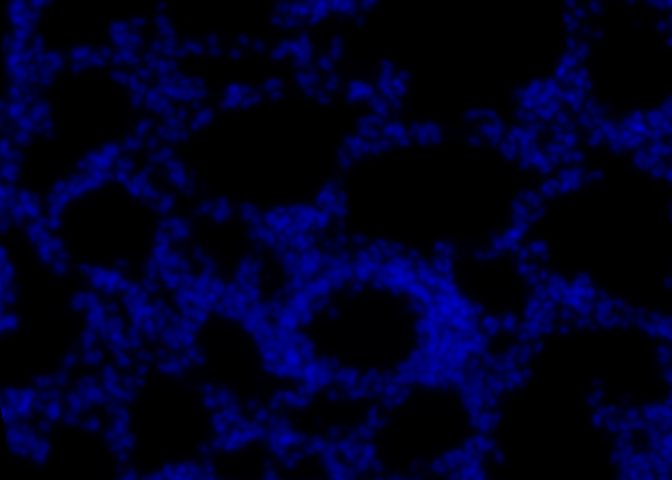

Supplement: Supplementary file 1 [file DataSheet1.ZIP › Supplementary materials/Original source data/uncropped images/Fig.3/Fig.3E Control-DAPI.tif]

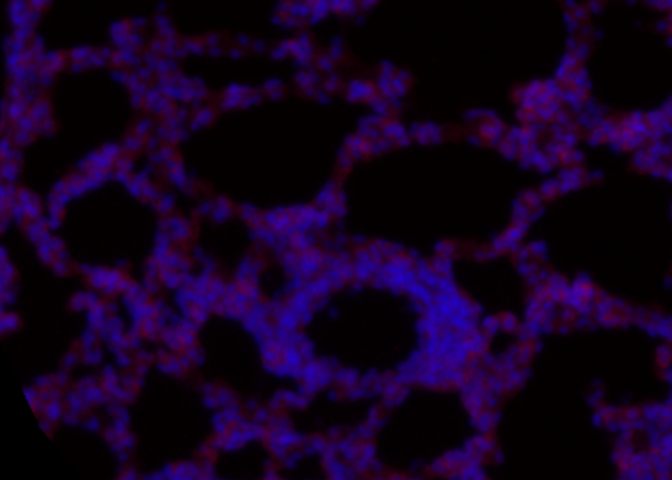

Supplement: Supplementary file 1 [file DataSheet1.ZIP › Supplementary materials/Original source data/uncropped images/Fig.3/Fig.3E Control-Merge.tif]

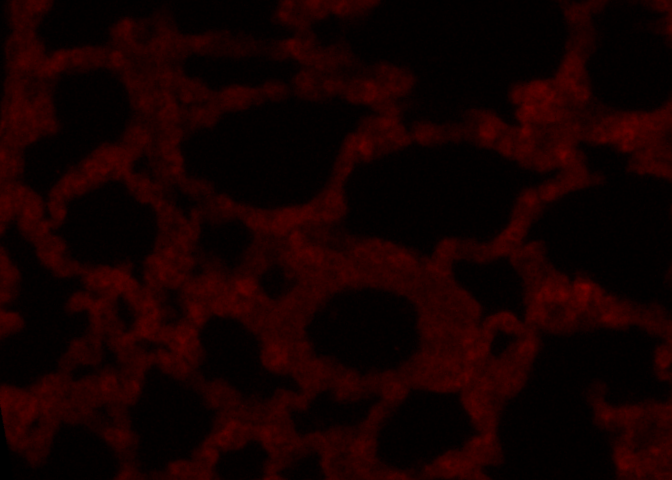

Supplement: Supplementary file 1 [file DataSheet1.ZIP › Supplementary materials/Original source data/uncropped images/Fig.3/Fig.3E Control-miR-130a-3p.tif]

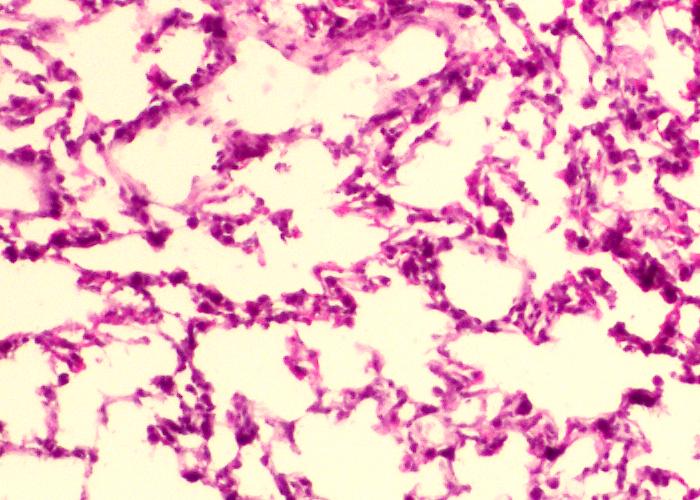

Supplement: Supplementary file 1 [file DataSheet1.ZIP › Supplementary materials/Original source data/uncropped images/Fig.5/Fig.5A HE-agomiR.tiff]

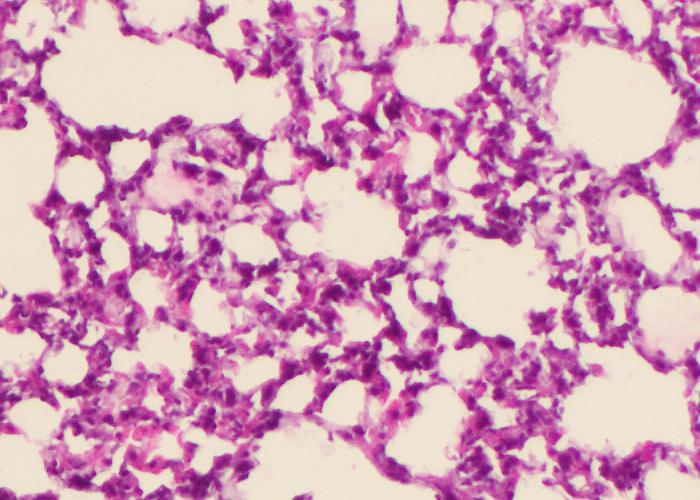

Supplement: Supplementary file 1 [file DataSheet1.ZIP › Supplementary materials/Original source data/uncropped images/Fig.5/Fig.5A HE-agomiR+BLM.tiff]

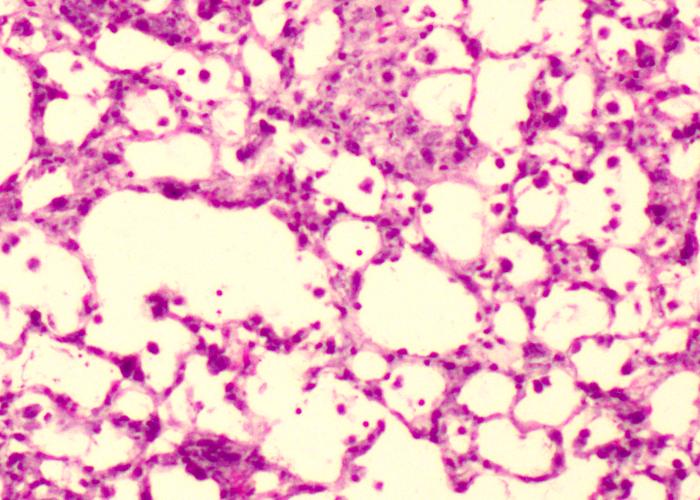

Supplement: Supplementary file 1 [file DataSheet1.ZIP › Supplementary materials/Original source data/uncropped images/Fig.5/Fig.5A HE-BLM.tiff]

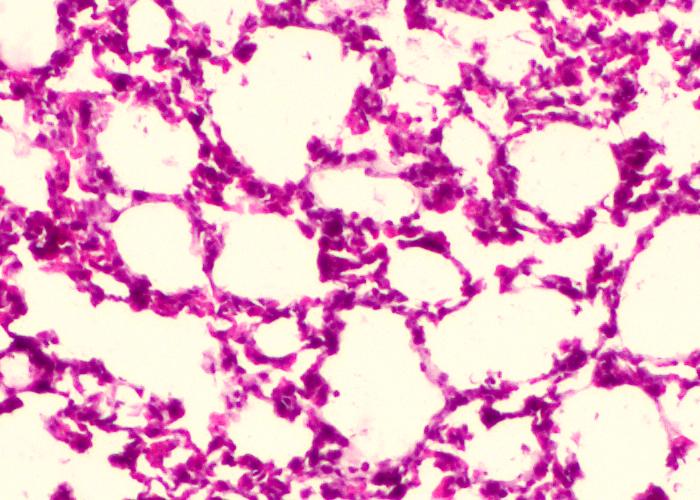

Supplement: Supplementary file 1 [file DataSheet1.ZIP › Supplementary materials/Original source data/uncropped images/Fig.5/Fig.5A HE-Control.tiff]

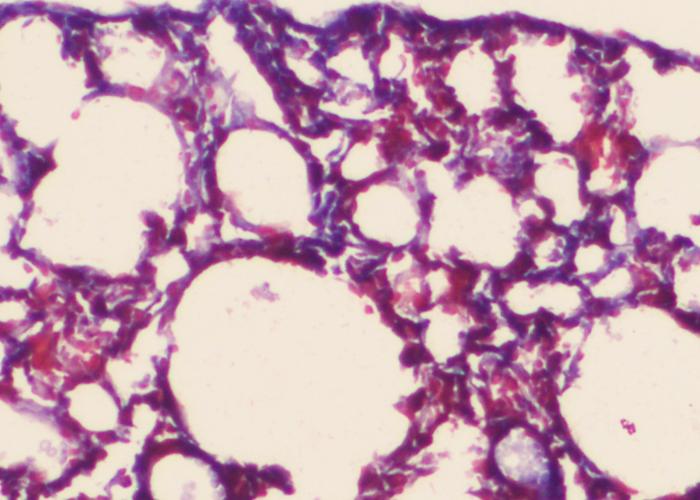

Supplement: Supplementary file 1 [file DataSheet1.ZIP › Supplementary materials/Original source data/uncropped images/Fig.5/Fig.5A Masson-agomiR.tiff]

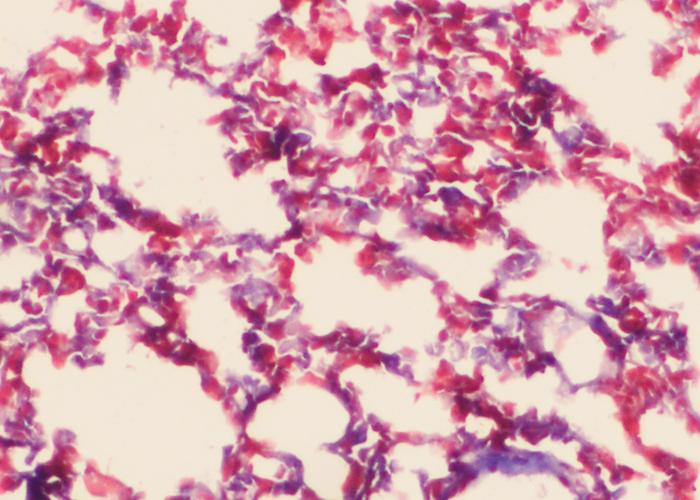

Supplement: Supplementary file 1 [file DataSheet1.ZIP › Supplementary materials/Original source data/uncropped images/Fig.5/Fig.5A Masson-agomiR+BLM.tiff]

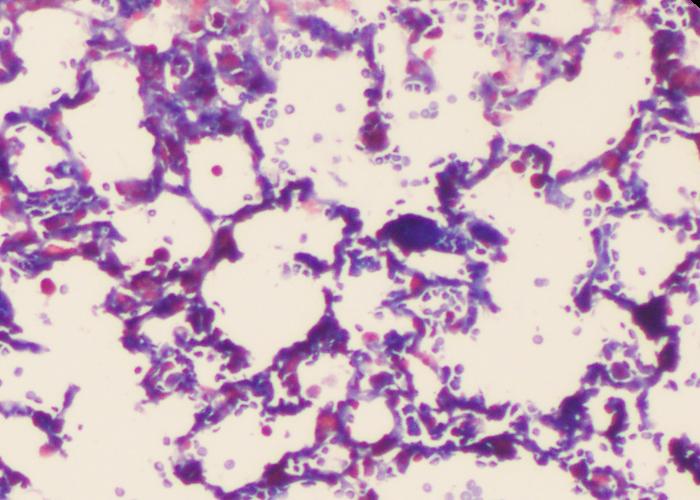

Supplement: Supplementary file 1 [file DataSheet1.ZIP › Supplementary materials/Original source data/uncropped images/Fig.5/Fig.5A Masson-BLM.tiff]

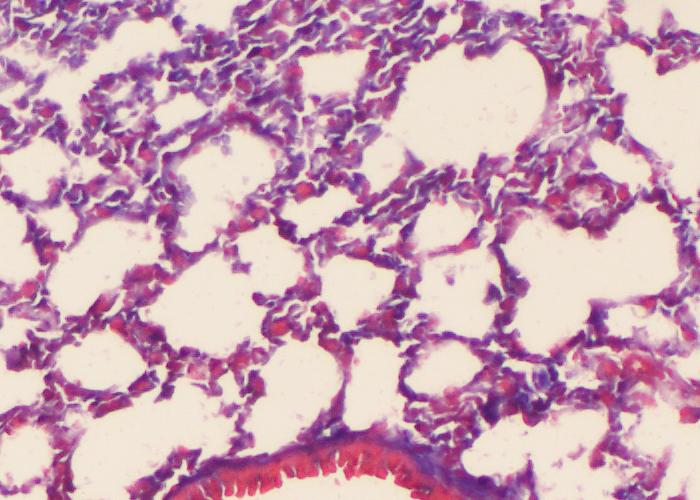

Supplement: Supplementary file 1 [file DataSheet1.ZIP › Supplementary materials/Original source data/uncropped images/Fig.5/Fig.5A Masson-Control.tiff]

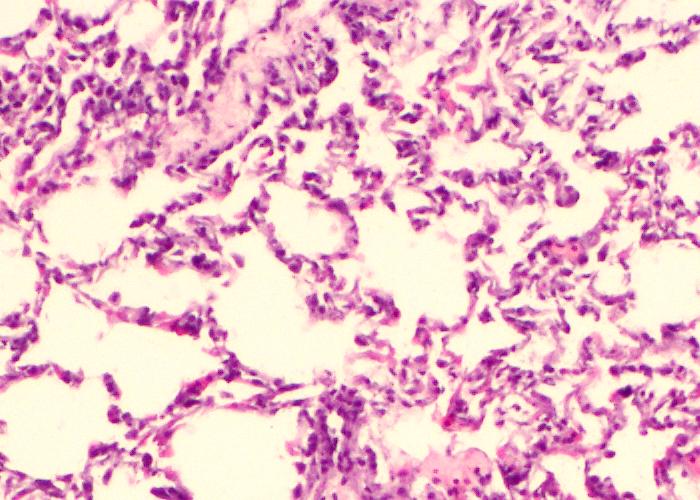

Supplement: Supplementary file 1 [file DataSheet1.ZIP › Supplementary materials/Original source data/uncropped images/Fig.5/Fig.5B HE-agomiR.tiff]

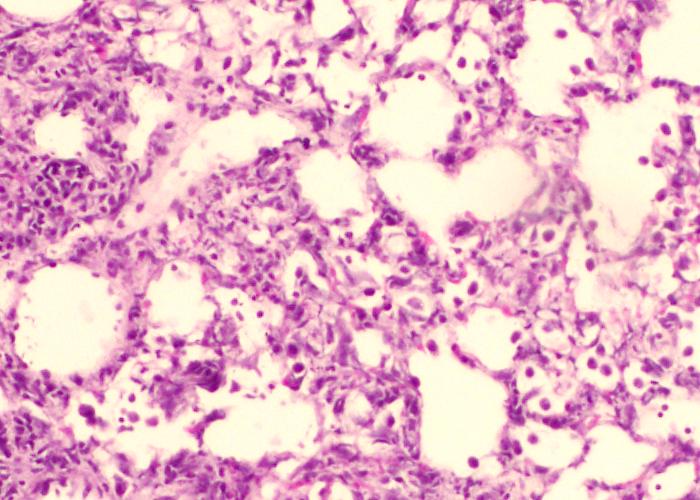

Supplement: Supplementary file 1 [file DataSheet1.ZIP › Supplementary materials/Original source data/uncropped images/Fig.5/Fig.5B HE-agomiR+BLM.tiff]

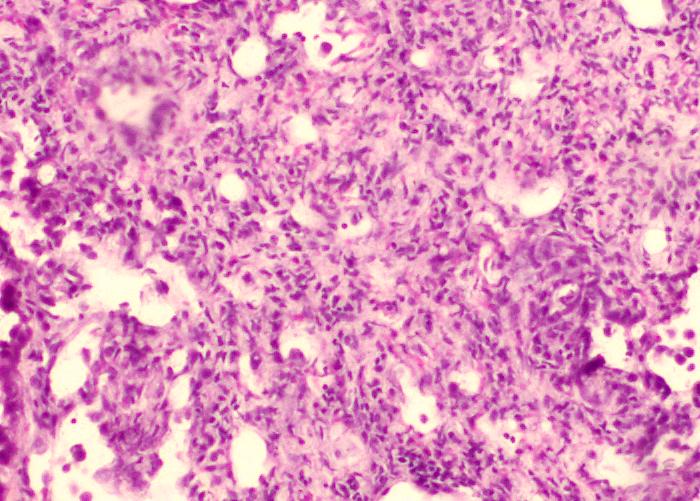

Supplement: Supplementary file 1 [file DataSheet1.ZIP › Supplementary materials/Original source data/uncropped images/Fig.5/Fig.5B HE-BLM.tiff]

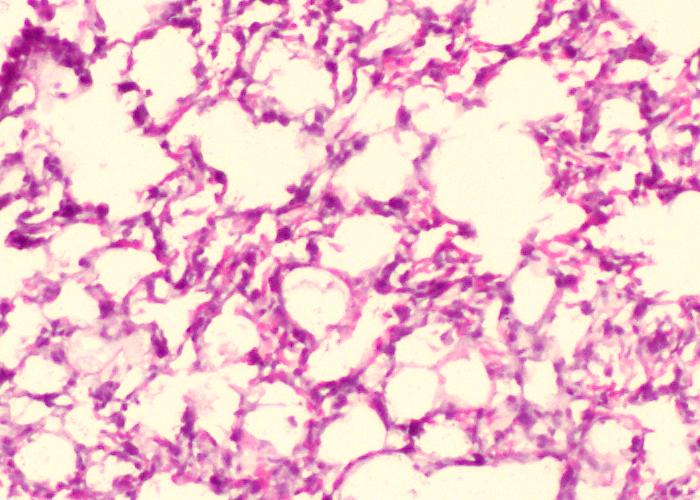

Supplement: Supplementary file 1 [file DataSheet1.ZIP › Supplementary materials/Original source data/uncropped images/Fig.5/Fig.5B HE-Control.tiff]

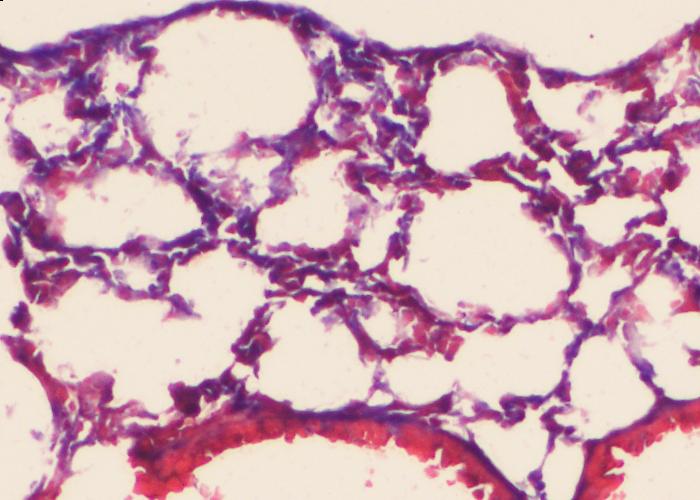

Supplement: Supplementary file 1 [file DataSheet1.ZIP › Supplementary materials/Original source data/uncropped images/Fig.5/Fig.5B Masson-agomiR.tiff]

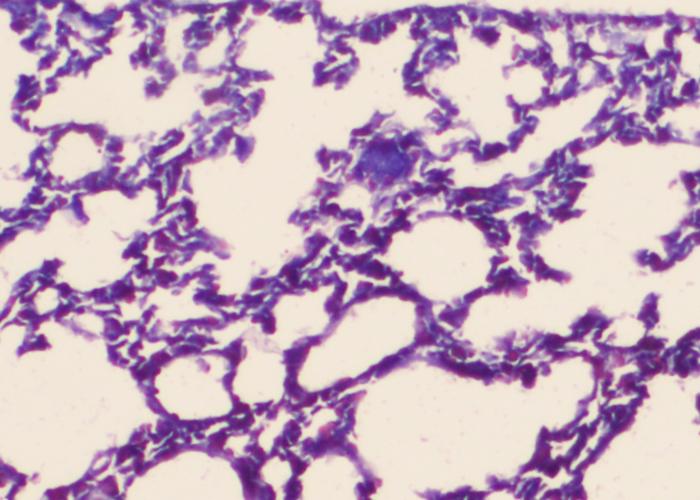

Supplement: Supplementary file 1 [file DataSheet1.ZIP › Supplementary materials/Original source data/uncropped images/Fig.5/Fig.5B Masson-agomiR+BLM.tiff]

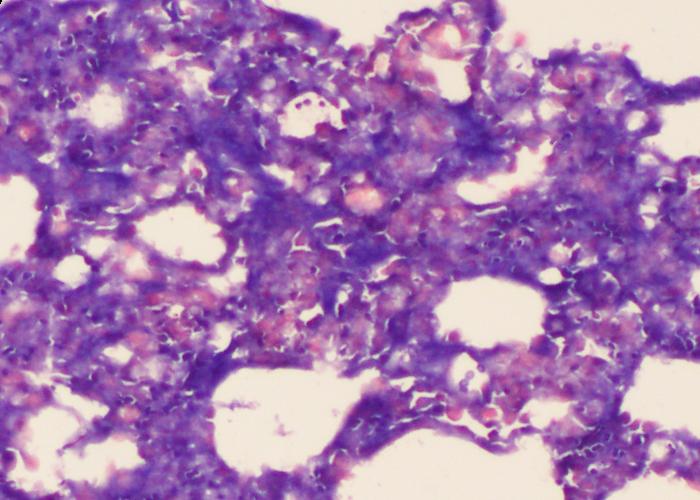

Supplement: Supplementary file 1 [file DataSheet1.ZIP › Supplementary materials/Original source data/uncropped images/Fig.5/Fig.5B Masson-BLM.tiff]

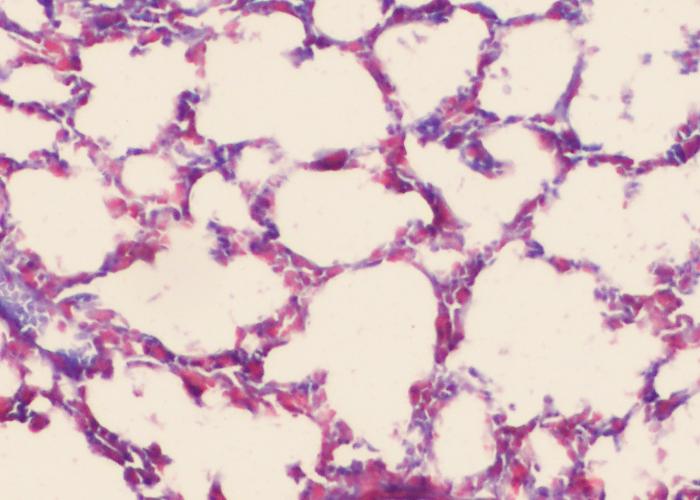

Supplement: Supplementary file 1 [file DataSheet1.ZIP › Supplementary materials/Original source data/uncropped images/Fig.5/Fig.5B Masson-Control.tiff]

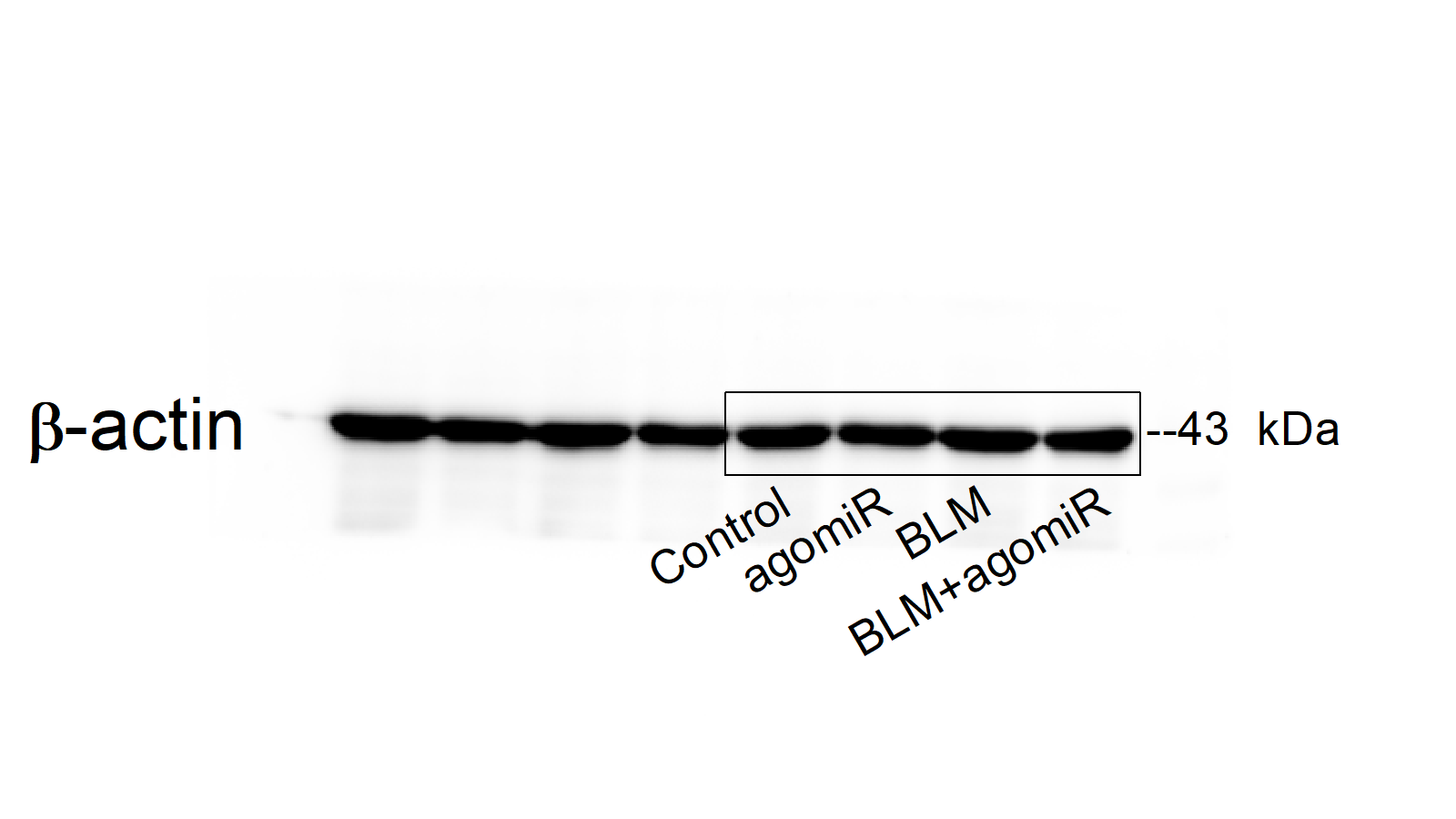

Supplement: Supplementary file 1 [file DataSheet1.ZIP › Supplementary materials/Original source data/uncropped images/Fig.6/Fig.6C b-actin.tiff]

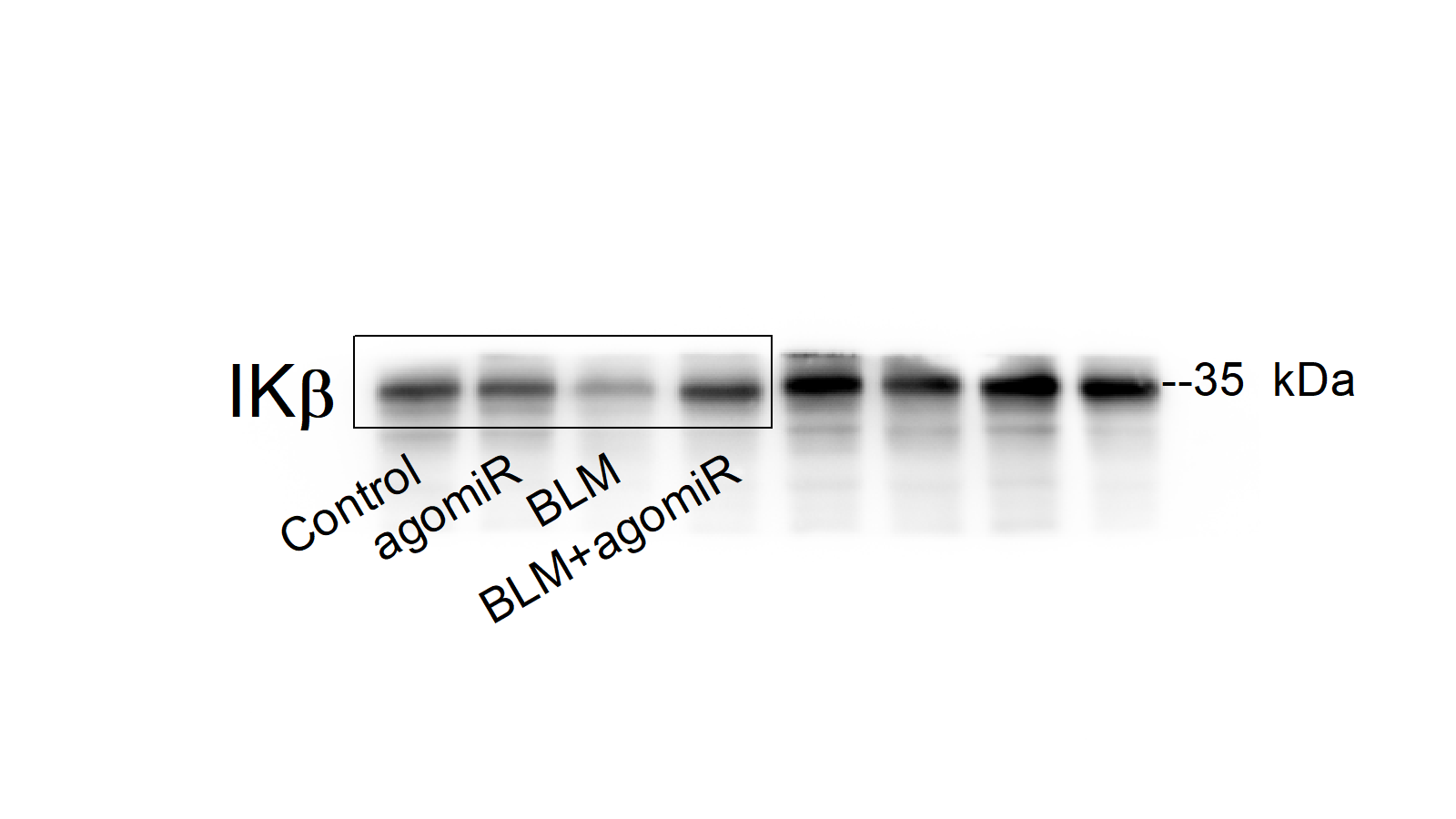

Supplement: Supplementary file 1 [file DataSheet1.ZIP › Supplementary materials/Original source data/uncropped images/Fig.6/Fig.6C IKb.tiff]

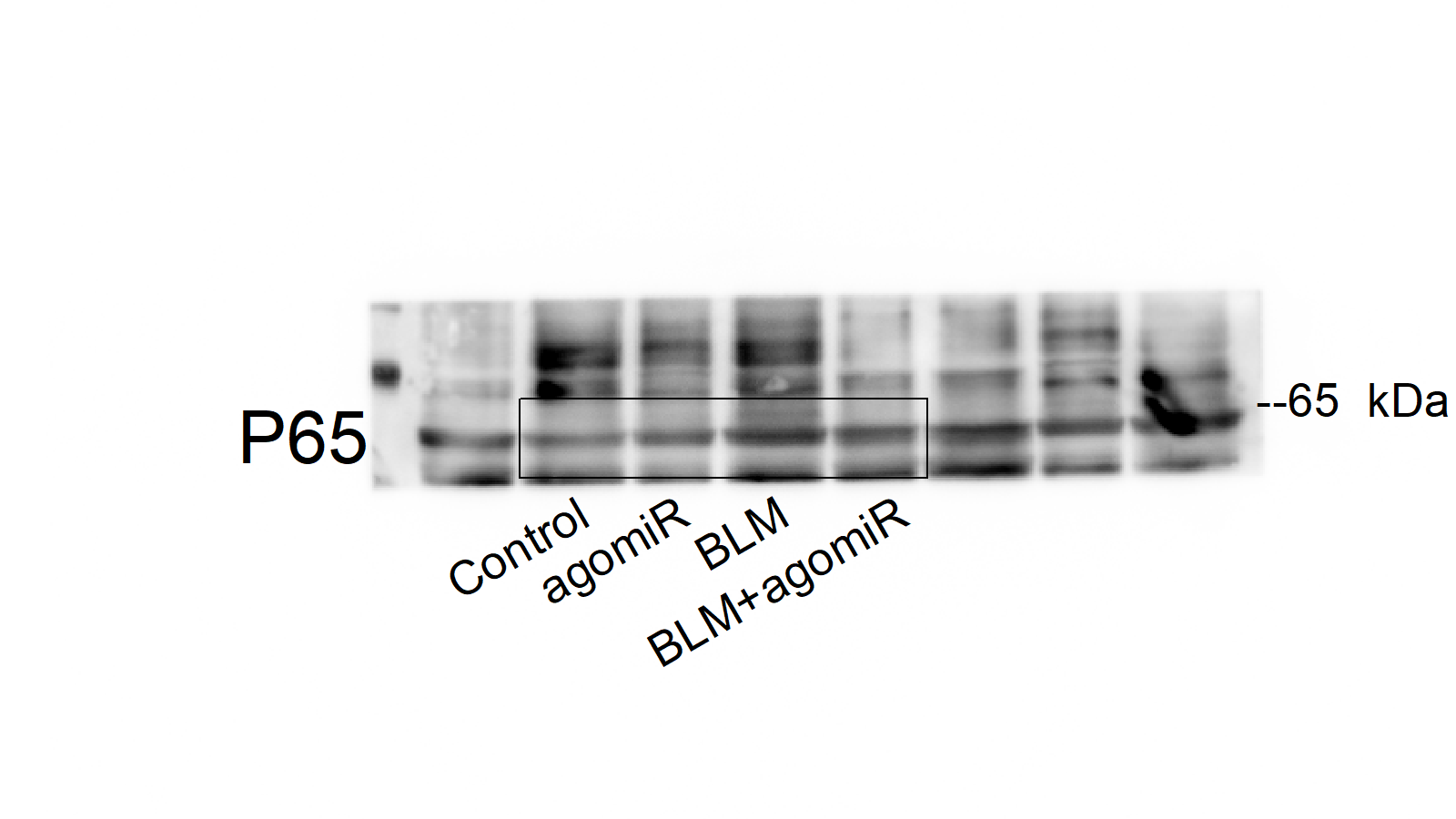

Supplement: Supplementary file 1 [file DataSheet1.ZIP › Supplementary materials/Original source data/uncropped images/Fig.6/Fig.6C P65.tiff]

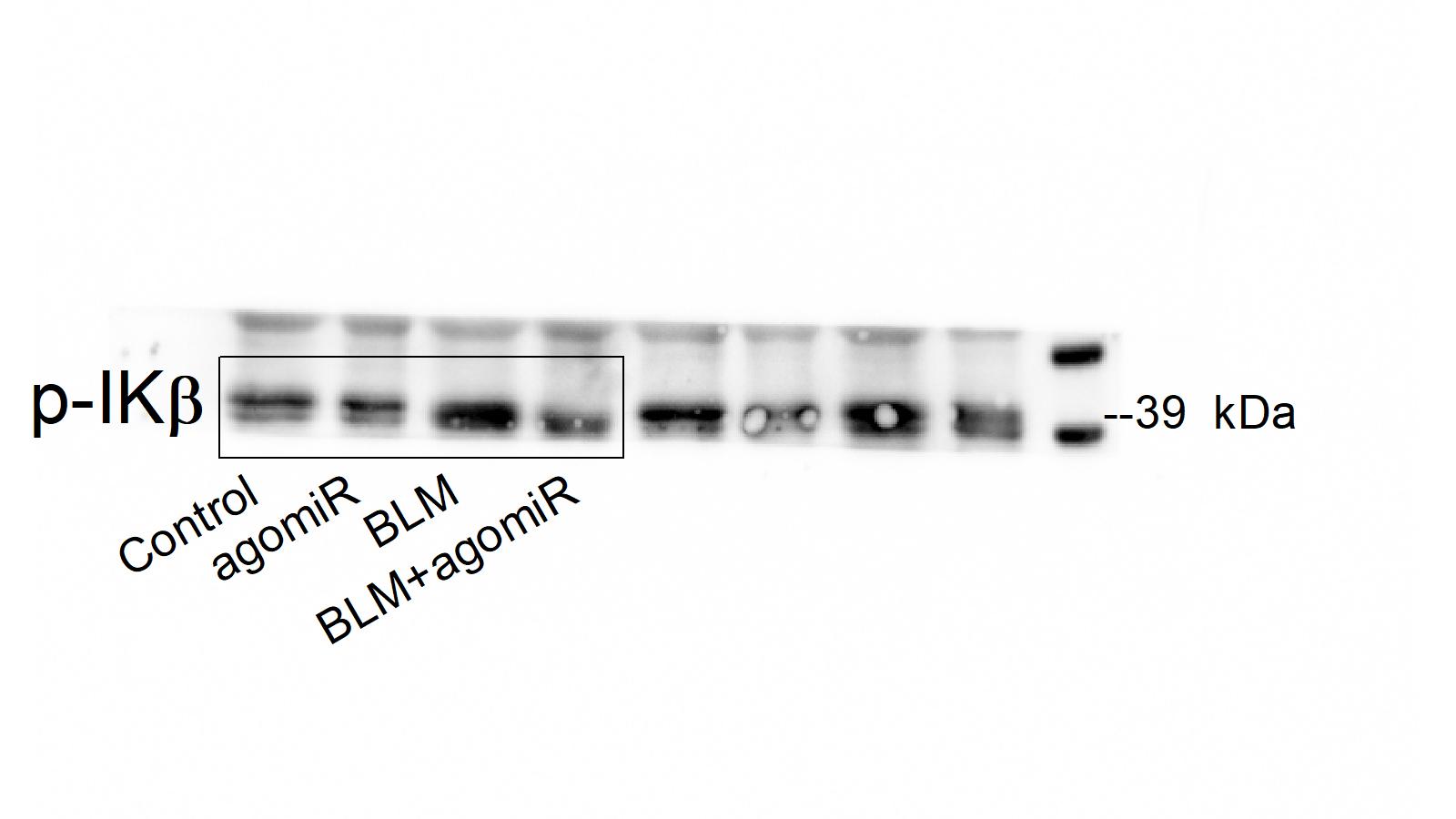

Supplement: Supplementary file 1 [file DataSheet1.ZIP › Supplementary materials/Original source data/uncropped images/Fig.6/Fig.6C p-IKb.tiff]

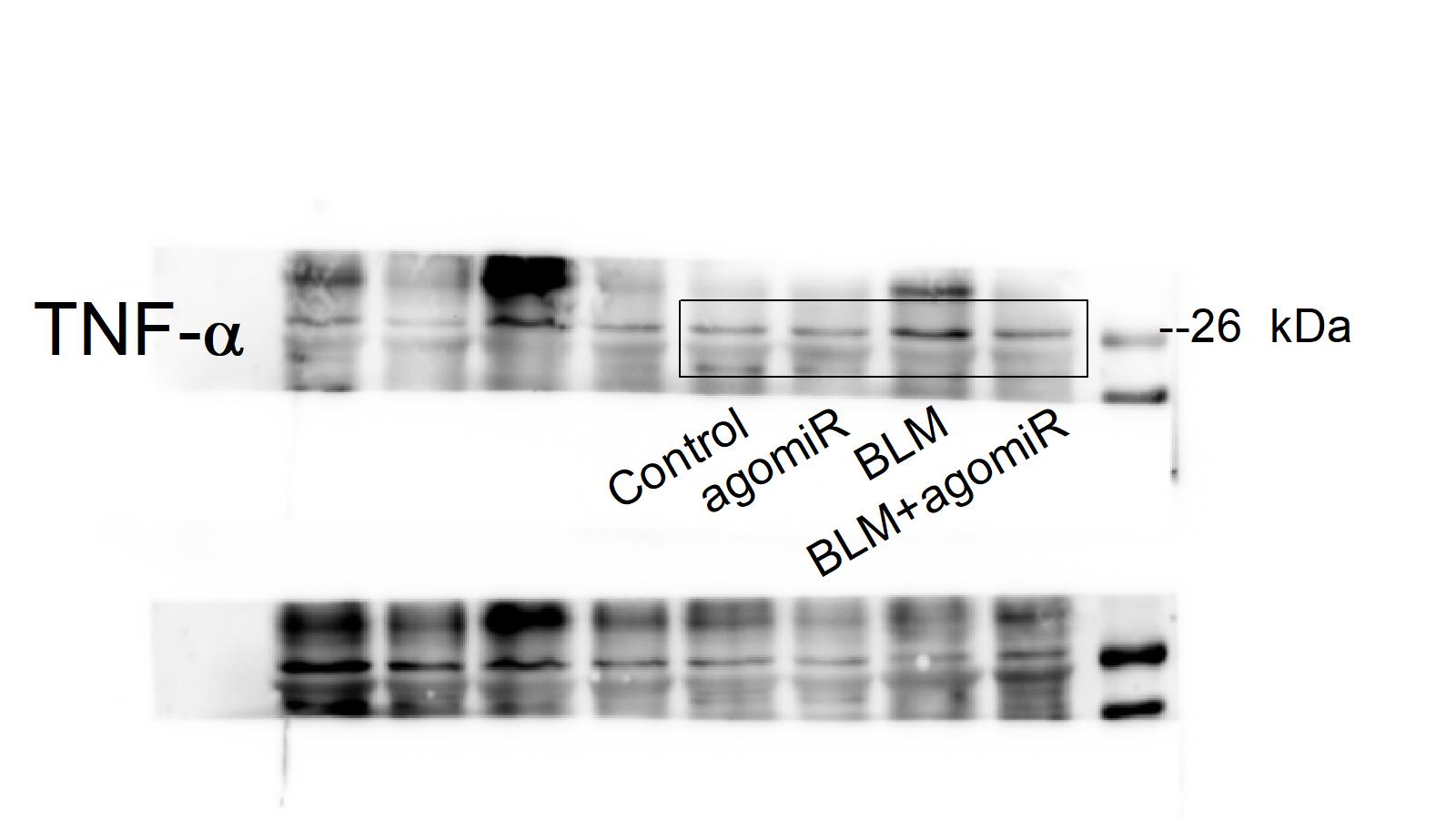

Supplement: Supplementary file 1 [file DataSheet1.ZIP › Supplementary materials/Original source data/uncropped images/Fig.6/Fig.6C TNF-a.tiff]

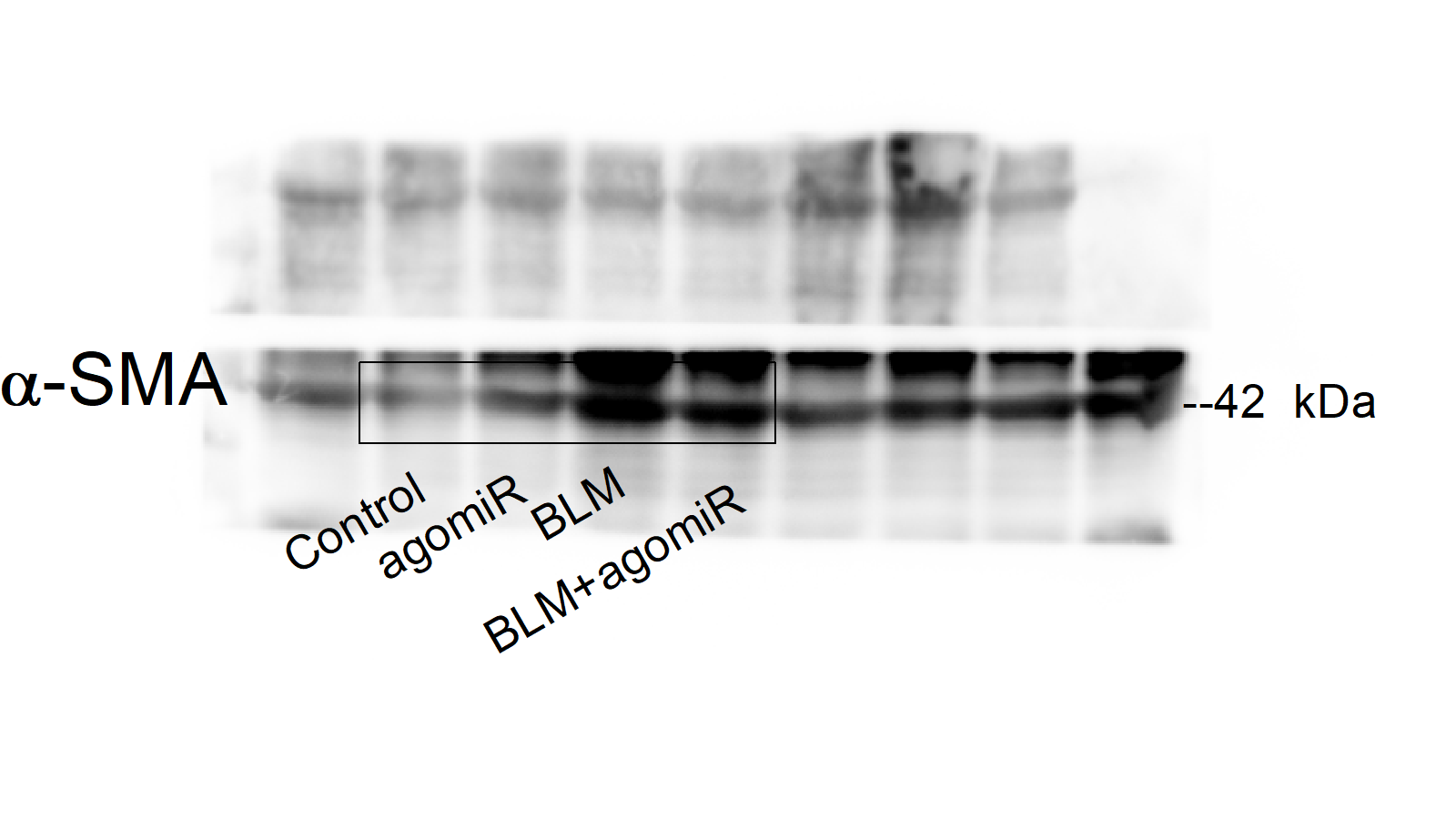

Supplement: Supplementary file 1 [file DataSheet1.ZIP › Supplementary materials/Original source data/uncropped images/Fig.6/Fig.6G a-SMA.tiff]

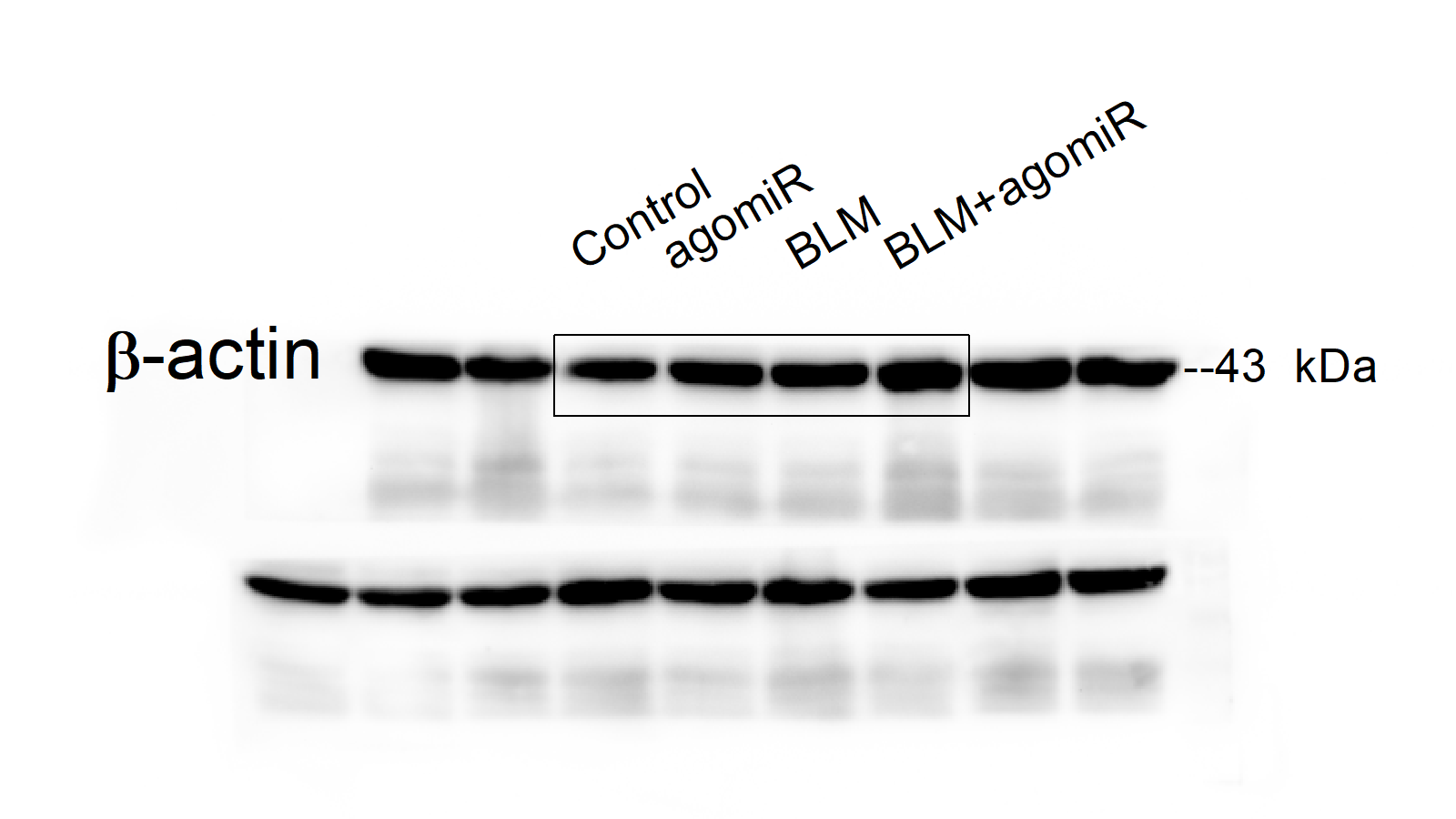

Supplement: Supplementary file 1 [file DataSheet1.ZIP › Supplementary materials/Original source data/uncropped images/Fig.6/Fig.6G b-actin.tiff]

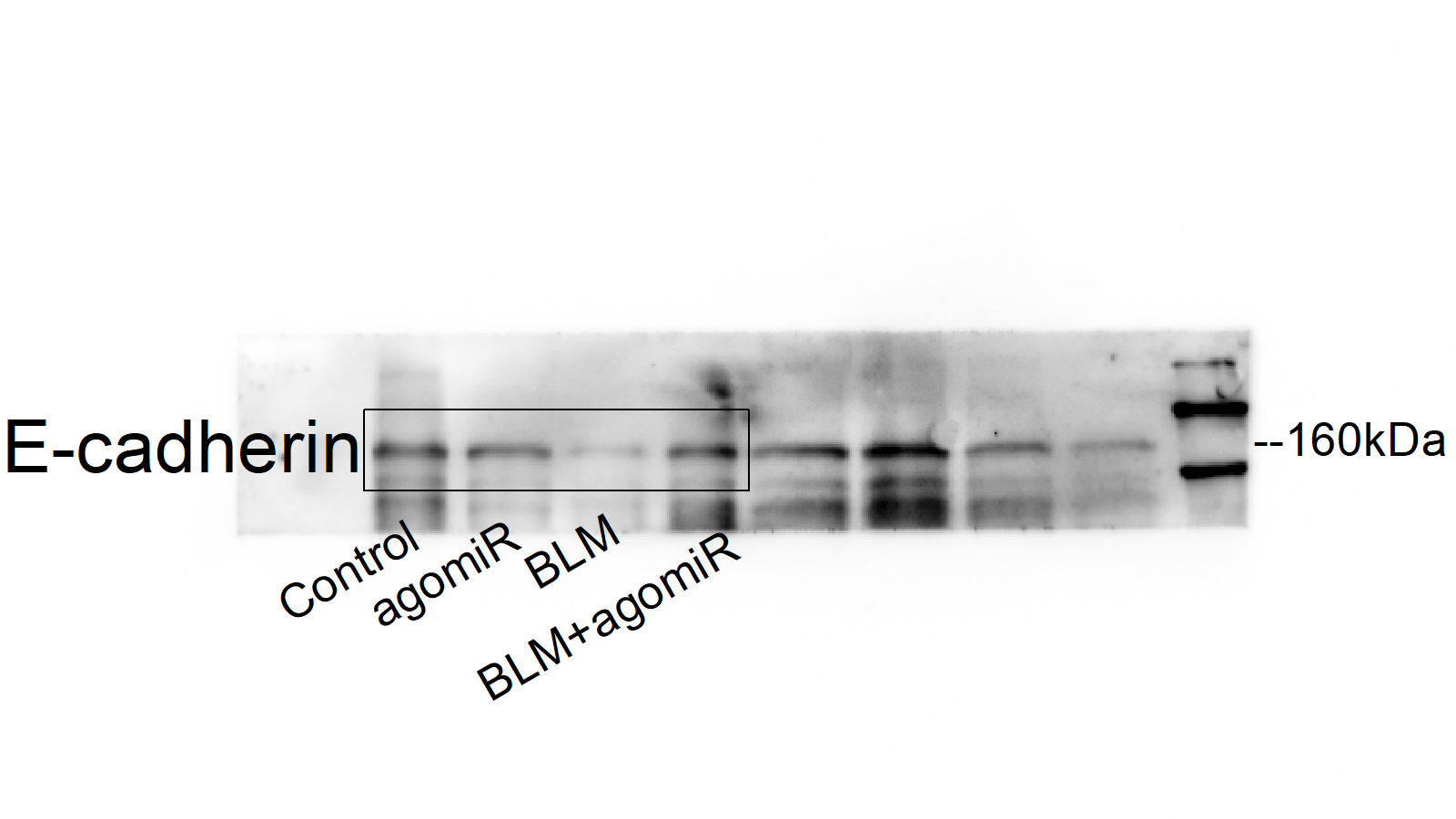

Supplement: Supplementary file 1 [file DataSheet1.ZIP › Supplementary materials/Original source data/uncropped images/Fig.6/Fig.6G E-cadherin.tiff]

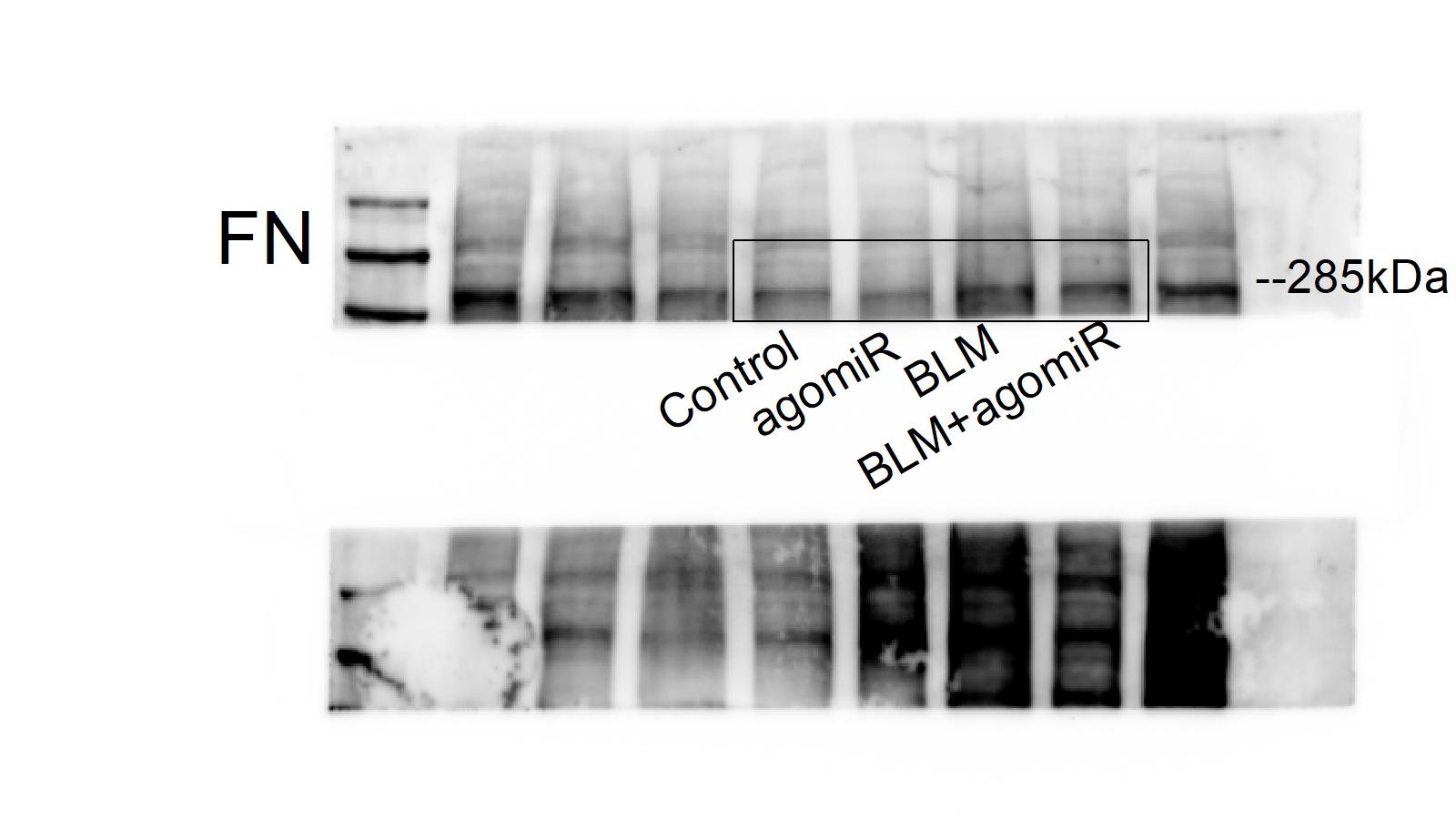

Supplement: Supplementary file 1 [file DataSheet1.ZIP › Supplementary materials/Original source data/uncropped images/Fig.6/Fig.6G FN.tiff]

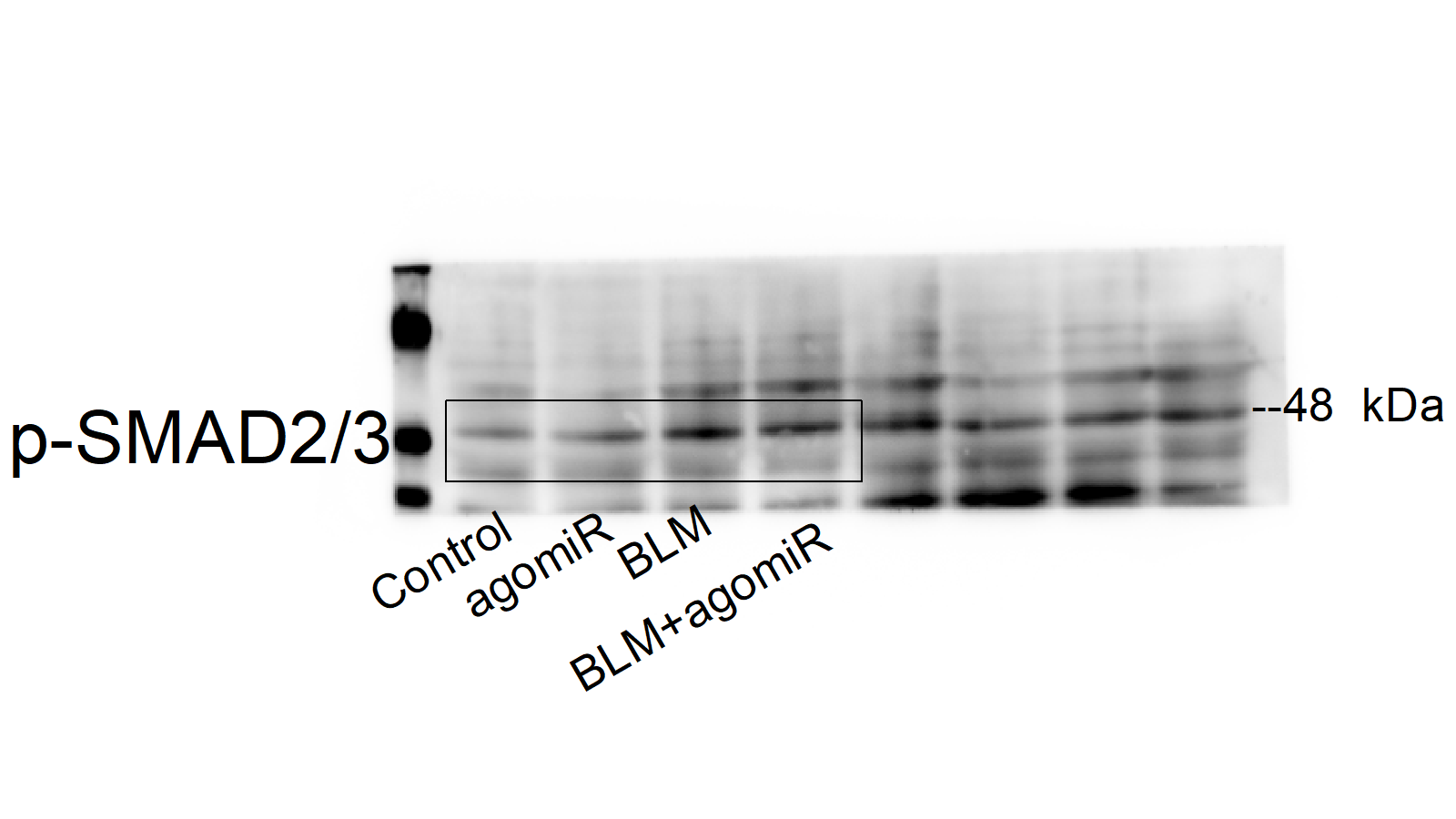

Supplement: Supplementary file 1 [file DataSheet1.ZIP › Supplementary materials/Original source data/uncropped images/Fig.6/Fig.6G p-SMAD23.tiff]

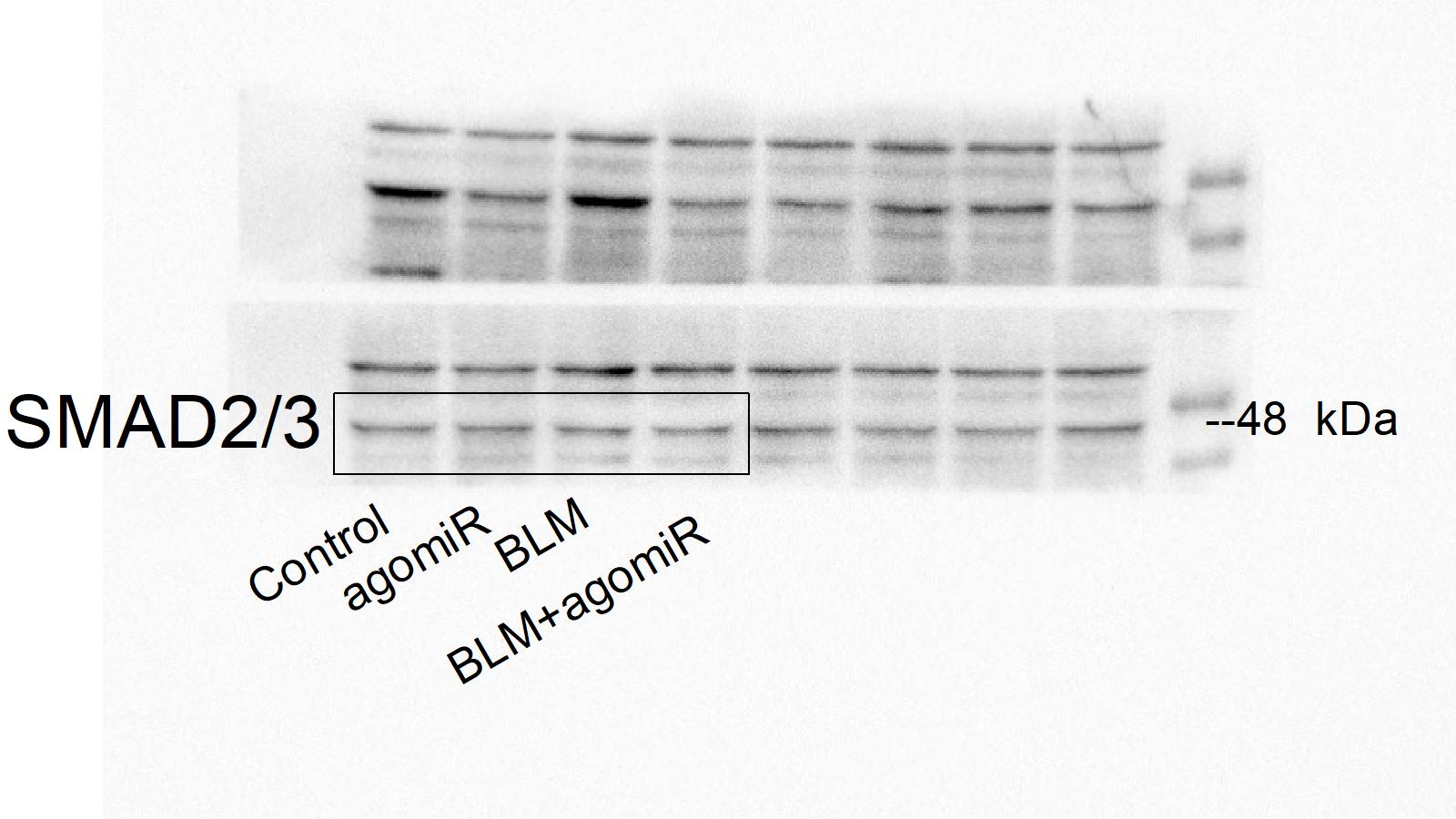

Supplement: Supplementary file 1 [file DataSheet1.ZIP › Supplementary materials/Original source data/uncropped images/Fig.6/Fig.6G SMAD23.tiff]

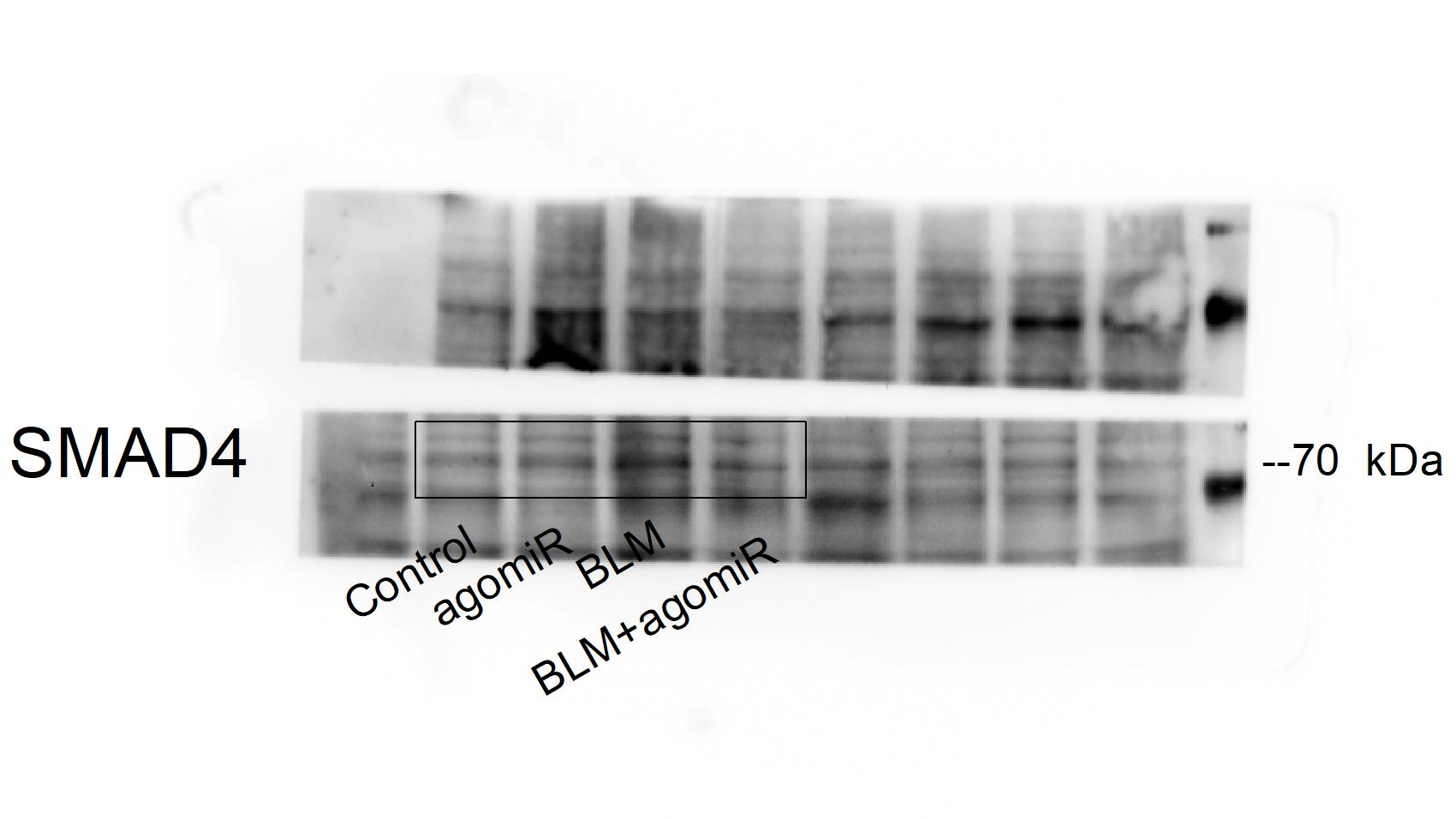

Supplement: Supplementary file 1 [file DataSheet1.ZIP › Supplementary materials/Original source data/uncropped images/Fig.6/Fig.6G SMAD4.tiff]

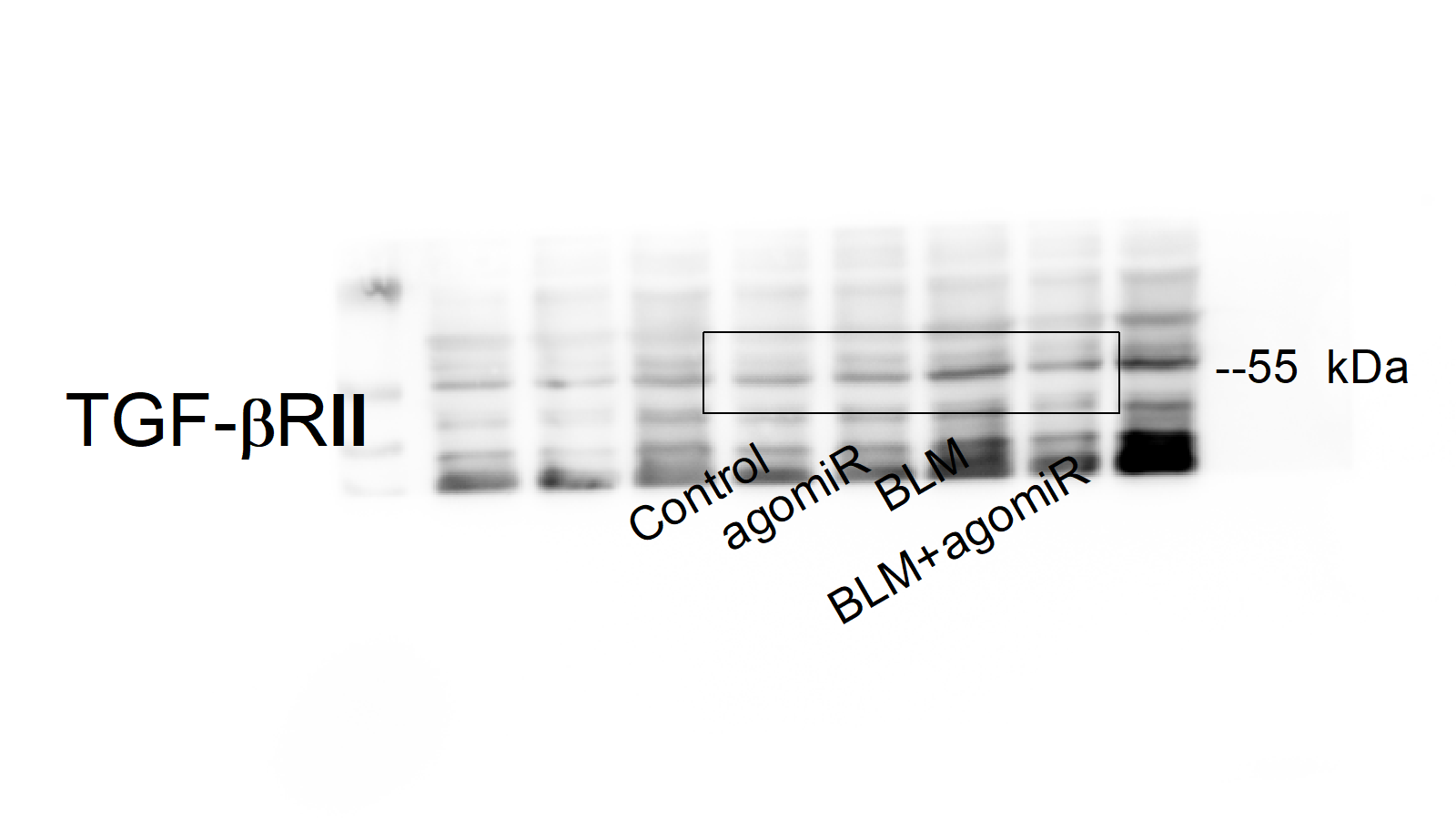

Supplement: Supplementary file 1 [file DataSheet1.ZIP › Supplementary materials/Original source data/uncropped images/Fig.6/Fig.6G TGF-bRII.tiff]

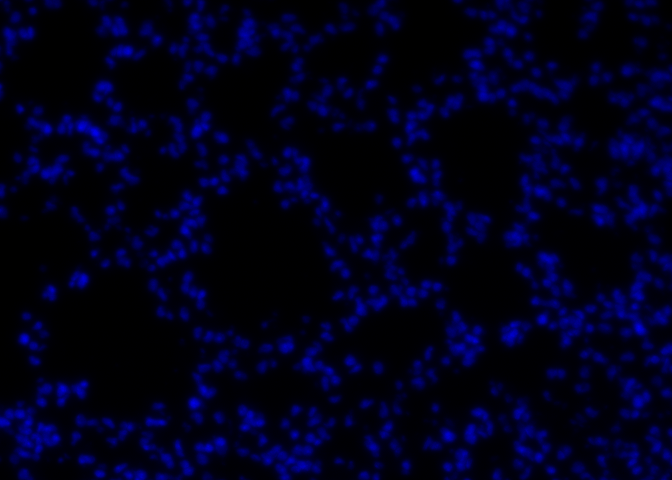

Supplement: Supplementary file 1 [file DataSheet1.ZIP › Supplementary materials/Original source data/uncropped images/Fig.6/Immunofluorescence/Fig.6I agomiR-DAPI.tif]

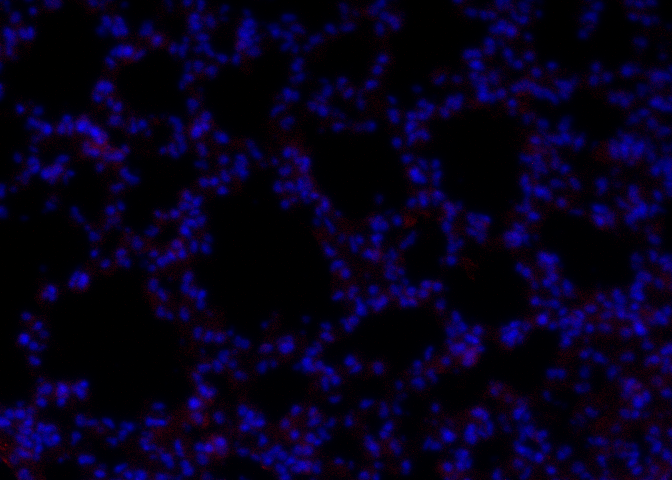

Supplement: Supplementary file 1 [file DataSheet1.ZIP › Supplementary materials/Original source data/uncropped images/Fig.6/Immunofluorescence/Fig.6I agomiR-Merge.tif]

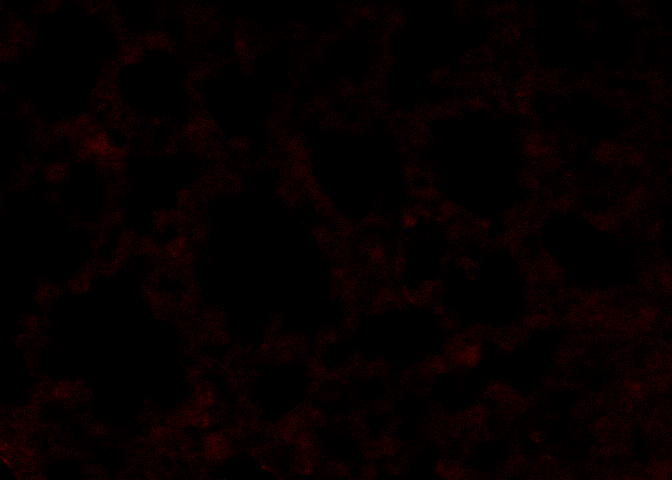

Supplement: Supplementary file 1 [file DataSheet1.ZIP › Supplementary materials/Original source data/uncropped images/Fig.6/Immunofluorescence/Fig.6I agomiR-miR-130a-3p.tif]

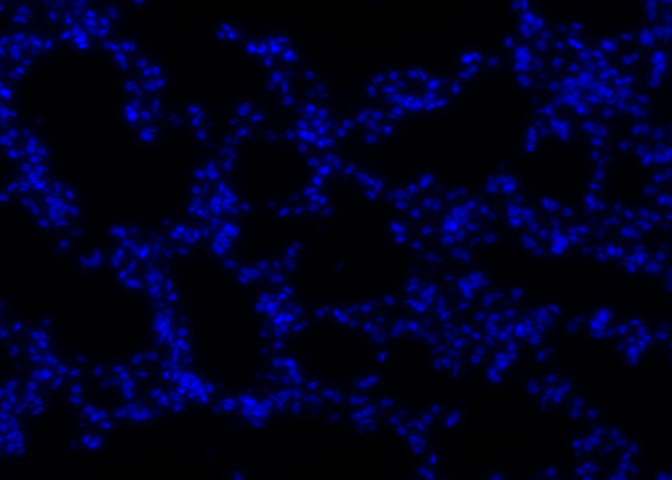

Supplement: Supplementary file 1 [file DataSheet1.ZIP › Supplementary materials/Original source data/uncropped images/Fig.6/Immunofluorescence/Fig.6I BLM+agomiR-DAPI.tif]

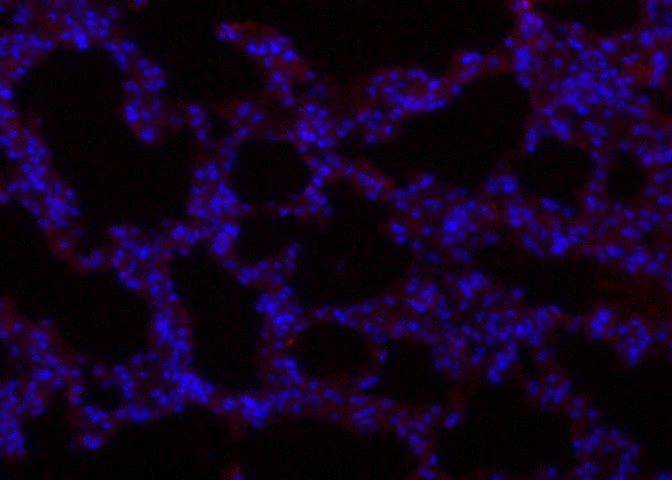

Supplement: Supplementary file 1 [file DataSheet1.ZIP › Supplementary materials/Original source data/uncropped images/Fig.6/Immunofluorescence/Fig.6I BLM+agomiR-Merge.tif]

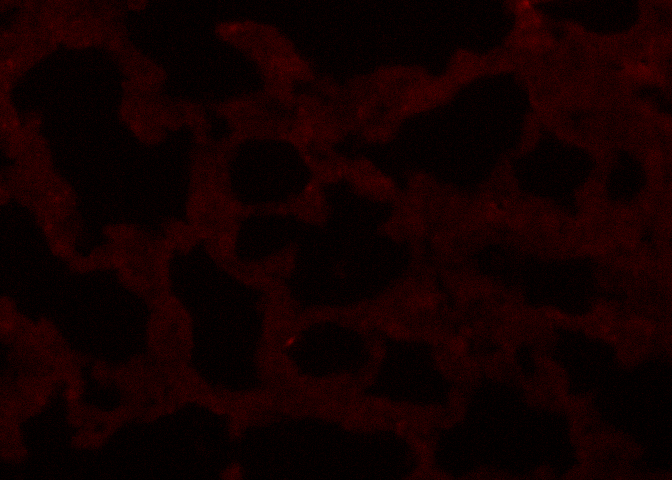

Supplement: Supplementary file 1 [file DataSheet1.ZIP › Supplementary materials/Original source data/uncropped images/Fig.6/Immunofluorescence/Fig.6I BLM+agomiR-miR-130a-3p.tif]

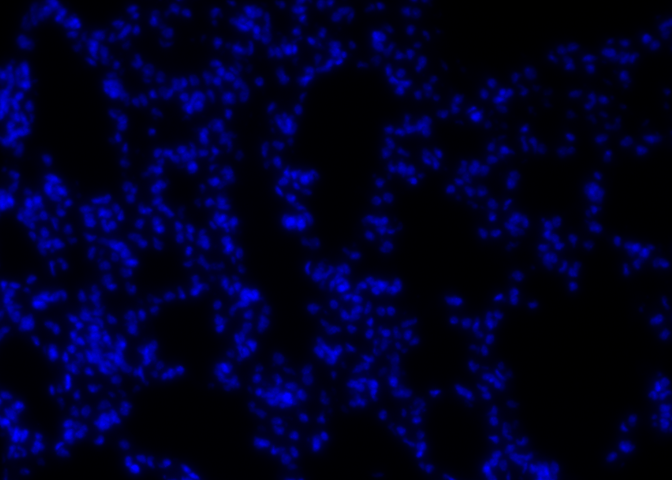

Supplement: Supplementary file 1 [file DataSheet1.ZIP › Supplementary materials/Original source data/uncropped images/Fig.6/Immunofluorescence/Fig.6I BLM-DAPI.tif]

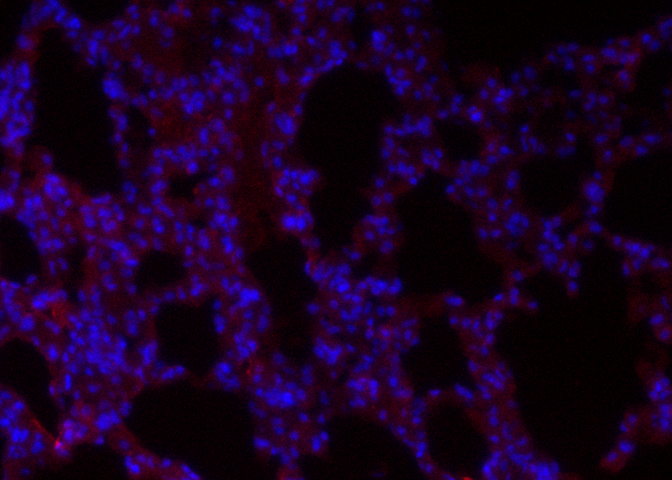

Supplement: Supplementary file 1 [file DataSheet1.ZIP › Supplementary materials/Original source data/uncropped images/Fig.6/Immunofluorescence/Fig.6I BLM-Merge.tif]

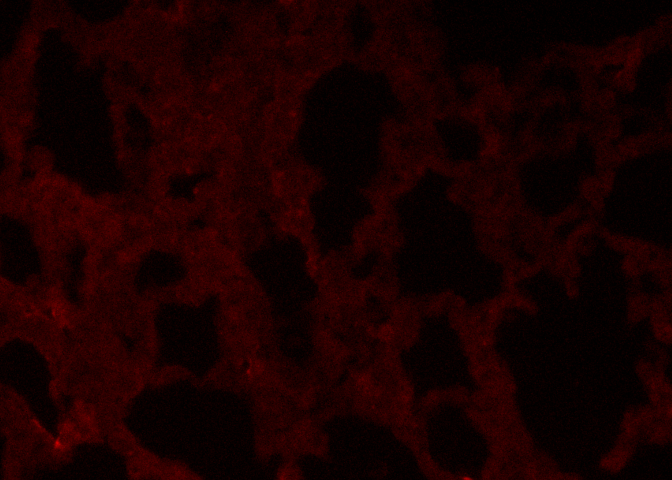

Supplement: Supplementary file 1 [file DataSheet1.ZIP › Supplementary materials/Original source data/uncropped images/Fig.6/Immunofluorescence/Fig.6I BLM-miR-130a-3p.tif]

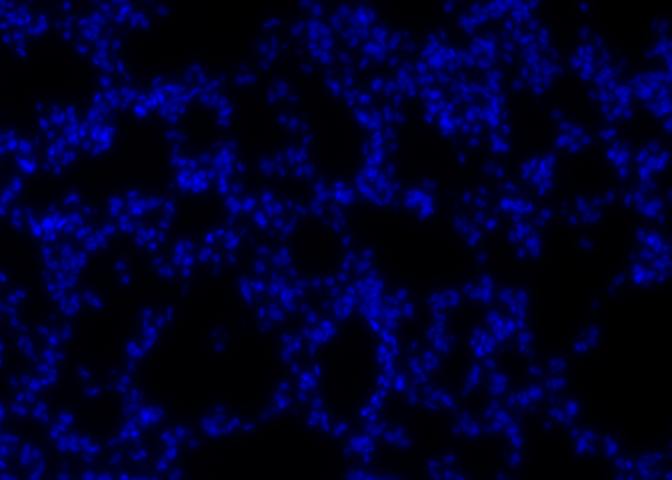

Supplement: Supplementary file 1 [file DataSheet1.ZIP › Supplementary materials/Original source data/uncropped images/Fig.6/Immunofluorescence/Fig.6I Control-DAPI.tif]

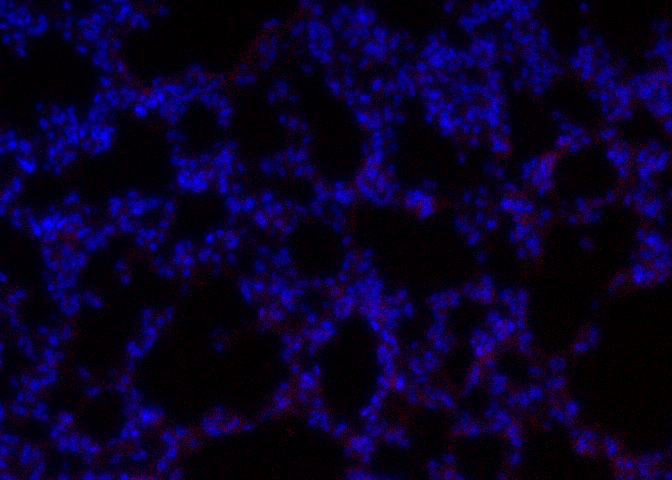

Supplement: Supplementary file 1 [file DataSheet1.ZIP › Supplementary materials/Original source data/uncropped images/Fig.6/Immunofluorescence/Fig.6I Control-Merge.tif]

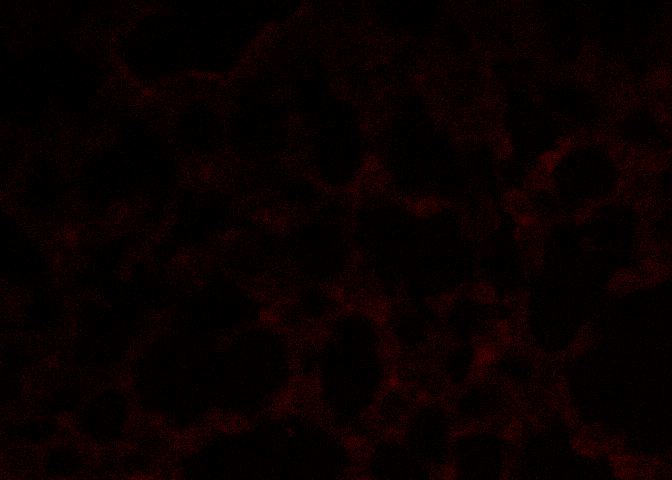

Supplement: Supplementary file 1 [file DataSheet1.ZIP › Supplementary materials/Original source data/uncropped images/Fig.6/Immunofluorescence/Fig.6I Control-miR-130a-3p.tif]

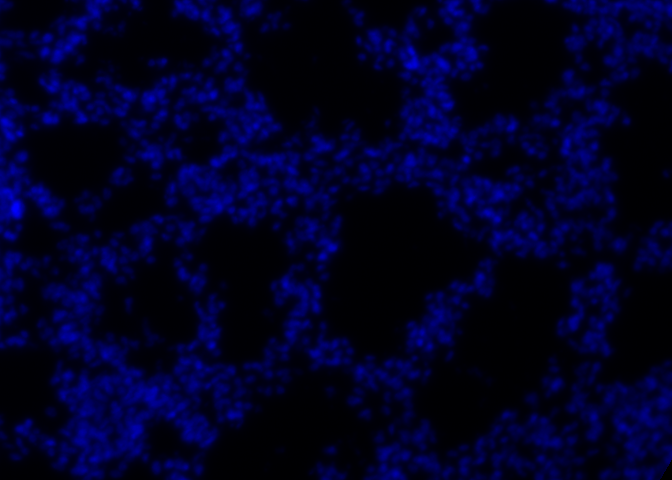

Supplement: Supplementary file 1 [file DataSheet1.ZIP › Supplementary materials/Original source data/uncropped images/Fig.6/Immunofluorescence/Fig.6J agomiR-DAPI.tif]

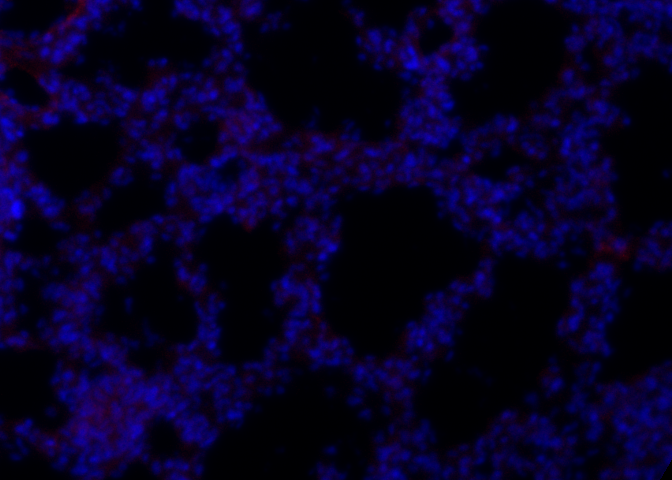

Supplement: Supplementary file 1 [file DataSheet1.ZIP › Supplementary materials/Original source data/uncropped images/Fig.6/Immunofluorescence/Fig.6J agomiR-Merge.tif]

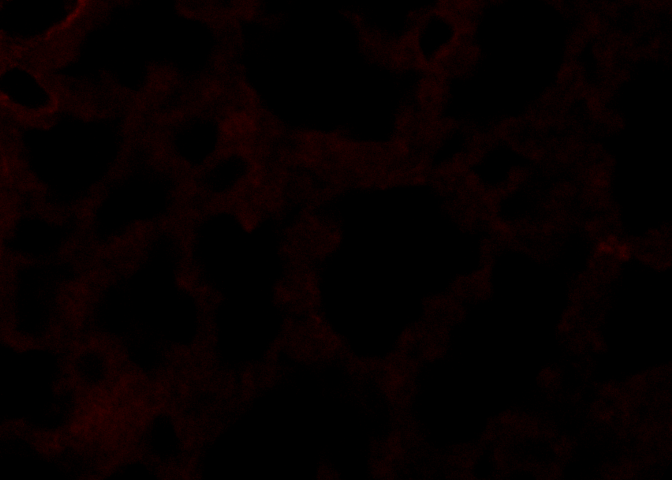

Supplement: Supplementary file 1 [file DataSheet1.ZIP › Supplementary materials/Original source data/uncropped images/Fig.6/Immunofluorescence/Fig.6J agomiR-miR-130a-3p.tif]

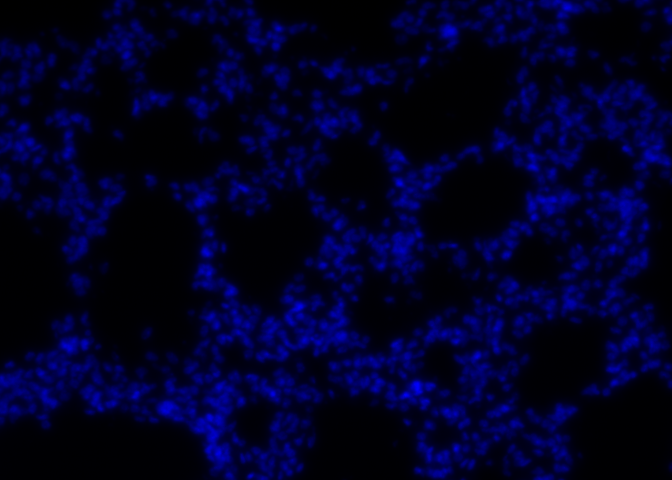

Supplement: Supplementary file 1 [file DataSheet1.ZIP › Supplementary materials/Original source data/uncropped images/Fig.6/Immunofluorescence/Fig.6J BLM+agomiR-DAPI.tif]

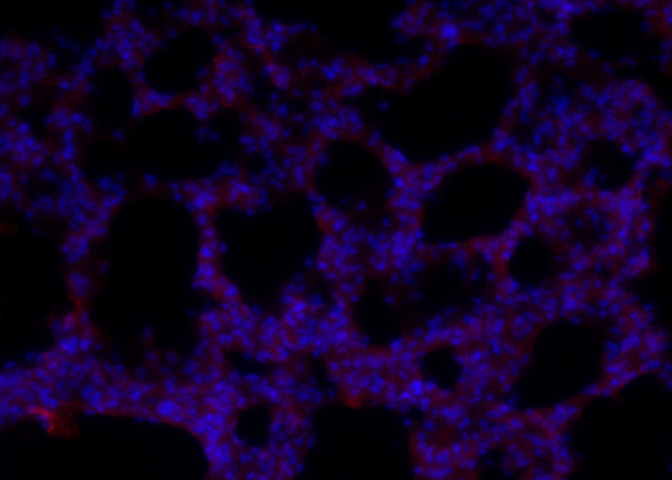

Supplement: Supplementary file 1 [file DataSheet1.ZIP › Supplementary materials/Original source data/uncropped images/Fig.6/Immunofluorescence/Fig.6J BLM+agomiR-Merge.tif]

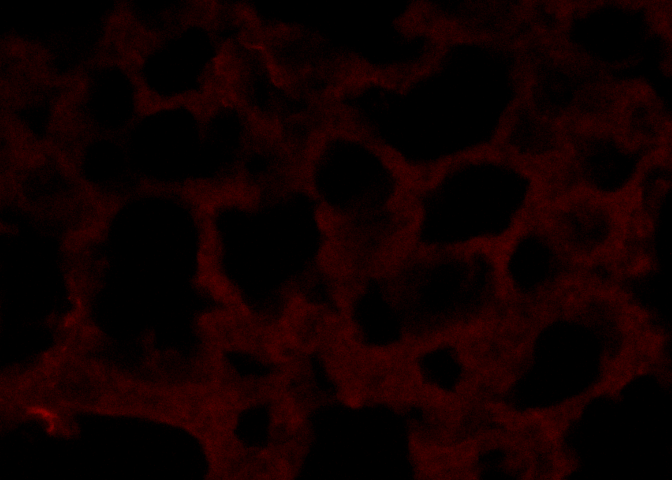

Supplement: Supplementary file 1 [file DataSheet1.ZIP › Supplementary materials/Original source data/uncropped images/Fig.6/Immunofluorescence/Fig.6J BLM+agomiR-miR-130a-3p.tif]

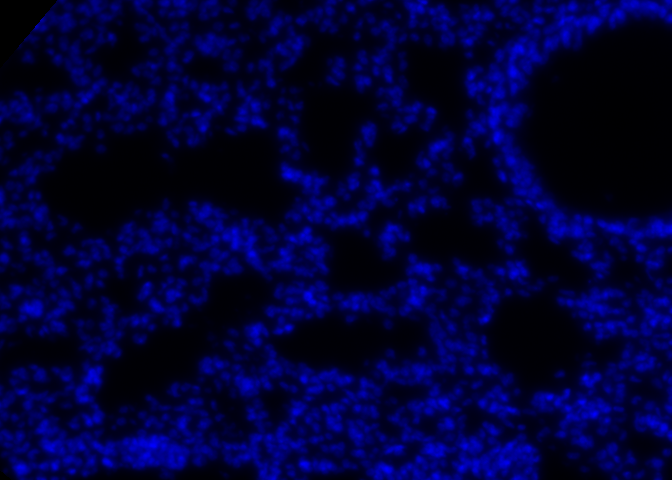

Supplement: Supplementary file 1 [file DataSheet1.ZIP › Supplementary materials/Original source data/uncropped images/Fig.6/Immunofluorescence/Fig.6J BLM-DAPI.tif]

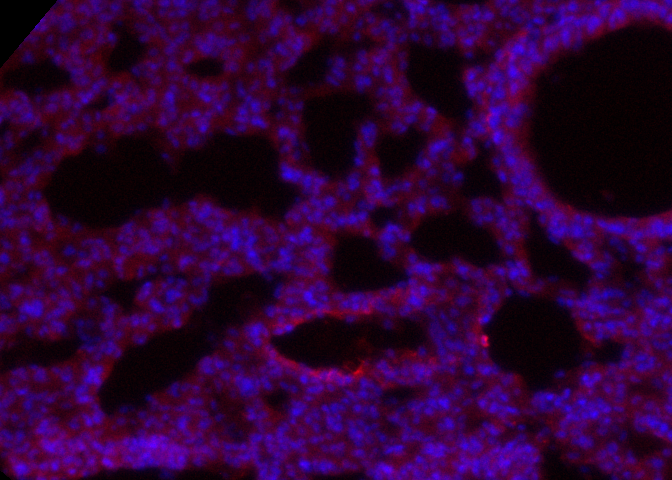

Supplement: Supplementary file 1 [file DataSheet1.ZIP › Supplementary materials/Original source data/uncropped images/Fig.6/Immunofluorescence/Fig.6J BLM-Merge.tif]

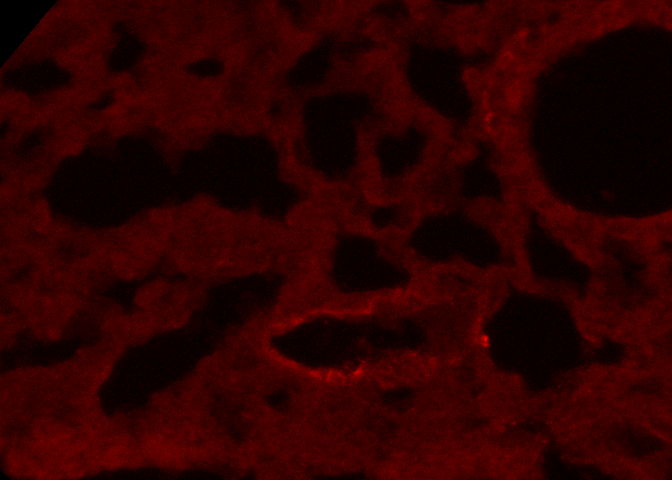

Supplement: Supplementary file 1 [file DataSheet1.ZIP › Supplementary materials/Original source data/uncropped images/Fig.6/Immunofluorescence/Fig.6J BLM-miR-130a-3p.tif]

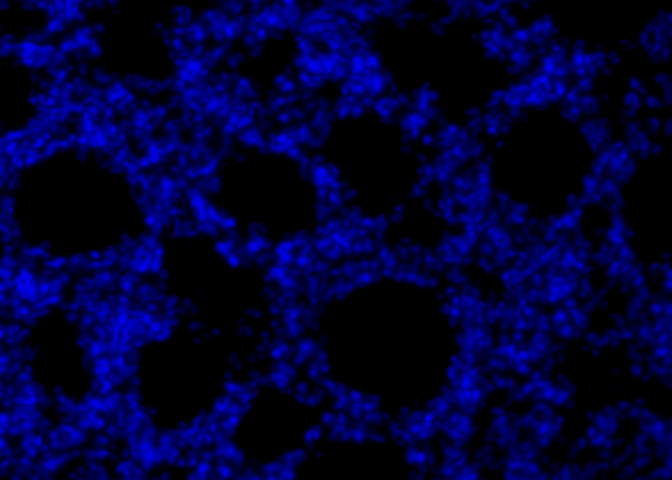

Supplement: Supplementary file 1 [file DataSheet1.ZIP › Supplementary materials/Original source data/uncropped images/Fig.6/Immunofluorescence/Fig.6J Control-DAPI.tif]

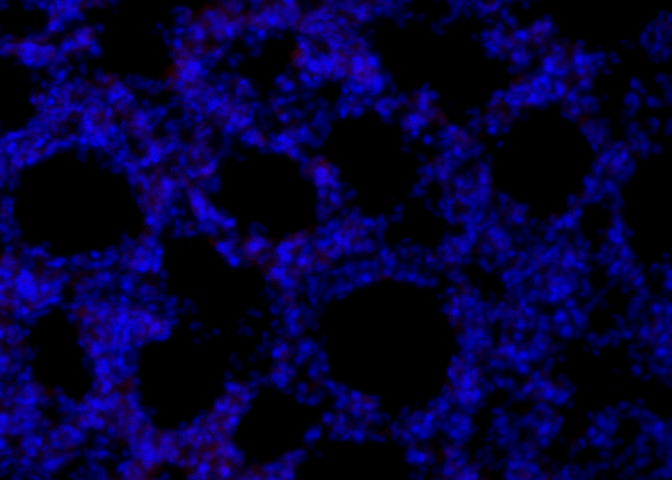

Supplement: Supplementary file 1 [file DataSheet1.ZIP › Supplementary materials/Original source data/uncropped images/Fig.6/Immunofluorescence/Fig.6J Control-Merge.tif]

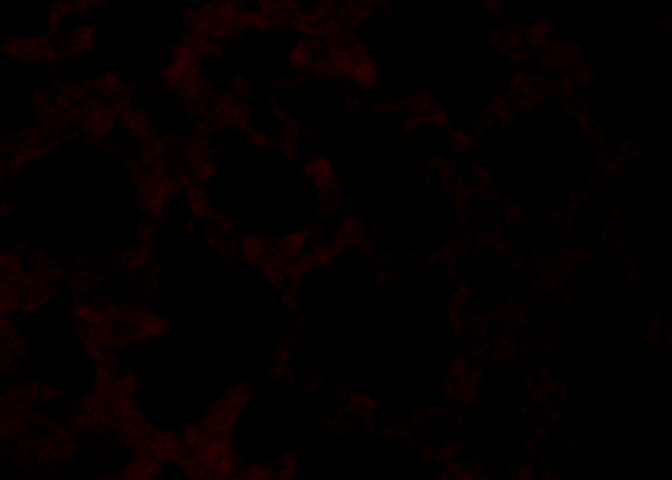

Supplement: Supplementary file 1 [file DataSheet1.ZIP › Supplementary materials/Original source data/uncropped images/Fig.6/Immunofluorescence/Fig.6J Control-miR-130a-3p.tif]

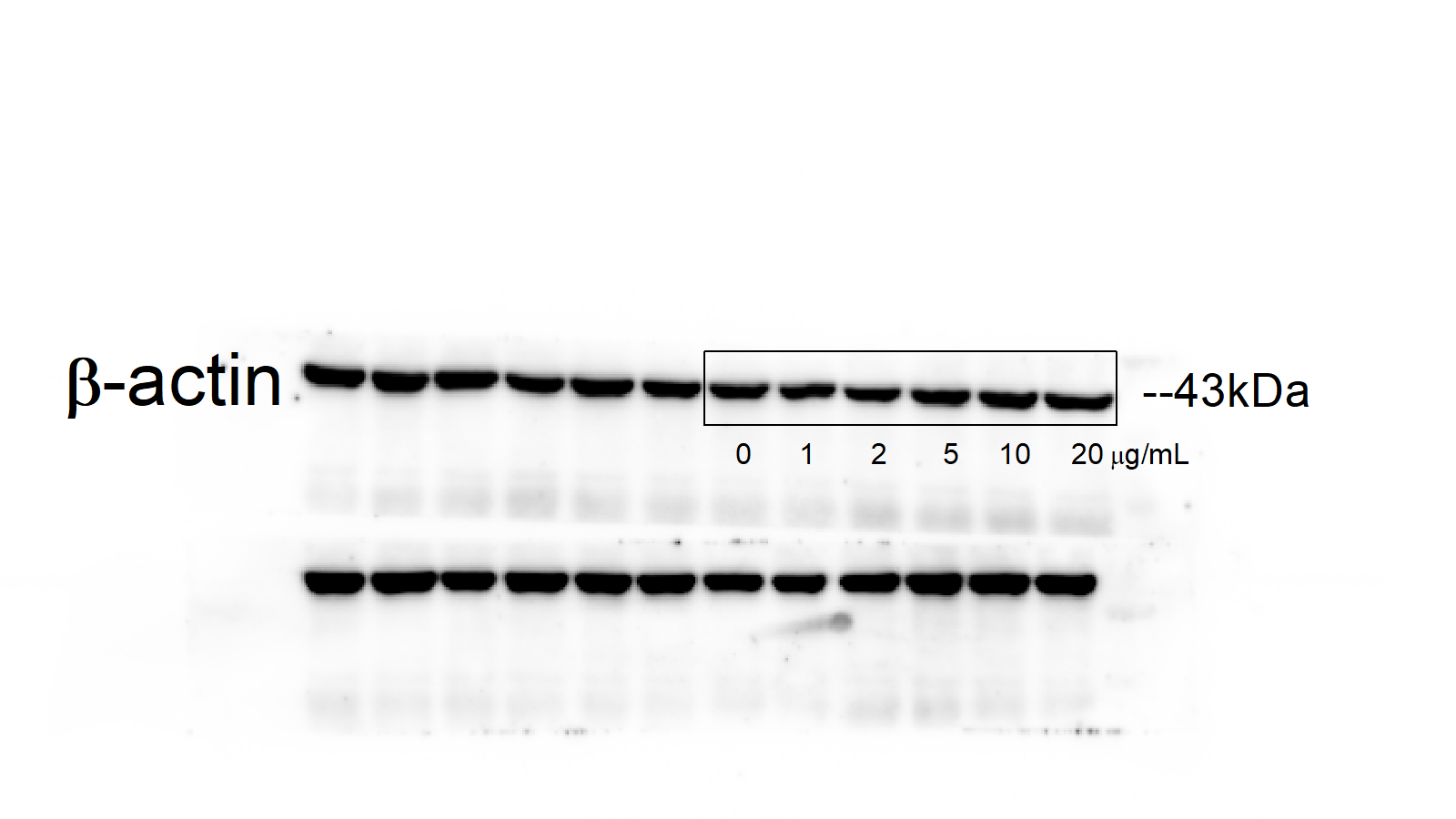

Supplement: Supplementary file 1 [file DataSheet1.ZIP › Supplementary materials/Original source data/uncropped images/Fig.7/Fig.7D b-actin.tiff]

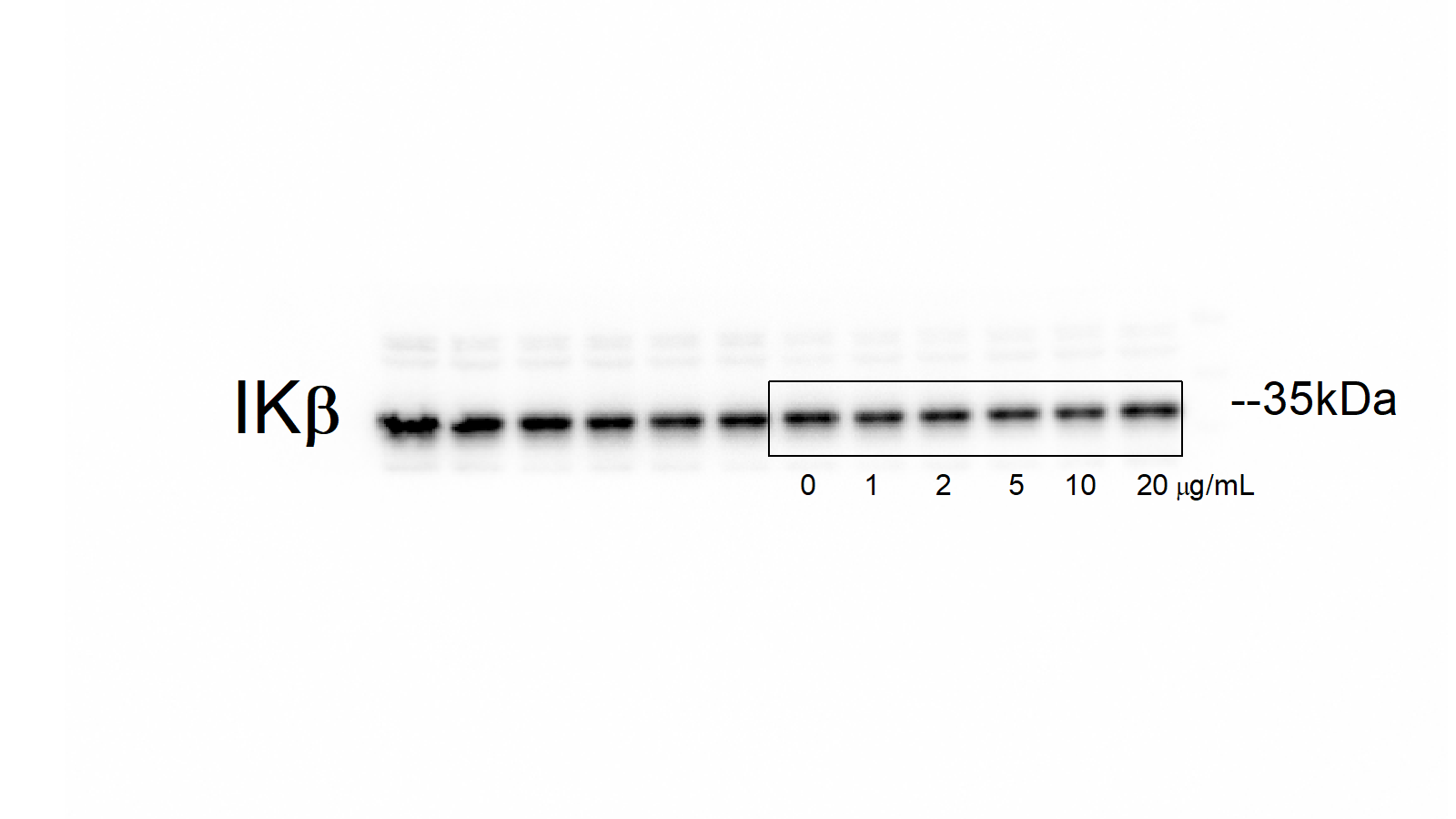

Supplement: Supplementary file 1 [file DataSheet1.ZIP › Supplementary materials/Original source data/uncropped images/Fig.7/Fig.7D IKb.tiff]

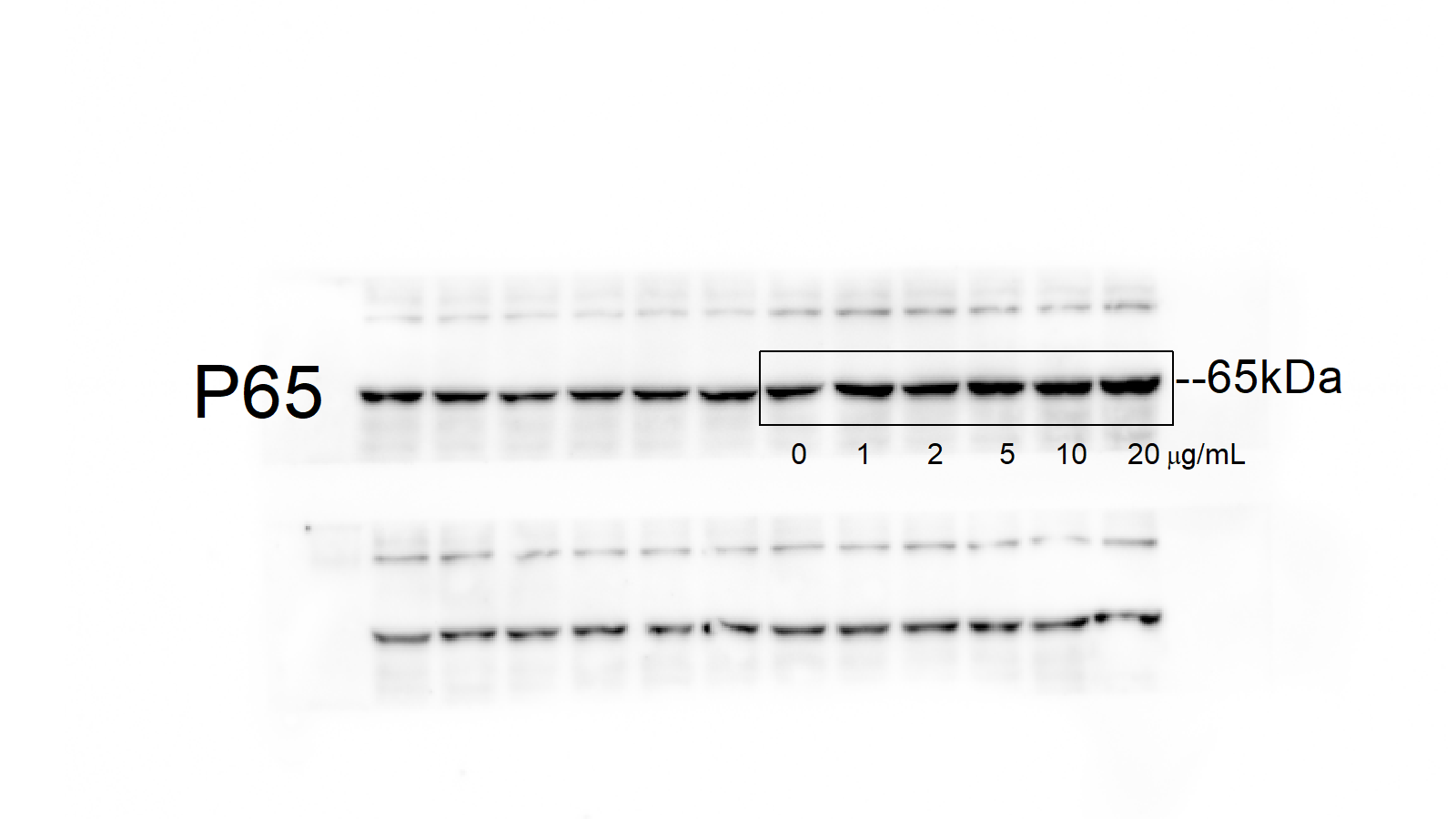

Supplement: Supplementary file 1 [file DataSheet1.ZIP › Supplementary materials/Original source data/uncropped images/Fig.7/Fig.7D P65.tiff]

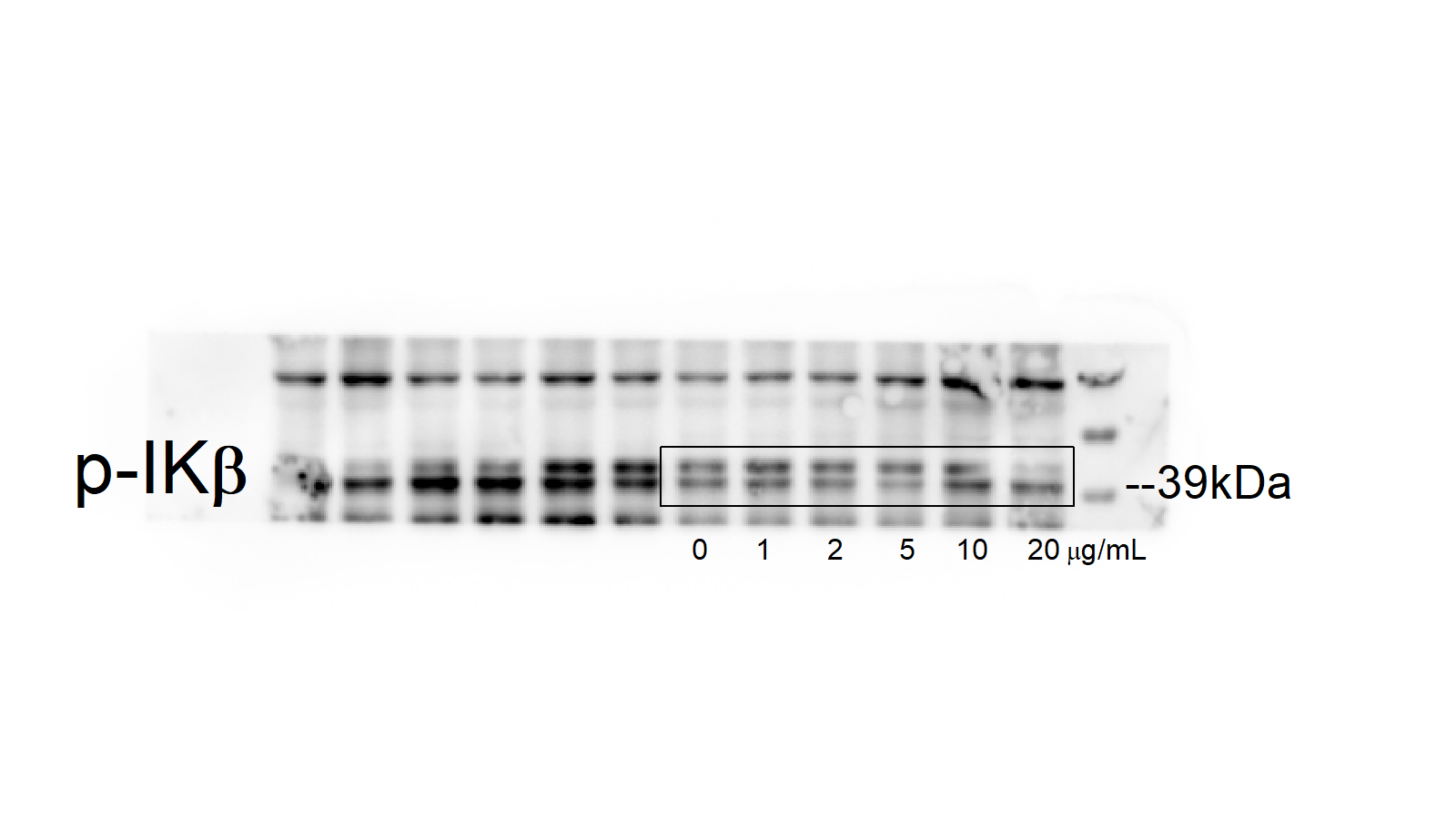

Supplement: Supplementary file 1 [file DataSheet1.ZIP › Supplementary materials/Original source data/uncropped images/Fig.7/Fig.7D p-IKb.tiff]

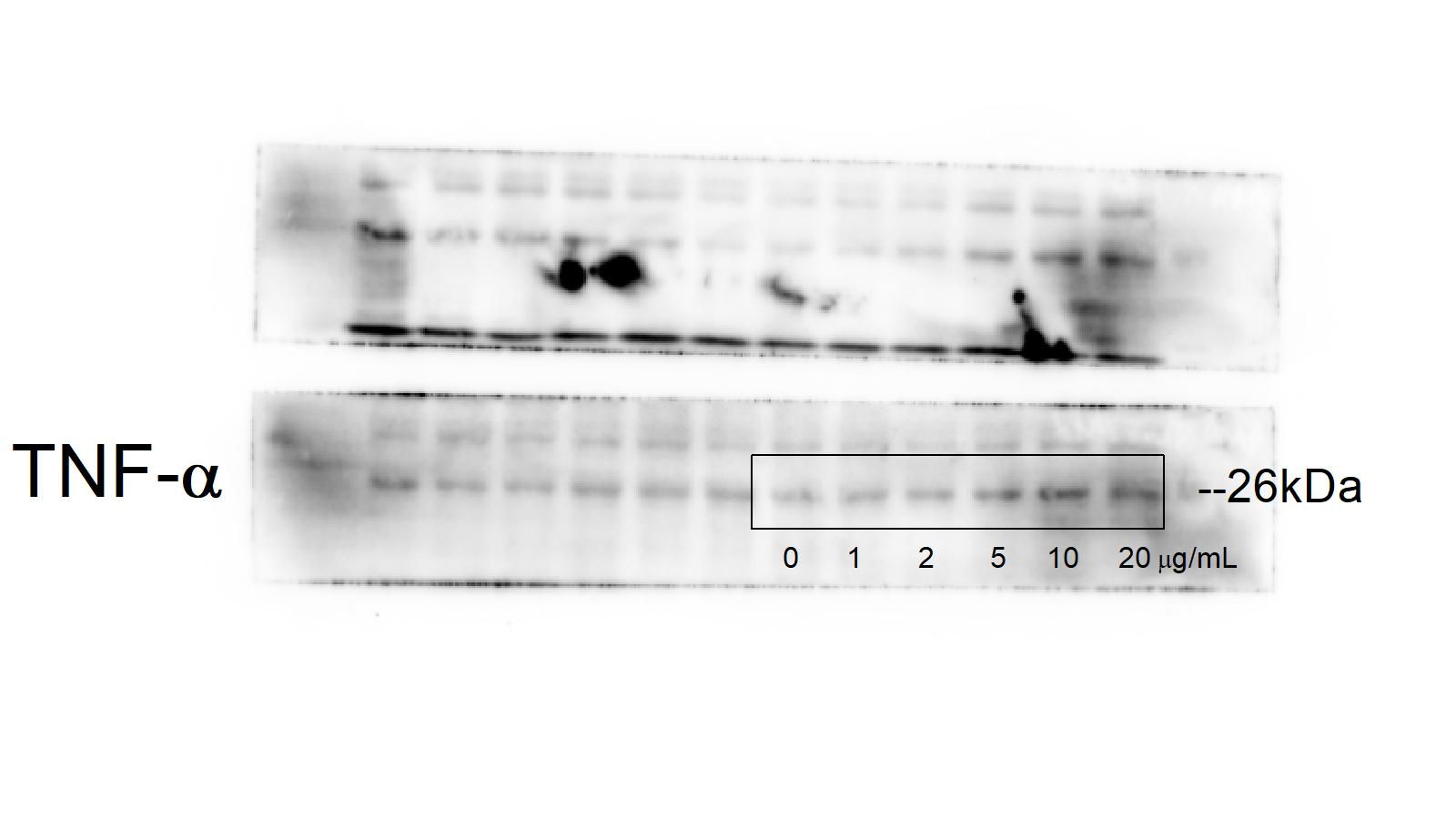

Supplement: Supplementary file 1 [file DataSheet1.ZIP › Supplementary materials/Original source data/uncropped images/Fig.7/Fig.7D TNF-a.tiff]

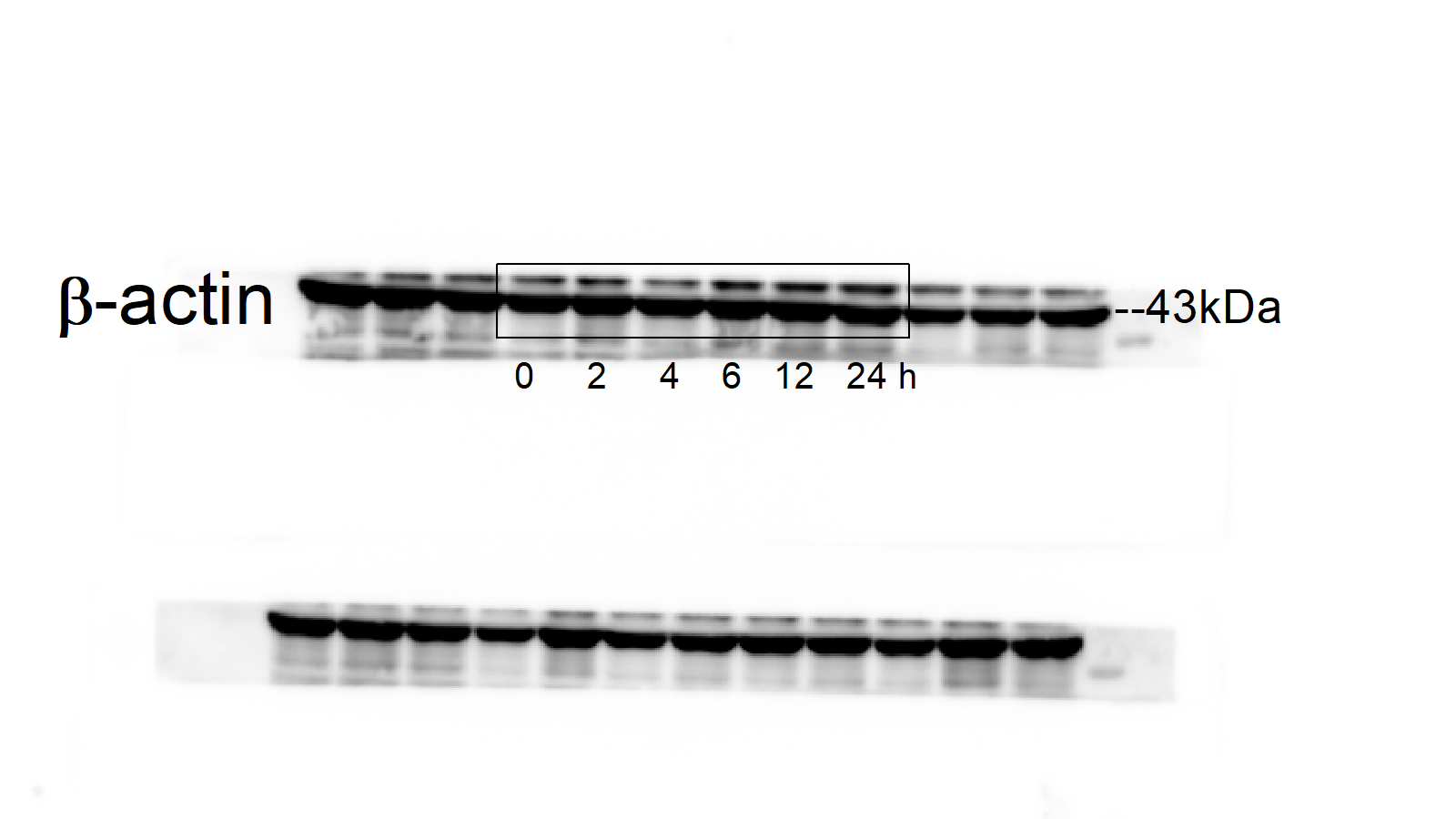

Supplement: Supplementary file 1 [file DataSheet1.ZIP › Supplementary materials/Original source data/uncropped images/Fig.7/Fig.7F b-actin.tiff]

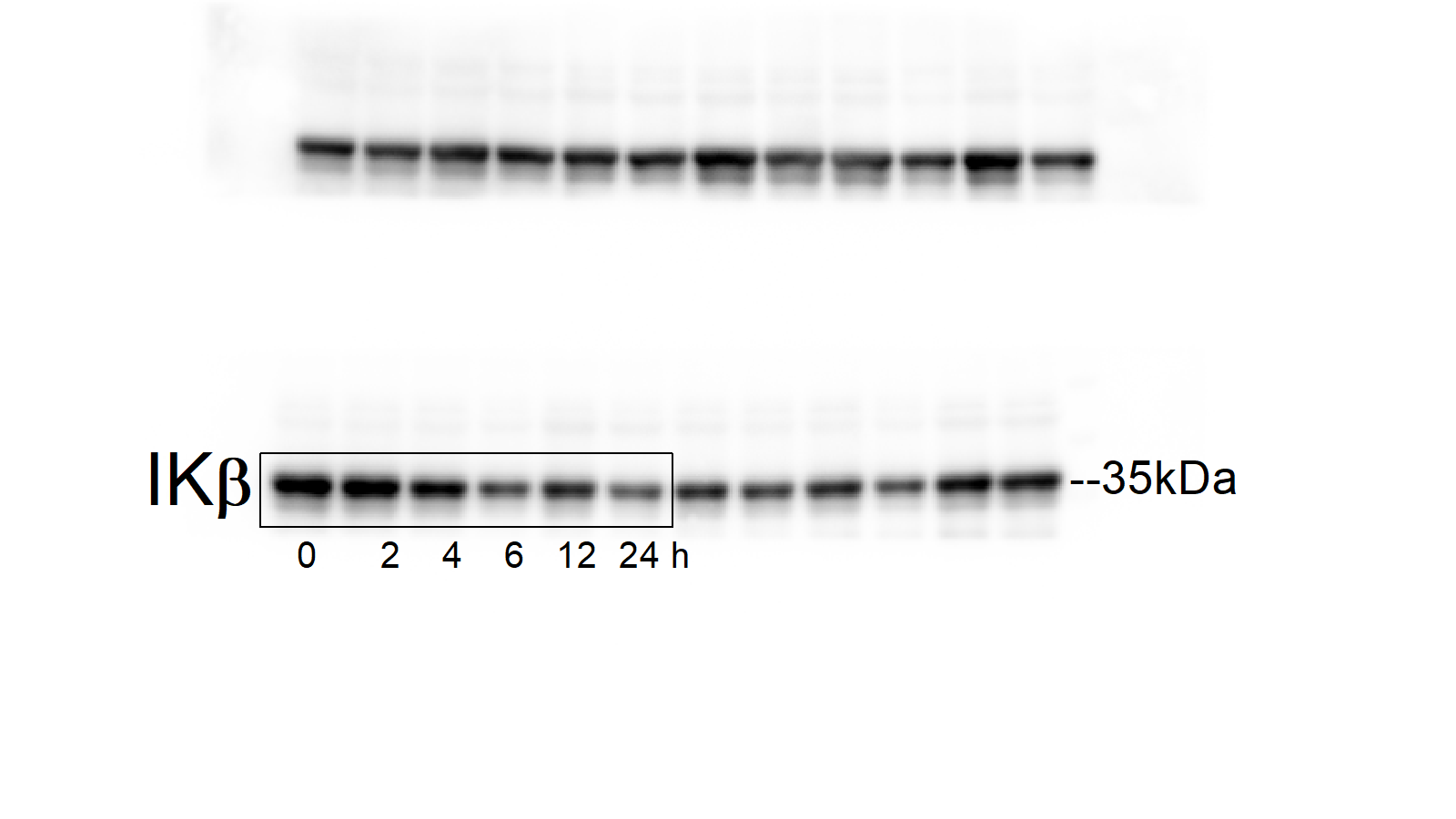

Supplement: Supplementary file 1 [file DataSheet1.ZIP › Supplementary materials/Original source data/uncropped images/Fig.7/Fig.7F IKb.tiff]

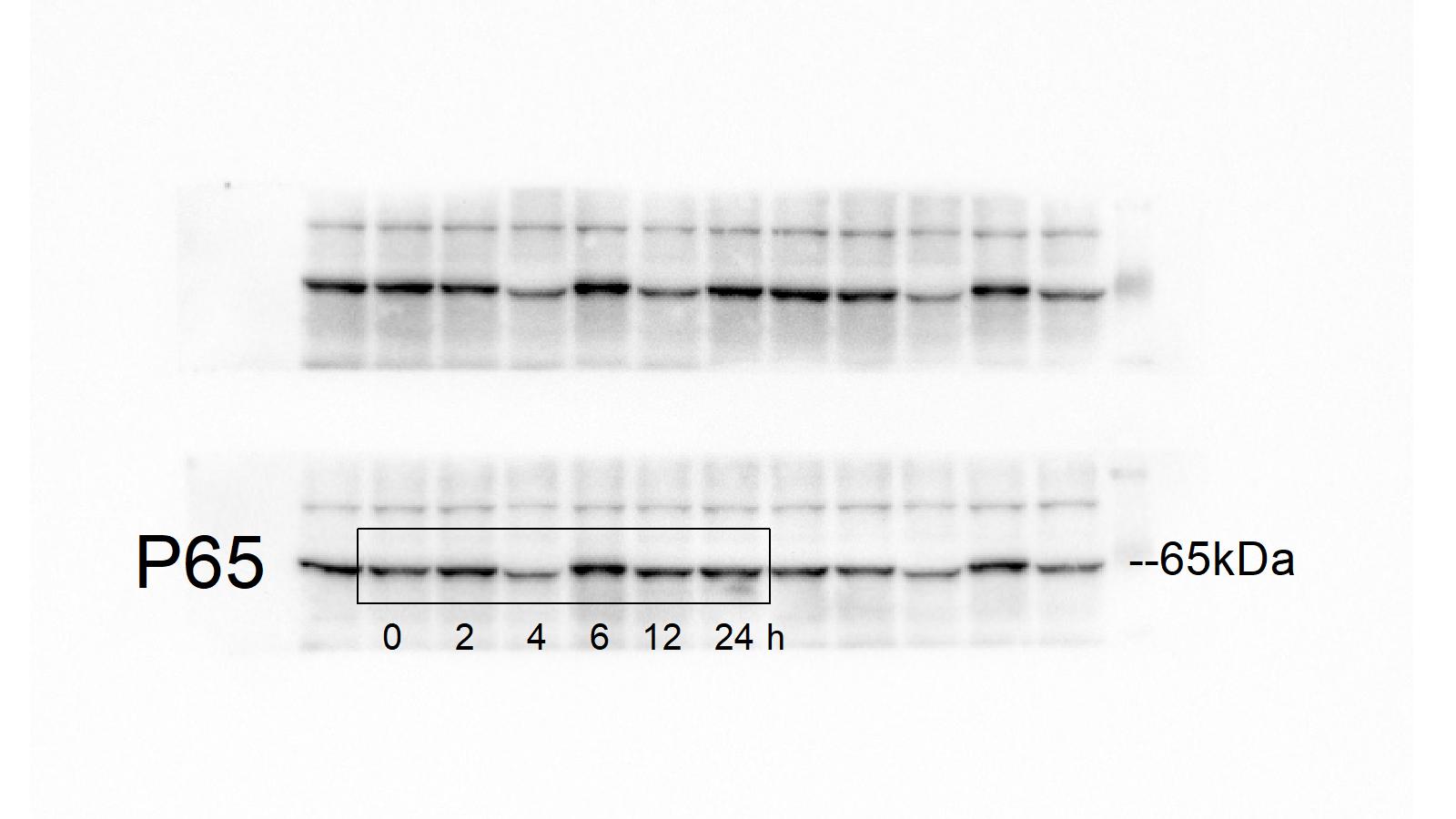

Supplement: Supplementary file 1 [file DataSheet1.ZIP › Supplementary materials/Original source data/uncropped images/Fig.7/Fig.7F P65.tiff]

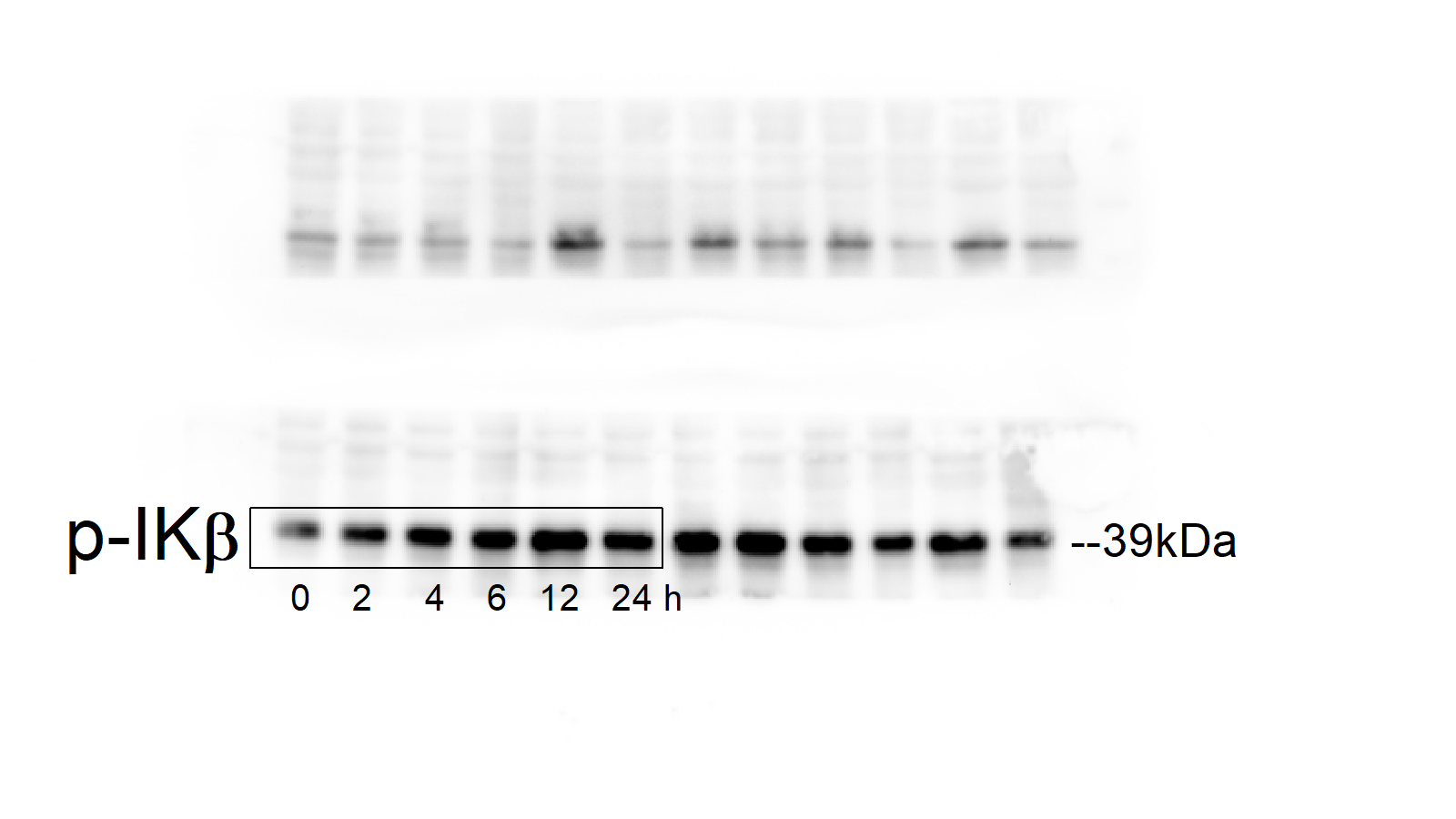

Supplement: Supplementary file 1 [file DataSheet1.ZIP › Supplementary materials/Original source data/uncropped images/Fig.7/Fig.7F p-IKb.tiff]

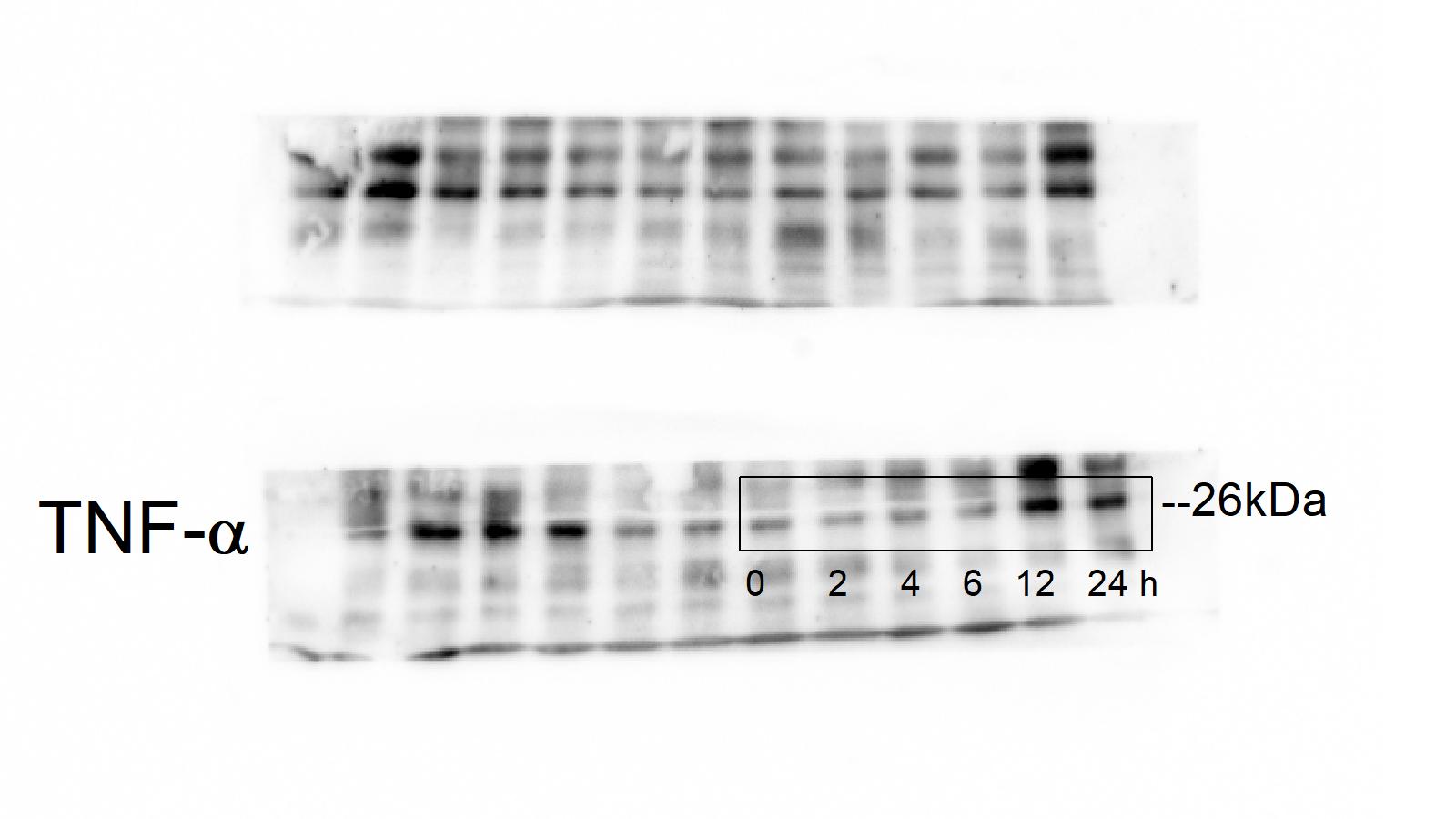

Supplement: Supplementary file 1 [file DataSheet1.ZIP › Supplementary materials/Original source data/uncropped images/Fig.7/Fig.7F TNF-a.tiff]

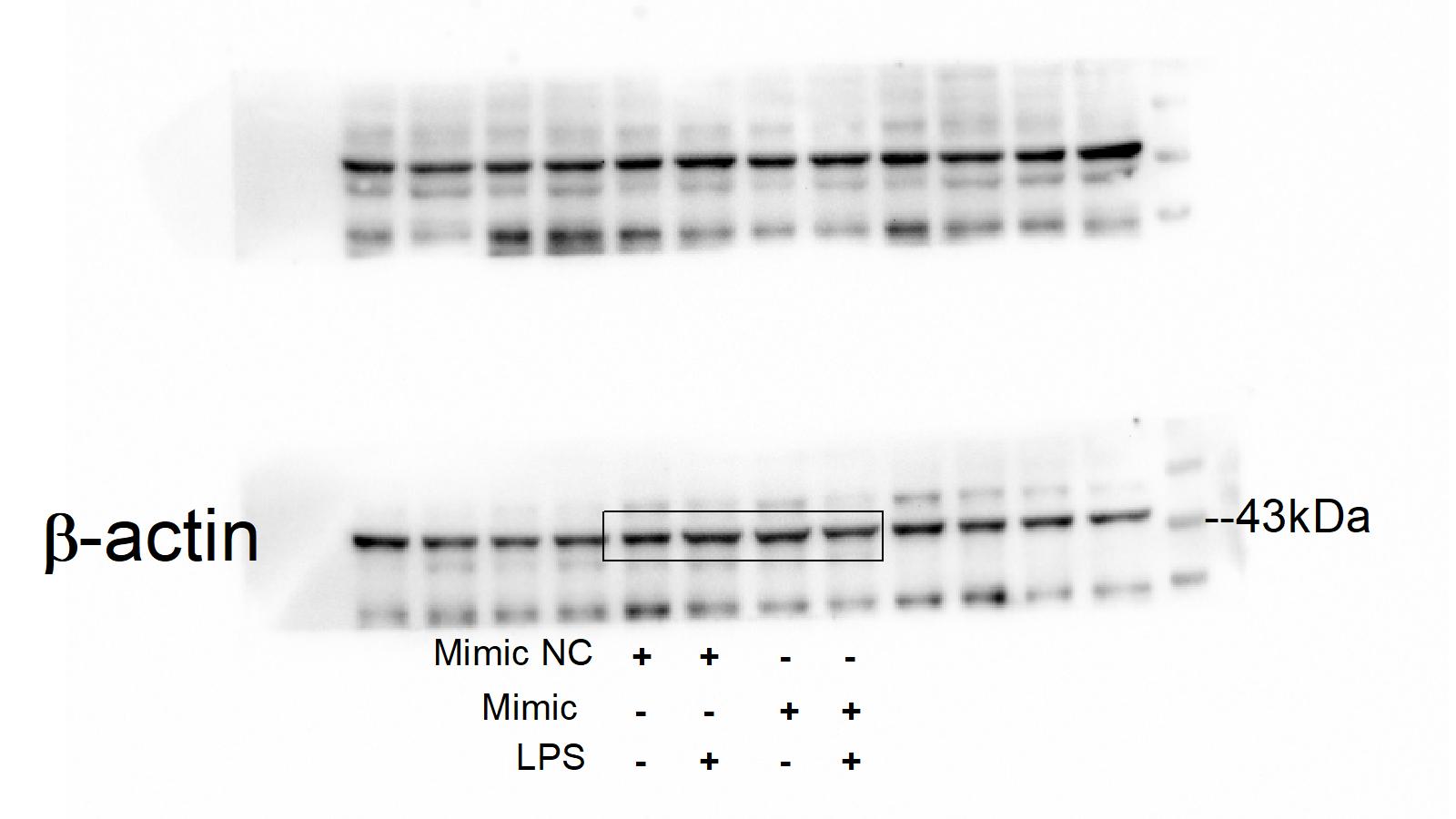

Supplement: Supplementary file 1 [file DataSheet1.ZIP › Supplementary materials/Original source data/uncropped images/Fig.7/Fig.7I b-actin.tiff]

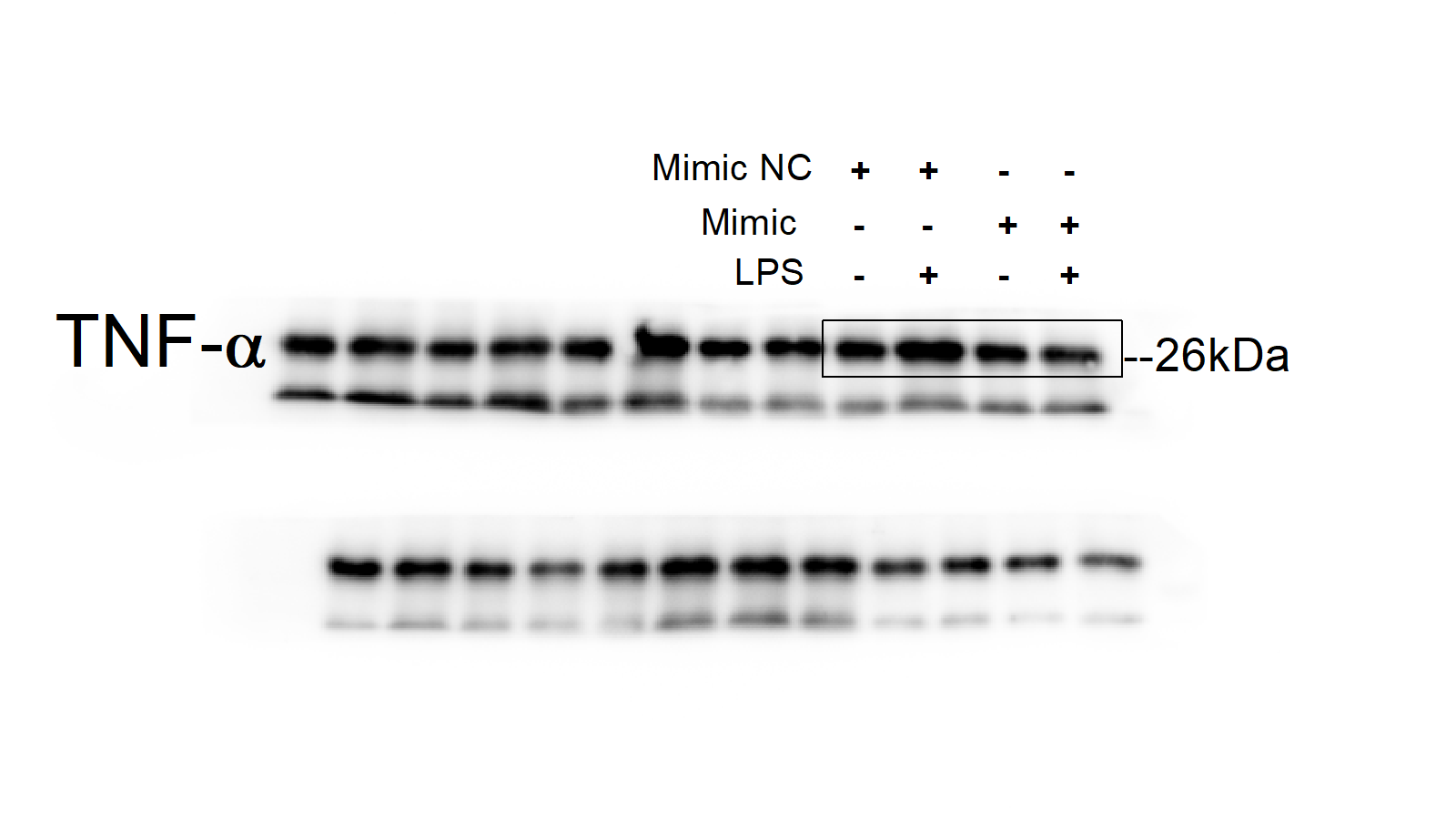

Supplement: Supplementary file 1 [file DataSheet1.ZIP › Supplementary materials/Original source data/uncropped images/Fig.7/Fig.7I TNF-a.tiff]

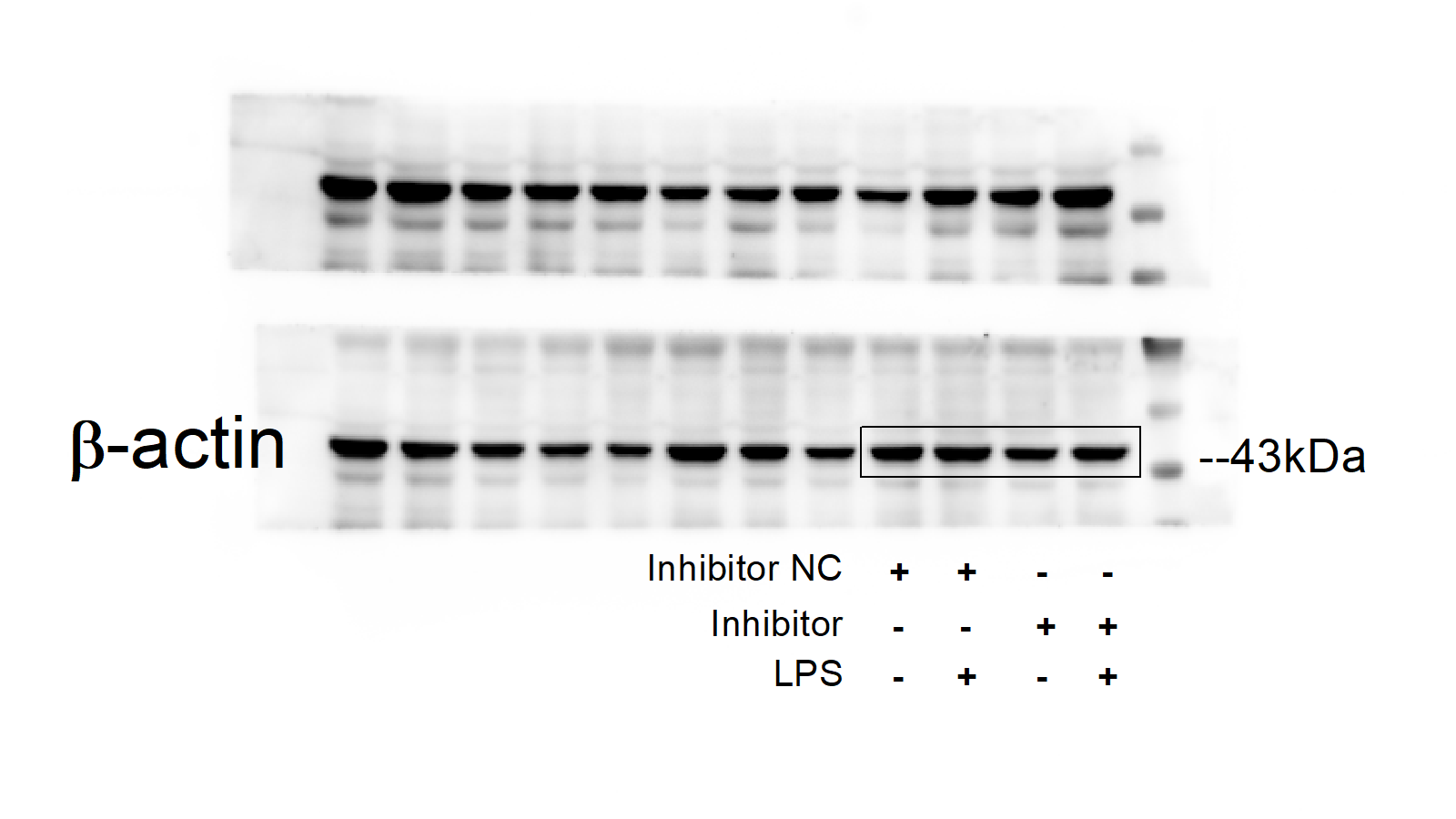

Supplement: Supplementary file 1 [file DataSheet1.ZIP › Supplementary materials/Original source data/uncropped images/Fig.7/Fig.7K b-actin.tiff]
